# Activity-dependent ribosome profiling reveals the landscape of canonical and non-canonical translation in brain tissue

**DOI:** 10.1038/s41467-026-74968-z

**Published:** 2026-07-23

**Authors:** Nayan Suryawanshi, Hitoshi Uchida, Ryo Endo, Kai Sato, Daisuke Satoh, Kazuya Tsumagari, Koshi Imami, Takayasu Mikuni, Motomasa Tanaka

**Affiliations:** 1 Laboratory for Protein Conformation Diseases, RIKEN Center for Brain Science, Wako, Saitama, Japan; 2 Department of Biomedical Sciences and Engineering, Graduate School of Medical and Dental Sciences, Institute of Science Tokyo, Tokyo, Japan; 3 Department of Cellular Neuropathology, Brain Research Institute, Niigata University, Niigata, Japan; 4 Department of System Pathology for Neurological Disorders, Brain Research Institute, Niigata University, Niigata, Japan; 5 Proteome Homeostasis Research Unit, RIKEN Center for Integrative Medical Sciences, Yokohama, Japan

**Keywords:** Cellular neuroscience, Gene expression analysis

## Abstract

Neural activity-dependent translation is essential for synaptic plasticity and diverse brain functions. Translation involves not only canonical main open reading frames (mORFs) but also upstream ORFs (uORFs), which may regulate mORF expression. However, due to technical limitations, systematic investigation of activity-dependent uORFs and mORFs in brain tissues remains challenging. Here, we developed a ribosome tagging and purification strategy that bypasses the prolonged turnover of ribosomal proteins, enabling ribosome profiling with one-hour temporal resolution after neural stimulation. Applying this strategy to mouse hippocampal slices undergoing long-term potentiation, we identify hundreds of activity-induced mORFs and uORFs, including a previously unknown uORF from *Egr1*. We demonstrate that this *Egr1*-uORF translation is tightly regulated by neuronal activity, and its encoded peptide interacts with peroxisomal machinery, suggesting a potential link between synaptic stimulus and peroxisome biology. This study provides a useful technique and resources for deciphering molecular mechanisms underlying activity- and translation-dependent brain functions in health and disease.

## Abstract (web summary)

Investigation of activity-dependent protein synthesis selectively in activated neurons has long been a challenge. Here, the authors developed a technique to avoid the translational background from inactive neurons and uncovered previously unknown activity-dependent alternative translation with functional implication, such as the uORF of Egr1.

## Introduction

Neural activity-dependent protein synthesis is essential for synaptic plasticity and circuit remodeling, serving as the basis for higher-order brain functions such as learning and memory^1–3^. Emerging evidence indicates that this process extends beyond canonical main open reading frames (mORFs) to include non-canonical translation, such as upstream ORFs (uORFs), which are translated in response to neural activity and may exert significant regulatory effects on downstream protein expression^4^. uORFs have garnered increasing attention for their role in translational control, with numerous genes involved in synapse formation and axon development now recognized to contain actively translated uORF^4,5^. However, our understanding of which specific uORFs are translated during neural activation in brain tissues and how they influence neuronal function remains limited, primarily because of the difficulty of systematically detecting these short, often transient peptides.

Existing methods for studying activity-dependent protein synthesis are limited in their ability to address this question. While mass spectrometry (MS) enables proteome-wide analysis, it typically requires large sample volumes, making it impractical to isolate and analyze the small fraction of neurons activated within heterogeneous tissues^4–6^. Additionally, the transient translation of short peptides, such as those derived from uORFs, poses a technical challenge for MS detection. Although RNA-seq provides a comprehensive view of transcription, it does not necessarily capture translation dynamics and may underrepresent non-canonical ORFs because it primarily corresponds to canonical ORFs. Moreover, since non-neuronal dividing cells in brains show a higher translation level than non-dividing neurons, data obtained from RNA-seq or MS analysis of large brain tissue might include a greater contribution from non-neuronal cells^5^. Although neuronal primary culture systems in vitro can overcome this issue, they might have relatively less biological relevance due to the lack of diverse, complex cell-cell interactions present in vivo^4,7,8^. Ribosome profiling (Ribo-seq), which maps ribosome occupancy along the transcripts, offers a direct approach to detecting both canonical and non-canonical ORFs^9,10^. However, applying Ribo-seq specifically to neurons that have recently undergone increased activity in brain tissues is challenging, as current techniques for isolating activated neurons may require prohibitively large tissue samples or impair activity-dependent translation due to the prolonged handling time^11–13^^.^

Translating ribosome affinity purification (TRAP), which uses a tagged ribosomal protein expressed under cell-type-specific promoters, has provided valuable insights into translational profiles ^14,15^^.^ Importantly, however, selectively capturing ribosomes in activated neurons within a short time window remains challenging (Supplementary Fig. 1a). While immediate-early gene promoters can drive the expression of tagged ribosomal proteins to label translating ribosomes in activated neurons, this approach is not suited for studying translation within hours of neural stimulation. The long half-life of ribosomal proteins such as L10a, which remain stably integrated into existing ribosomes for several days, limits the incorporation of newly synthesized EGFP-L10a into functional ribosomes over short timescales, resulting in insufficient tagging (Supplementary Fig. 1a)^16–18^. In fact, although previous studies detected upregulated translation of many genes after neural stimulation, well-known IEGs such as *Arc*, *Fos*, and *Egr1* were not necessarily enriched upon stimulation, possibly due to the lack of activated-neurons specificity, cell-type specificity, and/or neuron-glia interactions present in brain tissues (Supplementary Fig. 1b–d)^5,19^^.^ Consequently, no comprehensive Ribo-seq-based analysis has specifically focused on recently activated neurons in brain tissues to identify both mORFs and uORFs, as well as their potential regulatory functions in neuronal activity.

To overcome these limitations, we developed a strategy to selectively target ribosomes in recently activated neurons with high temporal specificity. For this aim, we first pre-labeled ribosomes in neurons with EGFP-L10a. Then, to specifically mark these ribosomes in activated neurons, we used an adeno-associated virus (AAV) encoding an EGFP nanobody^20^ fused to HaloTag under the activity-dependent E-SARE promoter^21^^.^ Upon stimulation, this fusion protein is expressed exclusively in activated neurons, binding to EGFP-L10a-labeled ribosomes. These ribosomes are then isolated via HaloTag-based purification for Ribo-seq analysis. By circumventing the long turnover time of newly synthesized ribosomal proteins^16,17^^,^ this activity-dependent Ribo-seq technique enables us to profile both mORFs and uORFs in activated neurons with high temporal specificity.

Applying this approach to mouse hippocampal organotypic slices following long-term potentiation (LTP) induction, we identified several hundred canonical mORFs and uORFs whose translation was significantly upregulated at one hour after stimulation. Notably, activity-induced and integrated stress response (ISR)-induced uORFs show substantial overlap. We demonstrated a previously unknown activity-dependent uORF from *Egr1*, which is also tightly regulated by endoplasmic reticulum stress. The peptide derived from *Egr1*-uORF contains a highly conserved C-terminal peroxisomal targeting signal (PTS1) and interacts with peroxisomal machinery, suggesting a potential link between synaptic stimulus and peroxisomal biology. Collectively, this study provides a valuable technical platform for capturing a transient translational snapshot from activated neurons in brain tissues and presents a comprehensive resource of immediate early genes (IEGs), including both mORFs and uORFs. Therefore, our results will provide a direction to elucidate the molecular mechanisms underlying activity- and translation-dependent brain functions in health and disease.

## Results

### Development of a method to investigate activity-dependent translation

**Fig. 1:**
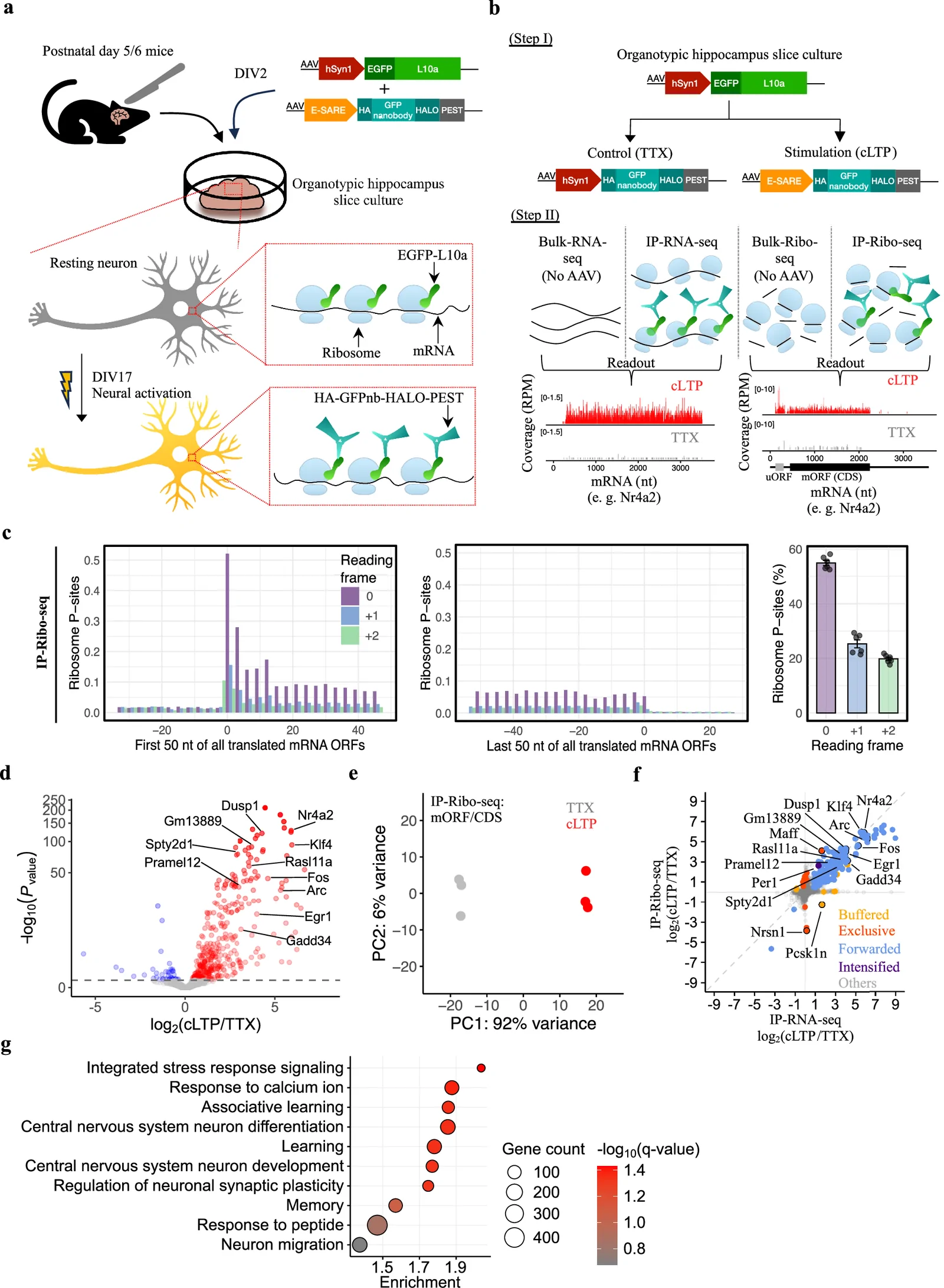
Development of an activated-neuron–specific ribosome footprinting analysis reveals previously unrecognized immediate early genes. **a** Schematic summarizing the workflow of the method used to investigate activity-dependent translation. Promoters are highlighted in red or orange, expression sequences of the constructs are shown in shades of green, and proteasome-targeting sequences for the clearance of unbound background EGFP nanobodies (GFPnb) are shown in gray. **b** Methodology of activity-dependent Ribo-seq (IP–Ribo-seq) and RNA-seq (IP–RNA-seq), as well as total lysate sequencing (Bulk–Ribo-seq and Bulk–RNA-seq). uORF denotes an upstream open reading frame, and mORF denotes the main canonical open reading frame (also known as the CDS). **c** Bar plots displaying P-sites derived from IP–Ribo-seq reads within the first 50 nt (left), the last 50 nt of annotated ORFs (middle), and the percentage of footprints in each reading frame (right). *n*  =  6; data are shown as mean ± s.e.m. **d** A volcano plot shows differential expression analysis between 1 h cLTP and TTX from three independent replicates. Red and blue points indicate significantly upregulated and downregulated genes, respectively (*P*  <  0.01; DESeq2; two-sided Wald test with multiple-testing correction using the Benjamini–Hochberg method (FDR)). **e** Principal component analysis (PCA) of IP–Ribo-seq data showing that TTX-treated samples (*n*  =  3, gray) and cLTP-treated samples (*n*  =  3, red) form two distinct clusters. **f** Translation efficiency scatterplots showing activity-dependent fold changes between cLTP and TTX for canonical CDSs (mORFs). Blue points indicate forwarded expression (*n*  =  221), magenta indicate intensified expression (*n*  =  1), ora*n*ge indicate exclusive expression (*n*  =  16), a*n*d yellow indicate buffered expression of canonical ORFs (*n*  =  10) (IP-Ribo-seq, *n*  =  3; IP-RNA-seq, *n* = 2). **g** Gene set enrichment analysis (GSEA) was performed on DESeq2-normalized IP–Ribo-seq expression counts of mORFs (CDSs) from TTX-treated (*n*  =  3) and cLTP-treated (*n*  =  3) samples. Enriched pathways are shown; FDR *q*  <  0.25.

To develop an activity-dependent Ribo-seq method, IP-Ribo-seq, we engineered AAV-based systems to selectively label ribosomes in recently activated neurons. Specifically, we designed two types of AAV constructs: one to pre-label ribosomes in neurons with EGFP-L10a (hSyn1::EGFP-L10a), driven by the constitutive neuron-specific human Synapsin1 (hSyn1) promoter, and the other expressing an EGFP nanobody (hSyn1::EGFP nanobody-HaloTag-PEST or E-SARE::EGFP nanobody-HaloTag-PEST) capable of binding to EGFP-L10a-labeled ribosomes. We leveraged the synthetic E-SARE promoter, designed using *Arc* enhancer and promoter^18,21^, to drive activity-induced expression of EGFP nanobody^20^^,^ specifically in excited neurons (Supplementary Fig. 2a–d)^21^^,^ while hSyn1 drove constitutive expression in all neurons. To enhance the labeling specificity toward translating ribosomes, we included a PEST sequence to accelerate the degradation of unbound EGFP nanobody-HaloTag. Given that newly synthesized EGFP-L10a requires over a week to be incorporated into ribosomes following AAV infection^16^^,^ we transduced hippocampal slice cultures at 2 days in vitro (DIV 2) and allowed 2 weeks for sufficient incorporation of EGFP-L10a before inducing chemical LTP. For the experimental setup, we transduced one group of slices designated for cLTP treatment with hSyn1::EGFP-L10a and E-SARE::EGFP nanobody-HaloTag-PEST, while a control group received hSyn1::EGFP-L10a and hSyn1::EGFP nanobody-HaloTag-PEST, both at DIV 2. Then, at DIV 17, when EGFP-L10a is incorporated into neuronal ribosomes, control slices were treated with tetrodotoxin (TTX), whereas cLTP slices were exposed to a cocktail of forskolin, rolipram, bicuculline, and 4-aminopyridine^22^^.^ In both groups, EGFP-L10a expression was driven by the hSyn1 promoter to ensure EGFP-tagging of ribosomes in all neurons (Fig. 1a). In the TTX control group, GFP nanobody-Halo-PEST was also expressed constitutively under the hSyn1 promoter, whereas in the cLTP group, its expression was restricted to activated neurons by the E-SARE promoter (Fig. 1b). Since the nanobody binds specifically to EGFP-L10a, fluorescent imaging confirmed selective labeling of neurons activated by cLTP, demonstrating the effectiveness of this approach in identifying ribosomes in recently stimulated neurons (Supplementary Fig. 2e). Next, the slices from both TTX and cLTP groups were then subjected to immunoprecipitation using Halo-trap beads and processed for Ribo-seq. As detailed in Supplementary Fig. 2f, IP-Ribo-seq utilizes the E-SARE promoter, which incorporates activity-dependent enhancers and the promoter region of *Arc*^21^. Consistently, the brain cell-type-specific expression analysis from Furlanis et al. (2019) demonstrates the enrichment of *Arc* in activity-prone CaMKII^+^ neurons (Supplementary Fig. 2g)^23^. These results suggest that E-SARE-driven expression is likely restricted to excitatory neurons undergoing neuronal activity (Supplementary Fig. 2). Furthermore, the analysis of the ribosome-protected fragments (RPFs) from IP-Ribo-seq revealed a significantly higher proportion originating from neurons compared to non-neuronal cells, as determined by reference to neuron-specific gene expression data from the Allen Brain Map scRNA-seq database (Supplementary Fig. 2h)^24^. Together, these findings demonstrate that our technique enables the rapid and exclusive capture of the translational landscape in recently activated neurons.

To validate the specificity of the IP-Ribo-seq approach, we compared it with conventional Bulk-Ribo-seq, which lacks specificity regarding neuronal cell type or activation states (Fig. 1b, Supplementary Fig. 2f). Notably, the rRNA content (~70–80%) across Ribo-seq strategies was comparable to previous brain-derived datasets (Supplementary Fig. 3a, b)^5^. Additionally, all Ribo-seq datasets displayed the characteristic alignment enriched to the coding region (CDS) of mRNA (Supplementary Fig. 3c, d), RPFs length distribution (Supplementary Fig. 3e, f), and three-nucleotide periodicity of active translation (Fig. 1c, Supplementary Fig. 3g), facilitating robust ORF identification. Comparative clustering analysis confirmed that the IP-Ribo-seq architecture substantially enhances the resolution of activity-dependent translation profiles over conventional Bulk-Ribo-seq, particularly in Cluster 1 (Supplementary Fig. 4a). Furthermore, the predominant enrichment of activity-dependent transcripts corresponding to neuronal cell type within this cluster validates the specificity of the IP-based method for activated neurons (Supplementary Fig. 4b)^25^.

To evaluate the efficacy of the IP-Ribo-seq method, we first examined differential gene expression between cLTP and control TTX-treated cultured hippocampal slices (Fig. 1d, e). Remarkably, we identified a total of 399 actively upregulated mORFs, mapping to 271 distinct genes, including well-known IEGs, such as *Fos*, *Arc*, and *Nr4a2*, in response to cLTP treatment (log_2_FC > 0 and *P*_*adj*_ < 0.05). The PCA analysis of IP-Ribo-seq revealed that the TTX and cLTP sample groups showed well-separated clusters, indicating the underlying differences by neuronal stimulation (Fig. 1e).

To investigate distinct translational regulation of mORF upon neuronal activity, we calculated translational efficiency (TE)^26^ by comparing differential expression in activity-dependent IP-Ribo-seq and IP-RNA-seq (Fig. 1b, f). Following established frameworks, we categorized the differentially regulated mORFs into four groups based on the relationship between mRNA abundance and translational output^27–29^. These include forwarded (both translation up and mRNA up); intensified (translation up and mRNA down or vice versa); buffered (either translation changes negated by mRNA changes or vice versa), and exclusive genes (either translation up or mRNA up) (Supplementary Fig. 5a, d).

Unlike Bulk-RNA-seq, IP-RNA-seq enabled the identification of transcriptional changes specific to active neurons. However, as IP-RNA-seq does not capture the free-floating mRNA pool, we also calculated TE by comparing IP-Ribo-seq against conventional Bulk-RNA-seq, which includes contributions from inactive neurons and non-neuronal cells (Fig. 1b, Supplementary Fig. 5b, c). The regulatory groups remained largely consistent between methods, such as identification of 221 and 242 forwarded mORFs from IP-Ribo-seq and Bulk-Ribo-seq, respectively. However, IP-Ribo-seq outperformed the Bulk approach in capturing exclusive mORFs (*n* = 16 and *n* = 6, respectively). Collectively, these data provide a comprehensive catalog of activity-dependent translational regulation of mORFs^8^. Importantly, the gene set enrichment analysis (GSEA) of IP-Ribo-seq highlighted gene ontology (GO) terms related to learning, memory, and synaptic plasticity (Fig. 1g). Together, these data demonstrated that our technique efficiently detected activity-dependent translation.

Among the significantly forward activity-dependent mORFs, we identified four previously uncharacterized candidates, *Gm13889*, *Rasl11a*, *Pramel12* and *Spty2d1* (log_2_FC > 2 and *P*_adj_ < 0.0001) (Fig. 1d, f). High-depth proteomics of hippocampal slice cultures undergoing cLTP further confirmed the protein-level expression for three of these four candidates, *Gm13889*, *Rasl11a*, and *Spty2d1*, consistent with our IP-Ribo-seq findings (Supplementary Data 1). Intriguingly, a further inspection of these previously unrecognized IEGs revealed that the N-terminal region of *Gm13889*, 1–57 aa, remains untranslated during neuronal activity (Supplementary Fig. 6a). Thus, we performed alternative ORF detection analysis using RibORF^30^ to predict alternative translation initiation of *Gm13889*. RibORF analysis suggests the non-AUG, T58/ACG, and L84/TTG are the alternative translation initiation codons for activity-dependent expression of Gm13889, with the predicted translated probability of 0.81 and 0.84, respectively, compared to its mORF, proteoform with the probability of 0.79 (Supplementary Fig. 6b; Supplementary Data 2). We suggest the 5’end of Gm13889 exhibits highly stable secondary structure, according to minimum free energy (MFE) prediction analysis using Vienna RNA^31^ (Supplementary Fig. 6c), which might be involved in non-AUG translation of *Gm13889*. Additionally, our analysis revealed that the N-terminal region, 1–57 aa, of the rat ortholog of *Gm13889*, *RGD1564664*, remains untranslated in Ribo-seq datasets from the previous studies^8^, in primary hippocampal neurons (Supplementary Fig. 6d).

### Identification of activity-dependent alternative translation in the genome

**Fig. 2:**
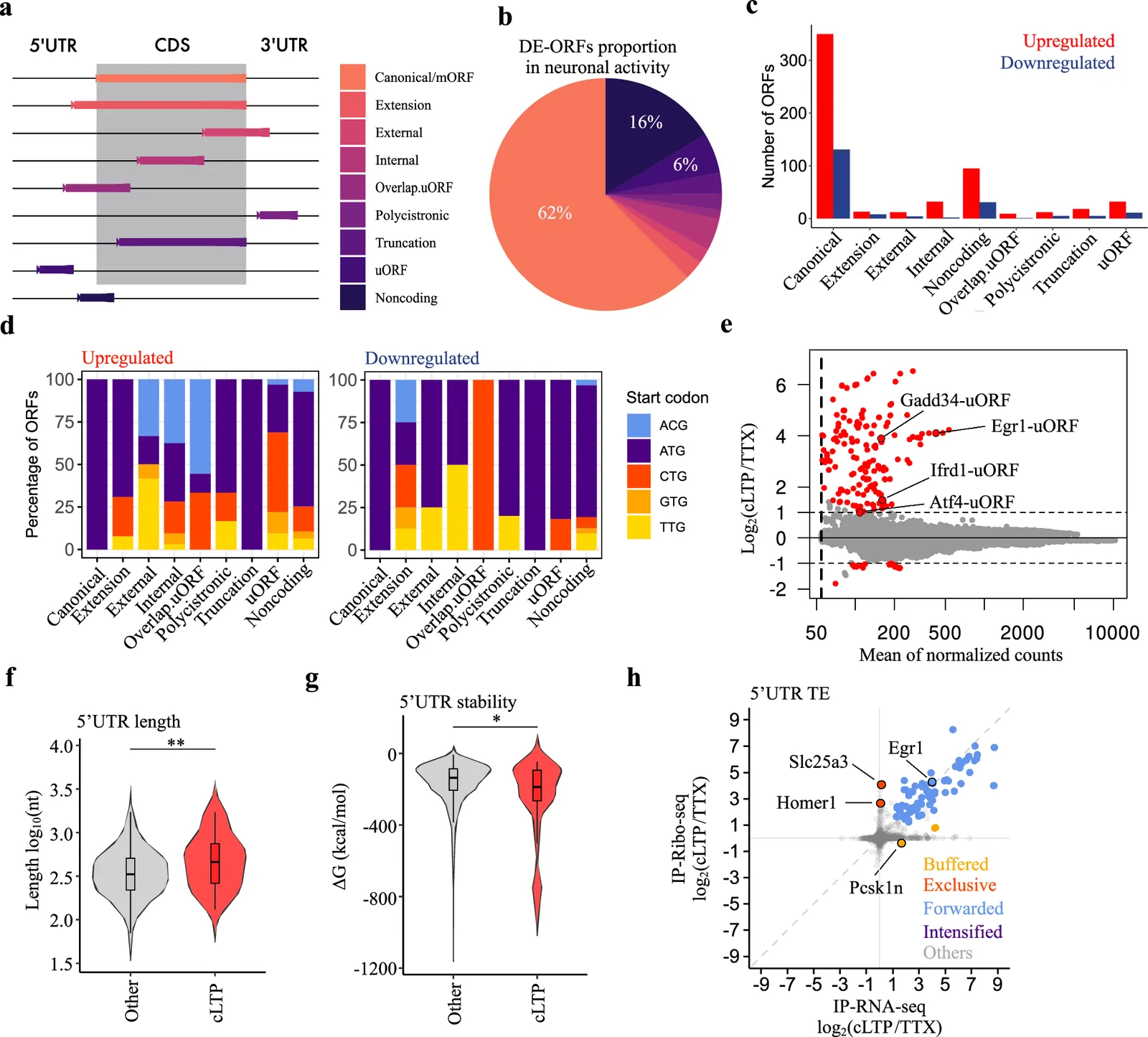
Activated-neuron-specific ribosome footprinting analysis reveals diverse types of ORF expression. **a** Schematic illustrating different types of ORFs based on their locations within mRNAs and ncRNAs. **b** Pie chart showing the proportion of differentially expressed representative ORFs upon neuronal activity, based on IP–Ribo-seq. Representative ORF selection was performed as described previously (Ji Z et al.^30^) (*n* = 3, *P* < 0.05 and |log₂FC |​​​​​ ​​> 0, two-sided Wald test with multiple-testing correction using the Benjamini–Hochberg method (FDR)). **c** Bar chart showing the total counts of differentially expressed ORFs upon neuronal activity (*n =* 3, *P* < 0.05 and |log₂FC| > 0, two-sided Wald test with multiple-testing correction using the Benjamini–Hochberg method (FDR)). **d** Stacked bar plot showing start codon usage by ORF type in upregulated (left) and downregulated (right) activity-dependent representative uORFs. **e** MA plot showing differential expression analysis of uORFs between cLTP and TTX conditions using DESeq2 with apeglm shrinkage, from three independent replicates. Red points indicate differentially regulated uORFs, highlighting previously unrecognized immediate early uORFs from *Egr1* (*P* < 0.05 and log₂FC > 1, two-sided Wald test with multiple-testing correction using the Benjamini–Hochberg method (FDR)). **f**, **g** Violin plots illustrating length dependence, **P = 0.0058 (**f**) and minimum free energy, *P = 0.026 (**g**) of the 5′ UTRs of activity-dependent uORFs. Red indicates *n*  =  44 transcripts corresponding to *n*  =  450 uORFs and other mRNAs; gray indicates *n*  =  1098 transcripts corresponding to *n*  =  61,399 uORFs. Statistical significance was assessed using a two-sided unpaired Wilcoxon rank-sum test; *P < 0.05 and **P < 0.01; data are shown as median with IQR, and the whiskers represent the minimum and maximum values. **h** 5′ UTR translational efficiency analysis using the deltaTE algorithm applied to IP-RNA**-**seq data. Blue points indicate forwarded expression (*n* = 77), orange points indicate exclusive expression (*n* = 2), and yellow points indicate buffered expression (*n* = 3); no intensified 5′ UTR-associated expression was observed (IP-Ribo-seq, *n*  =  3; IP-RNA-seq, *n* = 2).

Ribo-seq can detect less conspicuous translation signatures better than any other method and shows high performance in detecting alternative translational events across the genome^6,32^^.^ In particular, MS analysis may have limitations for analyzing short peptides such as uORF-derived peptides due to their low abundance or stability^33^. Since a comprehensive analysis of alternative translation ORF upon neural stimulation has remained elusive, we performed ORF detection analysis using RibORF^30^^.^ First, we examined RibORF performance in accurately identifying ongoing translational events, which demonstrated overall high true positive rates and low false positive rates (Supplementary Fig. 7a). As shown in Fig. 2a, RibORF classified translation into nine categories based on its position on the mRNA or genome: uORF, ncRNA, truncation, extension, external, internal, overlapping uORF, downstream ORF (dORF), also known as polycistronic, and main ORF (mORF), also known as canonical (Supplementary Data 3–10). A detailed analysis of differentially expressed ORFs upon neuronal activity indicated that ~40% of activity-dependent translation is non-canonical, suggesting that the genes other than canonical mORFs have a significant contribution to functional regulation by neuronal activity (Fig. 2b). Importantly, uORF and ncRNA accounted for 6% and 16% of total activity-dependent translation, respectively (Fig. 2b, c). To account for potential false positives where internal ORFs arise from incomplete mORF read coverage, we utilized the RibORF-based filtering strategy as detailed in Methods^30^. This approach allowed us to identify representative ORFs. Notably, ~30 uORFs and ~100 ncRNAs showed activity-dependent upregulated translation (Fig. 2c). Initiation codon usage indicated the varied relative proportion of start codon preferences across the distinct type of representative ORFs in activity-upregulated and downregulated translation, demonstrating its consistency with the general characteristics of non-canonical translation (Fig. 2d).

Emerging evidence suggests that uORFs play critical roles in regulating downstream canonical ORF^34–36^^.^ Additionally, the 5’UTR PCA analysis also indicated the stimulated and control neurons as distinct two clusters, suggesting the underlying variations in the translation are at least partly contributed by activity-dependent translation in 5’-UTR (Supplementary Fig. 7b). Thus, we aim to further characterize uORFs expressed in response to neuronal activity. As uORFs are positioned upstream and thus unaffected by potential mORF read coverage artifacts, our subsequent analysis utilized the complete set of predicted uORFs (log_2_FC > 0, *P*_adj_ < 0.05, and base-Mean>0; Supplementary Data 9; Fig. 2e). A differential expression analysis focusing on uORFs identified ~450 uORFs out of which 167 uORFs were robustly upregulated in response to neuronal activity (log_2_FC > 1, *P*_adj_ < 0.05, and baseMean >100) (Fig. 2e). Together, our Ribo-seq data revealed that the 5’-UTR showed specific translational changes in response to neuronal activity.

To explore the features of 5’-UTRs associated with activity-dependent translation, we analyzed the translation efficiency, uORF length, and MFE of 5’-UTRs (Fig. 2f, g). The length distribution revealed that activity-dependent uORFs had significantly longer 5’-UTRs compared to other mRNAs (Fig. 2f). This result suggests that longer 5’UTRs may accommodate more regulatory elements essential for activity-dependent translation. Furthermore, 5’UTRs of activity-dependent uORFs exhibited lower MFE, indicating a higher propensity for stable secondary structure formation (Fig. 2g). Moreover, we observed that the majority of activity-dependent translational changes in 5’ UTRs were correlated with corresponding transcriptional changes (~77), which is referred to as “forwarded” translational regulation, by our TE analysis from IP-Ribo-seq and IP-RNA-seq (Fig. 2h, Supplementary Fig. 7e)^26^. We also calculated TE for 5’UTR by comparing IP-Ribo-seq against conventional Bulk-RNA-seq, which identified ~88 forwarded candidates (Supplementary Fig. 7c, d). This result indicates that activity-dependent translational changes in 5’UTR are likely regulated at the transcriptional level.

To determine whether the “forwarded” translational regulation observed in the 5’-UTRs resulted from active regulation rather than a passive leaky scanning mechanism, we calculated the uORF-to-mORFs ratio as described in uORF-tools^37^. After excluding overlapping uORFs and N-terminally extended uORFs, we identified ~30 unique uORFs within the forwarded group. While four uORFs exhibited median activity scores near zero, indicative of passive leaky scanning, the majority demonstrated active regulatory potential. Specifically, we identified 17 translationally activating uORFs (mORF-up) and nine repressing uORFs (mORF-down) (Supplementary Fig. 8a). To validate these findings, we reanalyzed Bulk-Ribo-seq datasets from Cho et al.^19^, which employed a fear-conditioning paradigm to stimulate neuronal activity. This cross-study comparison confirmed a conserved set of both activating (*n* = 4) and repressing (*n* = 4) uORFs (Supplementary Fig. 8b). Together, these findings revealed distinct functional and structural properties of uORFs, which may contribute to translational regulation in response to neuronal activity.

### Activity-induced translational profiles resemble the ISR

**Fig. 3:**
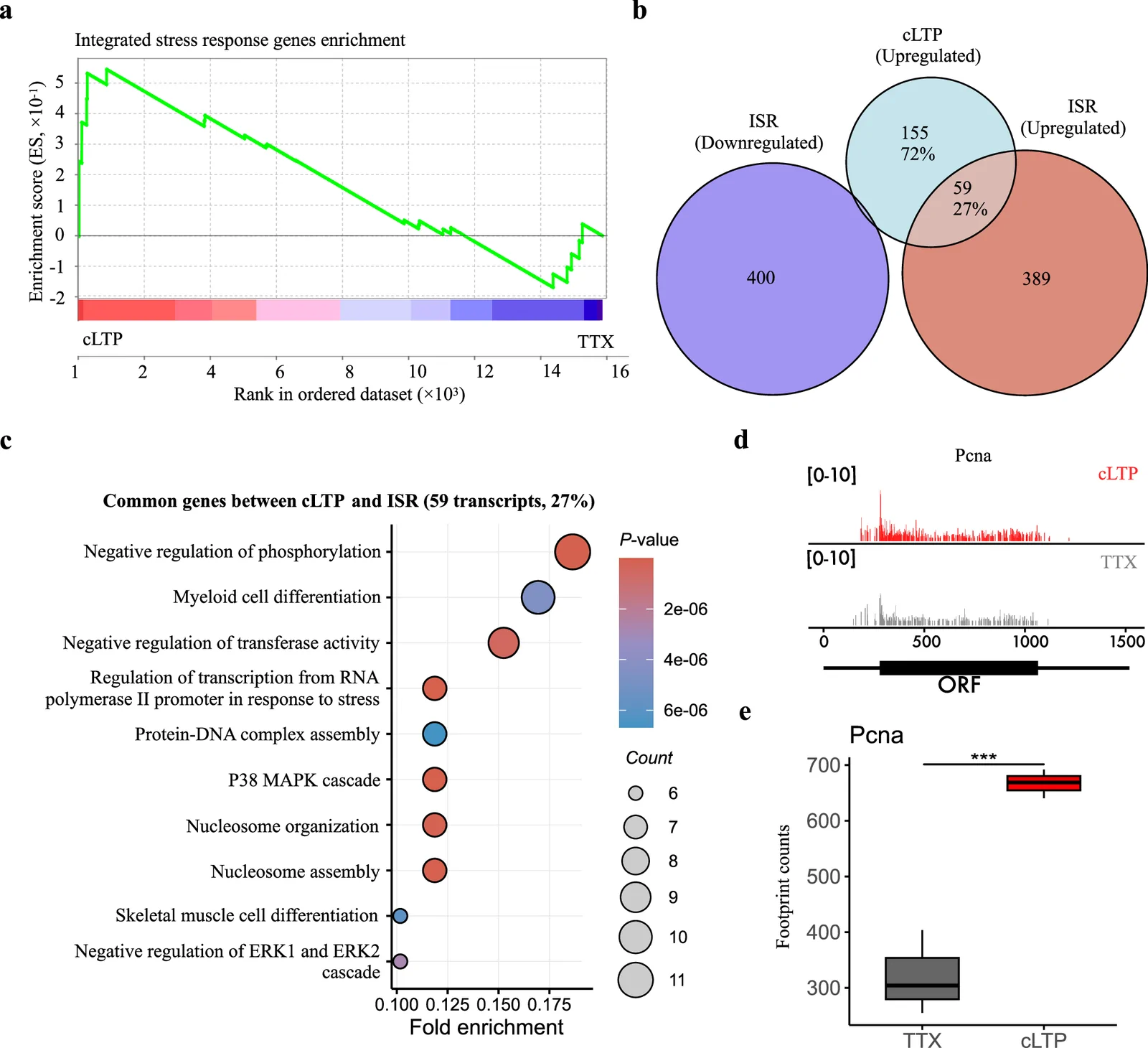
Molecular stress resembling the integrated stress response is associated with neuronal activity. **a** The enrichment of integrated stress response (ISR) signaling genes revealed by gene set enrichment analysis (GSEA) of activity-dependent Ribo-seq data based on normalized CDS expression counts from TTX-treated (*n* = 3) and cLTP-treated (*n* = 3) samples. ****P < 0.001, Kolmogorov–Smirnov-like (KS-like) permutation test. **b** Venn diagram showing that ~27% of activity-dependent genes overlap with ISR-upregulated genes. ISR-dependent genes were identified by reanalysis of ER stress (thapsigargin, THAP)-induced Ribo-seq data from Namkoong S et al.^39^. **c** ClusterProfiler gene ontology analysis of genes common to neuronal activity and ISR conditions, showing enrichment of GO terms related to regulation of phosphorylation and transcription. One-sided Fisher’s exact test with multiple-testing correction using the Benjamini–Hochberg method (FDR). **d**, **e** Differential expression of the mild ER stress-responsive gene *Pcna* in response to neuronal activity. P-site gene expression profile (**d**) and box plot showing normalized expression counts between cLTP and TTX (*n* = 3 indepe*n*dent biological replicates, ***P = 1.292314e-07, two-sided Wald test with multiple-testing correction using the Benjamini–Hochberg method (FDR)); data are shown as median with IQR, and the whiskers represent the minimum and maximum values (**e**).

To gain further insights, we investigated molecular mechanisms underlying neuronal activity by GSEA. Unexpectedly, we found a significant enrichment of ISR genes upon neuronal activity, indicating that activity-dependent translation might drive the expression of many ISR genes, including the recently reported activity-responsive gene, Gadd34^38^ (Figs. 1d and 3a). To gain further insights into the relationship between neuronal activity-dependent translation and ISR, we analyzed the previously published Ribo-Seq data from cells treated with an ER stress inducer, thapsigargin (THAP) or control^39^. Interestingly, the Venn diagram analysis showed that approximately 27% of the activity-dependent genes overlapped with the genes that are upregulated upon endoplasmic reticulum (ER) stress, one of the major mechanisms of ISR (Fig. 3b)^39–41^^.^ These results support the notion that a significant fraction of neuronal activity-induced genes were also associated with ISR. GO enrichment analysis using ClusterProfiler^42^ further displayed that the genes shared between neuronal activity and ISR are enriched for the biological processes that are related to phosphorylation regulation and transcriptional control (Fig. 3c). In addition, among these activity-dependent genes, *Pcna*, a mild ER stress-responsive gene^43^^,^ showed significant upregulation upon neuronal stimulation, representing a previously unrecognized IEG (Fig. 3d, e).

**Fig. 4:**
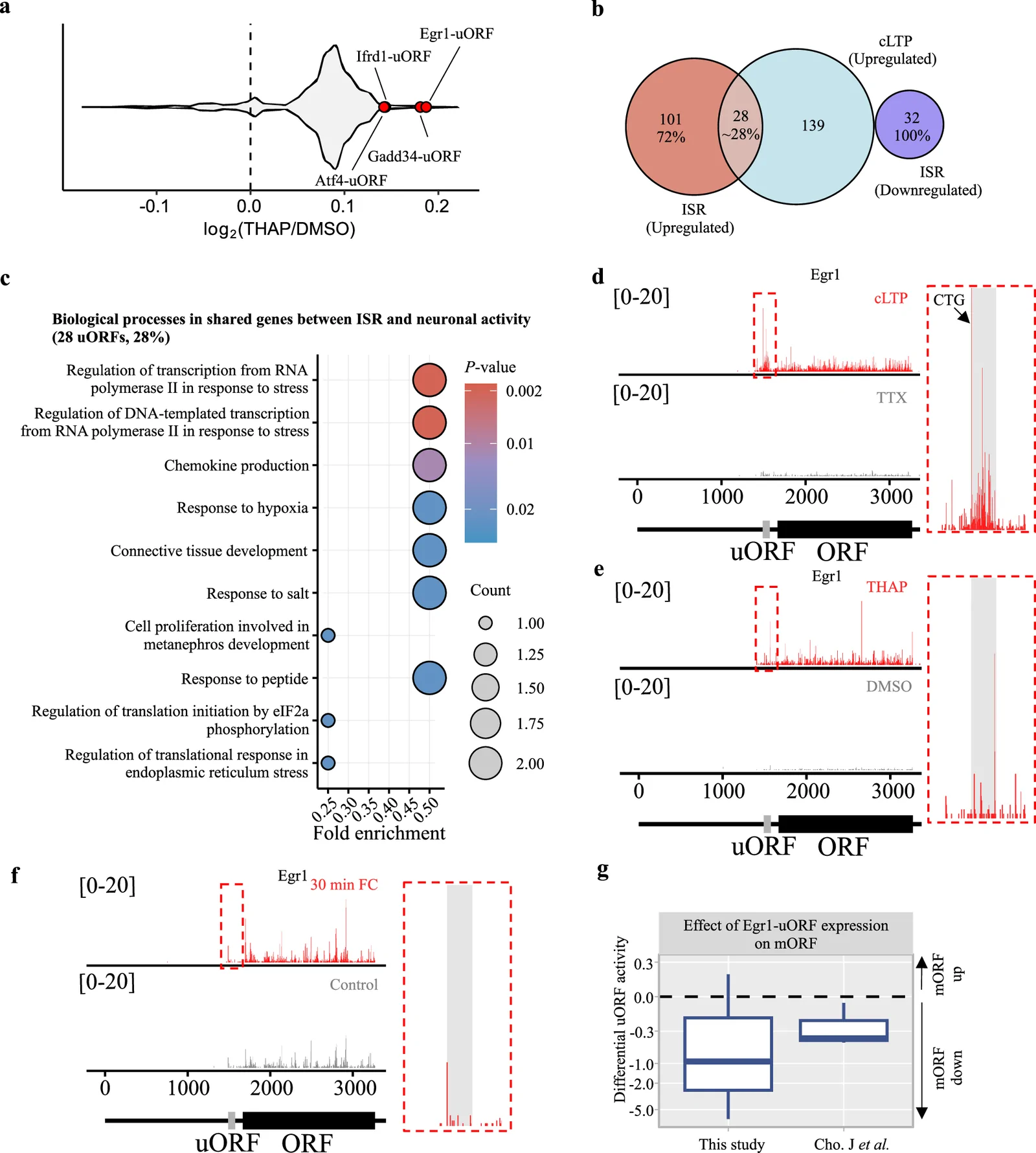
Neuronal activity implements integrated stress response-associated uORFs to prevent excitotoxicity. **a** Violin plot showing differential expression analysis of uORFs using DESeq2 with apeglm shrinkage between THAP and DMSO conditions, based on data from Namkoong S et al.^39^. Red points highlight examples of differentially upregulated uORFs, including a previously unrecognized immediate early uORF from *Egr1* (*n* = 2). **b** Venn diagram showing that approximately 28% of ISR-upregulated uORFs overlap with activity-dependent uORFs. **c** ClusterProfiler gene ontology analysis of uORFs common to neuronal activity and ISR conditions, showing enrichment of GO terms related to regulation of transcription and stress response. One-sided Fisher’s exact test with multiple-testing correction using the Benjamini–Hochberg method (FDR). **d–f** Representative P-site gene expression profiles of the immediate early gene *Egr1*, including the *Egr1*-uORF, in mouse cultured hippocampal slices under cLTP and TTX conditions (**d**), NIH3T3 cells under THAP and DMSO treatment (**e**), and mouse hippocampus in vivo 30 min after fear conditioning compared with control conditions (**f**). **g** Differential uORF activity plot for the *Egr1*-uORF in activity-dependent Ribo-seq datasets (*n* = 3) and Cho J et al.^19^. datasets (fear conditioning: 30 min compared to 5 min; *n* = 3). Negative values indicate translational repression of the mORF by the uORF, whereas positive values indicate translational amplification of the mORF by the uORF; data are shown as median with IQR, and the whiskers represent the minimum and maximum values.

Given the significant overlap between ISR and neuronal activity and the established role of uORFs in ISR, we investigated the shared and distinct regulatory features of activity- and ISR-induced uORFs. Following our analysis of activity-dependent translation within 5’-UTRs, we next characterized the structural features of 5’-UTRs associated with ISR-induced translation, such as uORF length and MFE. We found that ISR-responsive 5’ UTRs were significantly longer and exhibited lower MFE (Supplementary Fig. 9a, b). This trend is consistent with the structural features identified in 5’-UTRs regulated by neuronal activity (Fig. 2f, g). Notably, we found that 28% of ISR-dependent uORFs were also upregulated following neuronal activity, including known regulators such as *Atf4*-uORF, *Ifrd1*-uORF, and *Gadd34*-uORF (Fig. 4a, b, Supplementary Fig. 10a–g). To further explore this relationship, we performed GO enrichment analysis using ClusterProfiler. Common uORFs were enriched in biological processes related to transcriptional regulation and stress-response pathways (Fig. 4c). Collectively, these observations might imply structural and functional similarity between neuronal activity and ISR signaling and potential importance of numerous previously unrecognized uORFs that may be involved in the regulation of neuronal activity, providing a warrant for further investigation.

### Characterization of *Egr1*-uORF in neuronal activity and ISR

In IP-Ribo-seq analysis, neuronal activity increased *Egr1* expression (Fig. 4d), which is consistent with the stress-responsive role of *Egr1*^44,45^^.^ Similarly, among the previously unrecognized IEGs’ uORFs, *Egr1*-uORF also displayed a marked upregulation by an ER stressor, THAP (Fig. 4d, e). Furthermore, we analyzed the *Egr1*-uORF expression using the previous Ribo-seq datasets from Cho J. et al.^19^. Indeed, we found *Egr1*-uORF upregulation 30 min after fear conditioning (Fig. 4f). We then examined whether the ribosome footprints at *Egr1*-uORF represent an active engagement in translation or translational noise. The three-nucleotide periodicity of RPFs within the *Egr1*-uORF confirmed active translation, with ~60–90% of alignments being mapped to the first nucleotide of each codon, while a relatively smaller fraction of RPFs, ~10–40%, mapped to second and third nucleotides, indicating its robust protein expression (Supplementary Fig. 11a)^9^^.^ Additionally, we confirmed the expression of *Egr1*-uORF in rat hippocampal cultured neurons in previous Ribo-seq datasets, and found RPFs enrichment at non-AUG initiation site upon harringtonine, indicating the encoding ability of *Egr1*-uORF (Supplementary Fig. 11b)^7,8^^.^

Previous studies suggest that ER stress negatively regulates neuronal activity and prevents excitotoxicity^46,47^. Since uORFs are generally considered as a direct negative regulator of downstream canonical ORFs and *Egr1*-uORF is ER stress-responsive, we examined a potential function of *Egr1*-uORF in regulating its downstream mORF upon neuronal activity. Indeed, our differential uORF activity analysis^37^ revealed that *Egr1*-uORF negatively regulates downstream *Egr1* expression (Fig. 4g). Additionally, from our analysis of the in vivo datasets^19^, we observed a suppression of canonical *Egr1* expression by *Egr1*-uORF after 30 min of fear conditioning (Fig. 4g). These data suggest *Egr1*-uORF plays a regulatory role in modulating downstream *Egr1*-mORF expression. Thus, this mechanism may potentially explain the previously reported observation of the negative regulation of neuronal activity upon ER stress (Supplementary Fig. 12)^46,47^.

### The *Egr1*-uORF encodes a peroxisomal micropeptide

**Fig. 5:**
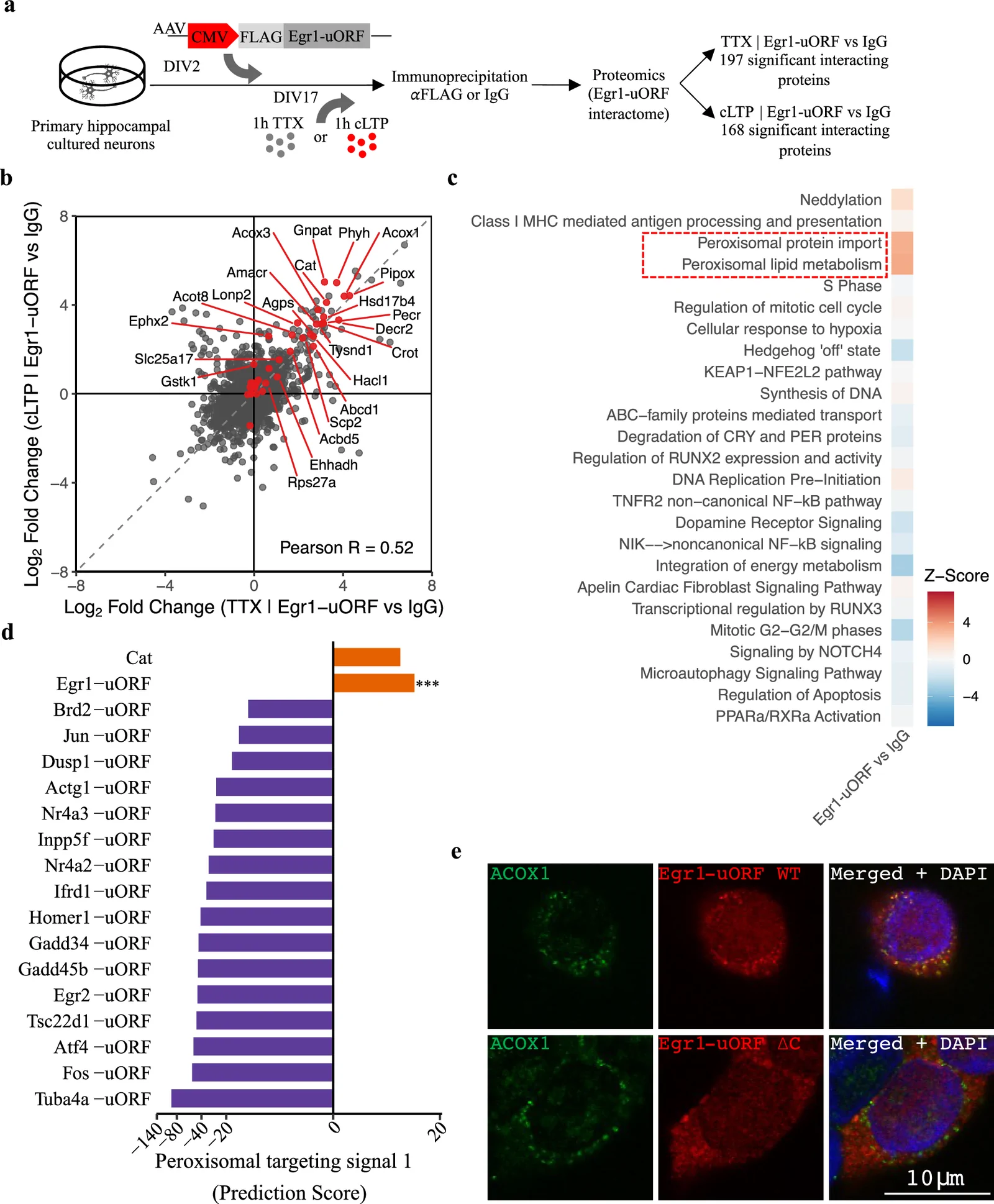
*Egr1*-uORF is a peroxisomal micropeptide that associates with import and lipid metabolism machineries. **a** Methodology of immunoprecipitation-based proteomics (IP–MS) of FLAG-tagged Egr1-uORF. FLAG-tagged Egr1-uORF was immunoprecipitated under basal (TTX) and stimulated (1 h cLTP) conditions. High-confidence interactors were identified based on reproducible enrichment across biological replicates (two-sided Welch’s *t* test, *n* = 3). **b** MS-based correlation plot showing *Egr1*-uORF interactors under 1 h cLTP and TTX conditions. Red points indicate proteins implicated in peroxisomal function (*n* = 3; log_2_FC > 0.3). **c** Comparative heatmap from ingenuity pathway analysis showing that pathways associated with *Egr1*-uORF interactors are enriched for peroxisomal protein import and lipid metabolism. **d** Peroxisomal targeting signal (PTS1) prediction demonstrating the propensity of representative activity-dependent upregulated uORFs. Only the *Egr1*-uORF exhibits a peroxisomal localization signal, ***P = 5.0e-04, calculated using empirical false positive probability as described in Neuberger G et al.^48,49^. **e** Immunofluorescence analysis of HEK cells overexpressing FLAG-tagged *Egr1*-uORF wild-type (WT) or the Δ3AA mutant, co-stained with the peroxisomal marker Acox1, demonstrates partial but greater peroxisomal colocalization of the WT compared with the Δ3AA mutant (*n* = 1).

To determine the cellular role of the *Egr1*-uORF, we first mapped its cellular interaction network in mouse primary neurons. Using immunoprecipitation-based proteomics (IP-MS) of FLAG-tagged *Egr1*-uORF, we identified a robust set of high-confidence interactors consistently maintained across both TTX and stimulated (1 h-cLTP) conditions (Fig. 5a; Supplementary Data 11). Interestingly, this interactome was highly enriched for peroxisomal components; of the 38 peroxisome-associated proteins identified in our screen, 24 exhibited a robust and reproducible association with the *Egr1*-uORF peptide (log_2_FC > 0.3; Fig. 5b). Furthermore, ingenuity pathway analysis (IPA) revealed that these interactors are involved in peroxisomal protein import and lipid metabolism pathways (Fig. 5c). Since these data indicate that *Egr1*-uORF is involved with peroxisomal biology, we investigated its amino acid (AA) sequence features for its association with peroxisomes. Indeed, the computational analysis using a PTS1 predictor^48,49^ revealed that the *Egr1*-uORF contains a PTS1 motif at its C-terminal, classifying it as a putative peroxisomal-localizing protein (Fig. 5d). Importantly, the C-terminal PAPRM motif exhibits high conservation across mammalian species^50^, suggesting that the *Egr1*-uORF likely serves a peroxisomal role maintained throughout evolution (Supplementary Fig. 11c).

Generally, the C-terminal tripeptide is the determinant of peroxisomal targeting^51^; therefore, to validate peroxisomal association, we additionally examined subcellular localization of FLAG-tagged Egr1-uORF WT and Δ3AA mutant by deleting the conserved C-terminal 3 AA, in HEK cells (Fig. 5e). Indeed, the FLAG-tagged Egr1-uORF WT showed a punctate structure that partially colocalized with the peroxisomal marker ACOX1, which was lost in its Δ3AA mutant. Together, these results identify the EGR1-uORF as a previously unrecognized peroxisomal micropeptide.

## Discussion

In this study, we developed a novel Ribo-seq technique, IP-Ribo-seq, to examine translation profiles selectively in activated neurons. Previously, the lack of cell-type specificity in translatome analysis, combined with the requirement for immediate capture of translating ribosomes upon neural stimulation, has posed major challenges for comprehensive profiling of activity-dependent translation (Supplementary Fig. 1)^5,19,52^. Here, by developing a ribosome tagging and purification strategy, we identified hundreds of ORFs (including *Arc*, *Fos*, and *Egr1*) as IEGs, which showed either upregulated or downregulated translation upon neuronal activation. We found an increased ribosome population in the 5’UTR in response to neuronal stimulation, unveiling high expression of 167 uORFs. Further analysis of these activity-regulated uORFs in hippocampal neurons uncovered functional and structural features, including a characteristic preference for non-AUG start codons, as well as longer length and higher stability of 5’UTR. The identification of previously unrecognized proteins in this study warrants further research for their function in ISR and neuronal activity. Together, our activity-dependent Ribo-seq technique not only expands the catalog of activity-dependent alternative translations but also provides a robust basis for future in-depth structural and functional analyses of activity-responsive peptides.

This study demonstrates that activity-dependent translation encompasses not only canonical mORFs but also uORFs. Among these, *Egr1*-uORF represents a non-canonical ORF within the well-known IEG *Egr1*, which encodes a transcription factor involved in modulating neuronal responses^53^^.^ Our data suggest that the translated peptide from the *Egr1*-uORF interacts with the peroxisomal machinery (Fig. 5b). Notably, the C-terminal five amino acid residues (PAPRM) of the Egr1-uORF are highly conserved across mammals and include a tripeptide peroxisome-targeting signal, potentially underlying its function (Supplementary Fig. 11c). Furthermore, we found that *Egr1*-uORF expression is upregulated not only by neural stimulation but also under ER stress conditions (Fig. 5d–f). The concurrent upregulation of *Egr1*-uORF in response to both neuronal stimulation and ISR activation raises the possibility that neuronal activity may recruit ISR-like mechanisms to attenuate ongoing translation and prevent neuronal overactivation (Supplementary Fig. 12)^46^

Our findings suggest a functional overlap between peroxisomal biology, neuronal activity, and the ISR. While peroxisomes are established as essential for memory formation^54^ and are known to trigger the ISR via import stress^55^, the role of activity-dependent translational regulation in this axis has remained elusive. By reanalysing published transcriptomic data from HEK cells undergoing peroxisomal import stress, we observed that 16% of the activity-dependent mORFs identified in our study, including the canonical IEGs *Arc* and *Fos*, are significantly upregulated at the mRNA level (Supplementary Fig. 13a, b). These overlapping candidates are primarily enriched in the MAPK signaling pathway, a key mediator of both synaptic plasticity and cellular stress (Supplementary Fig. 13c). Notably, this subset includes the human ortholog of the uncharacterized IEG *Gm13889* (*C11orf96*; Supplementary Fig. 6, Supplementary Fig. 12a), which has been independently predicted as a putative ISR candidate^56^. Collectively, these data position the Egr1-uORF, truncated *Gm13889* and related mORFs at a regulatory nexus, potentially coupling neuronal activation with peroxisomal homeostasis through the ISR.

Certain limitations of the technology and study should be considered for its application. First, the multi-step RNA selection process reduces the overall RNA yield, making in vivo library preparation challenging. Second, the time point scaling of the experiment is limited; the stability of GFP nanobody may reduce over time^20^, making it practically difficult to assess activity-dependent translation several hours after stimulation. Third, the effectiveness of GFP nanobody diffusion within activated neurons upon expression remains uncertain. Limited diffusion may enrich localization of GFP nanobody in the cell body rather than axon and dendrites, potentially reflecting soma translation while under-representing local translation. Fourth, the cross-validation of previously unrecognized activity-dependent uORFs using MS presents inherent challenges. Peptides and small proteins are generally shorter in length and may have significantly shorter half-lives than canonical proteins. Additionally, they may have lower abundance or undergo rapid degradation by the proteasome. These features may limit their detection by MS. For example, in the largest available study, Deutsch E. W. et al.^57^, only 30 reliable ncORF peptides were identified out of 3.8 billion mass spectra from 95,520 experiments, suggesting that MS is inherently limited in its capacity for validation of previously unrecognized activity-dependent uORFs. Moreover, Ribo-seq-based detection is unbiased towards the stability of the translational product, unlike MS.

The activity-dependent Ribo-seq technique is readily applicable in broader areas of neuroscience. We successfully applied our method to mouse hippocampal organotypic slices, demonstrating that activity-dependent Ribo-seq can be performed in brain tissue with complex cellular composition and circuit architecture. Given the fundamental roles of neuronal activity and translation in learning, memory, and various cognitive and neuropsychiatric disorders, this approach holds significant potential for future in vivo applications. Therefore, the technical platform developed in this study will open new avenues to reveal how neuronal activity-dependent translation impacts neuronal functions in health and disease.

## Methods

### Mice

All animal procedures were approved by the Animal Experiment Committee of Niigata University. C57BL/6 N mouse pups were purchased from SLC Japan and used for the preparation of organotypic hippocampal slice cultures immediately upon arrival. Both male and female pups at postnatal day 5–6 were used. Slice cultures were randomly allocated to experimental groups.

### Organotypic hippocampal slice culture and neuronal stimulation

Organotypic hippocampal slice cultures were prepared as described previously by Stoppini et al.^58^^.^ In brief, hippocampal slices of 325 μm thickness were dissected from both male and female C57BL/6 N mice (SLC) at the postnatal day 5-6 (P5-6) using a McIlwain tissue chopper (The Mickle Laboratory Engineering) and were plated on hydrophilic PTFE membranes (Millicell, Millipore). The slices were maintained in a culture medium containing MEM (Sigma-Aldrich), 20% heat-inactivated horse serum (GIBCO), 1 mM glutamax (GIBCO), 1 mM CaCl_2_, 2 mM MgSO_4_, 12.9 mM D-Glucose, 5.2 mM NaHCO_3_, 30 mM HEPES, 0.075% ascorbic acid, and 1 µg/ml insulin at 37 °C in a 5% CO_2_ humidified atmosphere. The culture medium was exchanged with fresh medium every other day. At day in vitro 2 (DIV2), cultured slices were divided into two groups, and AAV vectors at a titer of 0.5 × 10^13^ GC were directly added onto cultures, as follows: AAV-DJ-hSyn1-EGFP-L10a and AAV-DJ-hSyn1-HA-GFP-nanobody-HALO-PEST for the control group and AAV-DJ-hSyn1-EGFP-L10a and AAV-DJ-ESARE-HA-GFP-nanobody-HALO-PEST for the stimulation group. At DIV15-16, the culture medium was replaced by media containing 1 µM TTX (Tocris and Wako). At DIV16-17, slices were either treated with cLTP cocktail or 1 µM TTX for 15 min. The cLTP cocktail containing 50 µM forskolin (Abcam), 0.1 µM rolipram (Sigma), 50 µM (+)-bicuculline (Tocris), and 100 µM 4-aminopyridine (Tocris) was prepared in artificial cerebrospinal fluid composed of 15 mM Hepes pH 7.3, 125 mM NaCl, 20 mM glucose, 5 mM KCl, and 4 mM CaCl_2_. After the stimulation, the slices were allowed to recover for 1 h in normal culture media. Slices were then briefly washed with ice-cold PBS, immediately frozen in liquid nitrogen, and stored at −80 °C until processed for Ribo-seq and RNA-seq.

### Immunohistochemistry

Hippocampal slices were fixed with 4% paraformaldehyde for 30-60 min at room temperature, followed by washing in phosphate-buffered saline (PBS, pH7.4, Nacalai Tesque). The slices were incubated with JF646-HaloTag ligand (50 nM) for 1 h at room temperature, and then washed in PBS. After permeabilization in PBS containing 0.5% Triton X-100 (PBST) and blocking in PBST containing 5% normal goat serum, HA-Tag (C29F4) rabbit mAb (Cell Signaling, 3724, 1:1000), guinea pig polyclonal antibody against NeuN (Millipore, ABN90P, 1:1000), and/or c-Fos (9F6) rabbit mAb (Cell Signaling, 2250, 1:2000) were applied overnight at room temperature. After 2 h incubation at room temperature with secondary antibodies (goat anti-rabbit Alexa Fluor plus 555 (Invitrogen, A32732, 1:2000), goat anti-guinea pig Alexa Fluor 647 (Invitrogen, A21450, 1:2000), and/or goat anti-guinea pig Alexa Fluor 488 (Invitrogen, A11073, 1:2000)), the slices were washed in PBST. The immunolabeled sections were examined with a confocal laser scanning microscope (Olympus, FV1200). Confocal images were analyzed with ImageJ software.

For immunocytochemistry, HEK cells cultured on coverslips were fixed with 4% paraformaldehyde, 4% sucrose in PBS. Cells were permeabilized and blocked with 0.5% triton X-100, 5% normal goat serum in PBS for 45 min. Cells were incubated with primary antibodies against FLAG (Sigma Aldrich, F1084, 1:350) and ACOX1 (Proteintech, Q15067, 1:100) overnight at 4 °C, followed by incubation with fluorescently labeled secondary antibodies (1:200) for 60 min at room temperature. Nuclei were stained with 4’,6-diamidino-2-phenylindole, and fluorescent images were acquired by a Leica confocal laser microscope.

### Activated neuron-specific Ribo-seq (IP-Ribo-seq)

Ribosome profiling was performed using a protocol modified from Ingolia et al.^59^. To investigate translation profiles selectively in activated excitatory neurons from the mouse hippocampus, we employed the brain slice culture model, which expresses the sequence of interest described in the method of the organotypic hippocampal slice cultures and neuronal stimulation section. Frozen hippocampal slices were treated with 400 µl of ice-cold lysis buffer: 20 mM Tris, pH 7.4, 150 mM NaCl, 10 mM MgCl_2_, 1 mM DTT, 100 µg ml^−1^ of cycloheximide (Nacalai Tesque), and 10% Triton-X 100. The lysate was sheared 10 times using a 27-gauge syringe and centrifuged at 14,000 × *g* for 10 min at 4 °C. The supernatant, for the Ribo-seq protocol, was treated with Ambion RNase I (3.34 U/µg of RNA) at 4 °C for 45~60 min^60,61^^.^ The samples were later transferred to ultracentrifuge tubes (Beckman Coulter) containing 10% to 50% sucrose density gradient and centrifuged in a SW41-TI rotor at 274,400 × *g* for 2 h at 4 °C. The sample gradients were then fractionated using the gradient station (Biocomp) to isolate monosomes. The monosome fraction was subjected to immunoprecipitation using HALO beads (Promega, G7282) for 15 h at 4 °C on a gentle rotor. Immunoprecipitate was washed in the lysis buffer indicated above with certain modifications, including 300 mM NaCl without Triton X-100, and later resuspended in 500 µl of Sepasol-RNA I Super G (Nacalai, 09379-55) and precipitated. Using the precipitated ribosome footprints, NGS sequencing library preparation was performed as described in Ingolia et al.^59^. No rRNA depletion steps were performed. The sequencing was performed using the Novaseq X plus (Macrogen).

### Activated neuron-specific low-input pair-end RNA-seq (IP-RNA-seq)

To investigate mRNA levels selectively in activated excitatory neurons from the mouse hippocampus, we employed the brain slice culture model, which expresses the sequence of interest described in the method of the organotypic hippocampal slice cultures and neuronal stimulation section. Frozen hippocampal slices were treated with 400 µl of ice-cold lysis buffer: 20 mM Tris pH 7.4, 150 mM NaCl, 10 mM MgCl_2_, 1 mM DTT, 100 µg ml^−1^ of cycloheximide, and 10% Triton-X 100. The lysate was sheared 10 times using a 27-gauge syringe and centrifuged at 14,000 × *g* for 10 min at 4 °C. The supernatant, for the IP-RNA-seq protocol, was subjected to immunoprecipitation using HALO beads for 15 h at 4 °C on a gentle rotor. Immunoprecipitate was washed in the lysis buffer indicated above with certain modifications, including 300 mM NaCl without Triton X-100, and later resuspended in 500 µl of TRIzol and precipitated^59^.

The precipitated RNA from each sample was dissolved in 20 µl H_2_O each and outsourced for library preparation and sequencing. The sequencing libraries were constructed with the SMART-Seq® mRNA Kit (SMARTer Ultra low input RNA library (PolyA_NexteraXT)), and the paired-end (2 × 150 bp) sequencing using the NovaSeq X plus (Macrogen).

### Bulk-RNA-seq and Bulk-Ribo-seq

Ribosome profiling was performed as described in the IP-Ribo-seq Methods with certain modifications, notably omitting the immunoprecipitation step. For Bulk-RNA-seq, 500 µl Sepasol-RNA I Super G was added to the supernatant and stored at −80 °C. mRNA was subsequently purified using the Takara Oligotex-dT30 kit according to the manufacturer’s instructions and fragmented using an alkaline buffer (27 mM Na_2_CO_3_, 72 mM NaHCO_3,_ and 2 mM EDTA) at 95 °C for 40 min. For Bulk-Ribo-seq, monosome fractions were precipitated using 1000 µl Sepasol-RNA I Super G. Resulting ribosome footprints and fragmented RNAs (25–34 nt) were ligated with sample-specific unique linkers and multiplexed for library preparation. No rRNA depletion steps were performed. The sequencing was performed using the Novaseq X plus (Novogene).

### Immunoprecipitation and digestion

Culture mouse primary neurons were treated with 0.1% formaldehyde for 10 min at room temperature. The reaction was quenched by adding glycine-NaOH pH7.5 to the final concentration of 100 mM, followed by washing in phosphate-buffered saline (PBS, pH7.4, Nacalai Tesque), repeated twice. Neurons were harvested and pelleted by centrifugation.

Neurons were lysed in ice-cold RIPA buffer (Fujifilm Wako) supplemented with Benzonase nuclease (1:500, v/v; Merck) and a protease inhibitor cocktail (Roche). Lysates were sonicated using a BioRuptor (SonicBio; 30 s × 5) and centrifuged at 20,000 × *g* for 15 min at 4 °C. The supernatant was incubated with M2 anti-FLAG magnetic beads for 2 h, after which the beads were washed four times with RIPA buffer. Bound proteins were digested in 50 mM ammonium bicarbonate (ABC) containing 0.02% lauryl maltose neopentyl glycol with 0.1 µg trypsin and 0.1 µg LysC, at 37 °C overnight. The beads were removed, and the resulting peptides were reduced and alkylated with 10 mM tris(2-carboxyethyl)phosphine hydrochloride (TCEP) and 40 mM 2-chloroacetamide (CAA), and then purified using an SDB-RPS StageTip^62^.

### Protein digestion of hippocampal slice culture

Protein digestion was performed following the phase-transfer surfactant (PTS)-aided digestion protocol^63,64^. Frozen hippocampal slice cultures were lysed in a PTS buffer (100 mM Tris-HCl pH 8.5, 12 mM sodium deoxycholate (Fujifilm Wako), 12 mM sodium *N*-lauroylsarcosinate (Fujifilm Wako), 10 mM TCEP, 40 mM CAA, and a protease inhibitor cocktail (Roche)), heated at 95 °C for 5 min, and then sonicated using a BioRuptor (60 sec × 10). Protein concentration was measured using a BCA assay. After fivefold dilution with 50 mM ABC, proteins were digested with trypsin and LysC (enzyme-to-protein ratio, 1:100 each) at 37 °C overnight. After surfactant removal with ethyl acetate, peptides were purified and fractionated into eight fractions using a high-pH reversed-phase fractionation kit (Thermo Fisher Scientific).

### LC/MS/MS

For IP-MS, a nanoLC–MS/MS system comprising a Vanquish Neo UHPLC pump (Thermo Fisher Scientific) and an Orbitrap Eclipse mass spectrometer (Thermo Fisher Scientific) equipped with a FAIMSpro interface was used. Mobile phases were (A) 0.1% (v/v) formic acid in water and (B) 80% (v/v) acetonitrile containing 0.1% (v/v) formic acid. Peptides were loaded onto a 12-cm column (75 µm i.d., 3 µm particles; NTCC-360/75-3-125; Nikkyo Technos) and separated at 350 nL/min using a 40-min linear gradient (5–36% B for 25 min, 36–99% B for 5 min, and 99% B for 10 min). Spectra were acquired on the Orbitrap analyzer in data-independent acquisition (DIA) mode. MS1 scans were collected over an *m/z* range of 350–1000 at a resolution of 120,000, with a maximum injection time of 45 ms and an automatic gain control (AGC) target of 300%. For MS/MS, the precursor range was set to *m/z* 500–740 with a 4 m/z isolation width, and higher-energy collisional dissociation was performed at a normalized collision energy of 27. MS/MS scans were acquired at a resolution of 15,000 with a maximum injection time of 22 ms, an AGC target of 1000%, and a first mass of m/z 120. The FAIMSpro compensation voltage was set to −45 V.

For measurements of hippocampal slice cultures, an Orbitrap Astral mass spectrometer (Thermo Fisher Scientific) was used. Peptides were loaded onto a 25-cm Aurora ULTIMATE column (AUR3-25075C18; IonOpticks, Collingwood, Australia) and separated by a linear gradient for 20 min (5–40% B over 16 min, 40–99% B over 1 min, and 99% B for 3 min) at the flow rate of 350 nL/min. MS1 scans were acquired using an Orbitrap analyzer over an *m/z* range of 380–1180 with a resolution of 240,000, maximum injection time of 5 ms, and AGC target of 500%. Subsequent MS/MS scans were performed using an Astral analyzer. 400 scans were acquired with a 2 Th isolation window. The scan range was set to *m/z* 150–2000, HCD normalized collision energy to 27, maximum injection time to 3 ms, AGC target to 500%, and loop control time to 0.6 s.

### MS data processing

MS raw files were processed using DIA-NN (v2.3.0)^65^ in library-free mode. IP-MS data were searched against the UniProtKB mouse reference proteome (November 2025), supplemented with the 3× FLAG Egr1-uORF sequence and a list of commonly observed contaminant proteins. The following parameters were used: up to two missed cleavages; precursor charge states of 2–4; a precursor m/z range of 500–740; and a fragment ion m/z range of 120–1800. Brain slice culture data were searched against the UniProtKB mouse reference proteome, supplemented with the list of commonly observed contaminant proteins. The maximum number of variable modifications was set to three. Protein N-terminal methionine excision, cysteine carbamidomethylation, methionine oxidation, and protein N-terminal acetylation were specified. Match-between-runs was enabled. Identifications were filtered at the precursor and protein-group levels using a 1% *q* value threshold based on mutated decoys.

### IP-Ribo-seq, bulk-Ribo-seq and bulk-RNA-seq data analysis

Reads were preprocessed for trimming the adapter and one excess nucleotide at the 5’-end that is derived from reverse transcription, using cutadapt^66^. rRNAs were filtered out. RPFs of size 25 nt to 34 nt were aligned to the mouse gencode mm39 transcriptome, rat RefSeq mRatBN7.2 rna_from_genomic transcriptome, depending on the sample species, using Bowtie2^67^. Transcriptome alignments were used for all subsequent analyses unless otherwise indicated. Normalized RPF counts in each gene were obtained with the DESeq2 package in R^68–70^. The codon position of the ribosome P-site of the transcriptome-mapped reads was determined as the 12–15th nucleotide from the 5’ end, depending on the length of the reads, using custom R scripts. Mapped reads ranging from 25 nt to 34 nt were used for further analysis. The read depth plots of the ribosome P-site along each transcript were generated using R software. For all subsequent analyses, genes with an average ORF read density <0.05 were excluded or assigned a default read density equal to zero. For uORF differential expression analysis, the log_2_ fold change shrinkage was applied using an R package apeglm^71^. Differential expression analysis and translation efficiency analysis were performed using DESeq2 and deltaTE, respectively^26,68^. For the differential uORF expression analysis, uORFs with mean normalized counts above 30 were used for further analysis.

### IP-RNA-seq data analysis

Activated-neurons specific low-input pair-end RNA-seq (IP-RNA-seq) data was analyzed using nf-core/rnaseq pipeline (v3.19.0) from Nextflow data analysis framework (v25.04.6) with default parameters. Reads aligned with Salmon (v1.10.0) were processed for downstream data analysis.

### Analysis of excitatory neurons-specific enrichment by activity-dependent Ribo-seq

The neuronal transcriptome gene list was obtained from Allen Brain Map (https://portal.brain-map.org/), using the single-cell RNA-seq data from glutamatergic neurons^24^.

### GO analysis

GSEA (v4.2.3) was performed using the classic GSEA method with default parameters. The normalized expression matrix of IP-Ribo-seq data was used as an input and analyzed for gene pathway enrichments as estimated by the Enrichment Score, ES^72^. Multiple hypothesis testing was performed by computing the false discovery rate (FDR) as a default setting of parameters in the GSEA software.

For the genes commonly involved in ISR or peroxisome import stress and neuronal activity, cluster profiler analysis was performed with the default parameters^42^. No ranking procedure was employed.

### Novel ORFs detection using RibORF

A novel ORF identification analysis was performed using RibORF^30^. In brief, RPFs of size 28 nt to 30 nt were aligned to the mouse mm39 or rat mRatBN7.2 genome using the STAR aligner^73^ using the parameters –outSAMattributes All –outFilterMismatchNmax 2 –alignEndsType EndToEnd –outFilterIntronMotifs RemoveNoncanonicalUnannotated –alignIntronMax 20000 –outMultimapperOrder Random –outSAMmultNmax 1. The A-site offset correction parameter for RPFs length 28, 29, and 30 was set to 14, 14, and 15, respectively. Candidate initiation codons taken into account were ATG/CTG/GTG/TTG/ACG. ORFs shorter than 30 nt in length or with fewer than 11 mapped reads were removed. The cutoff used to select predicted translated ORF was set to 0.3, and P-site alignments for ORFs with false positive rates less than 0.1 were plotted using a custom R script.

### Differential uORF activity analysis

Differential uORF activity analysis was performed as described in UORF-Tools^37^, with certain modifications. Briefly, the metric for the relative uORF activity, *U*, was calculated as –

1 $${{U}}=\frac{{n}_{{mORF}}}{{n}_{{uORF}}}$$

where *n*_*x*_ is the normalized RPF counts (RPKM) for an indicated ORF *x*. Finally, the metric for the differential uORF activity, *ΔU, was* calculated as –

2 $$\varDelta {{U}}={\log }_{2}\left(\frac{{U}_{{stimulated}}}{{U}_{{control}}}\right)$$

where *U*_*y*_ is the relative uORF activity for an indicated condition *y*.

For the uORF analysis based on forwarded 5’UTR from deltaTE: 5’UTRs were filtered to remove those associated with overlapping uORFs and N-terminally extended proteoforms. As described in Zhe Ji et al., to account for the multiple uORFs that are derived from each distinct 5’UTR among the forwarded translation, the uORFs ending with the same stop codon were consolidated into a representative uORF during comparison across replicates and conditions. If the uORF was not expressed in a particular replicate, then a pseudo-count of the lowest normalized expression in that replicate, divided by 2, was assigned to avoid division by zero errors. These candidates were evaluated for differential uORF activity using their normalized expression (RPKM) as described in uORF-tools^37^. Differential uORF activity within an empirically defined interval of ±0.3 was classified as potential leaky scanning. Conversely, uORFs with median activity scores above 0.3 or below −0.3 were classified as translationally activating (CDS/mORF-up) or repressing (CDS/mORF-down), respectively.

### uORF conservation analysis using MEGA11

Egr1-uORF peptide sequence alignment was performed using the ClustalW program using MEGA11 with default paramaters^50^.

### MFE prediction analysis of mRNAs

MFE, thermodynamic ensemble, centroid structure, and positional entropy for Gm13889 were estimated using RNAfold webserver from the ViennaRNA package (Version 2.4.14) ^31,74^. The potentials of the 5’UTR secondary structures were estimated from the MFE by the ViennaRNA package (v2.4.14).

### Analysis of translation using Ribo-seq from previously published studies

Datasets from Cho. J. et al.^19^, Tyssowski K. et al.^75^, Namkoong S. et al.^39^, Duffy E. et al.^5^, Biever A. et al.^7^, Glock et al.^8^, Simbriger K. et al.^52^, and Jinoh Kim et al.^55^, were downloaded using pysradb^76^ and analyzed for comparison and validation.

### Statistical methods

P-site RPM counts, differential expression, and corresponding statistical tests such as the Wald test, Wilcoxon’s rank sum test, ANOVA test, Welch’s t test, and binomial test were performed using the R statistical software. To adjust for multiple comparisons, we implemented the Benjamini–Hochberg corrections whenever applicable except for the data from Namkoong S. et al.^39^ and Simbriger K. et al.^52^, which rely on *P* from the Wald test due to the small sample size. A two-sided *t*-distribution significance test was used unless otherwise indicated. During the experiments and data interpretation, the author was not blinded to group allocation.

### Reporting summary

Further information on research design is available in the Nature Portfolio Reporting Summary linked to this article.

## Supplementary information

Supplementary Information

Description of Additional Supplementary Files

Supplementary Data 1

Supplementary Data 2

Supplementary Data 3

Supplementary Data 4

Supplementary Data 5

Supplementary Data 6

Supplementary Data 7

Supplementary Data 8

Supplementary Data 9

Supplementary Data 10

Supplementary Data 11

Reporting Summary

Transparent Peer Review file

## Source data

Source Data

## Data Availability

Bulk RNA-seq and IP Ribo-seq data from this study are available in the Gene Expression Omnibus (GEO) under accession GSE295656. Bulk Ribo-seq and IP RNA-seq datasets are available under accessions GSE317973 and GSE317975, respectively. MS proteomics data from this study are available in the ProteomeXchange Consortium via the jPOST^77^ partner repository with the dataset identifier: PXD074666. Datasets linked to GEO accession GSE72064, Cho. J. et al.^19^, GSE111899, Tyssowski K. et al.^75^, GSE103667, Namkoong S. et al.^39^, GSE180240, Duffy E. et al.^5^, bioproject linked to PRJNA550323, Biever A. et al.^7^, PRJNA634994, Glock et al.^8^, GSE167197, Jinoh Kim et al.^55^, and Mendeley data linked to 10.17632/8hrj49fthr.2, Simbriger K. et al.^52^, were analyzed. Source data are provided with this paper.

## Code availability

All software and code used in this study were previously published, are cited in the Methods, and are available at: https://github.com/nayanvs/Suryawanshi_et_al._2026. The version of the code has been archived on Zenodo at 10.5281/zenodo.21273040.
