# Publisher Correction: Activity-dependent ribosome profiling reveals the landscape of canonical and non-canonical translation in brain tissue

**DOI:** 10.1038/s41467-026-76963-w

**Published:** 2026-08-20

**Authors:** Nayan Suryawanshi, Hitoshi Uchida, Ryo Endo, Kai Sato, Daisuke Satoh, Kazuya Tsumagari, Koshi Imami, Takayasu Mikuni, Motomasa Tanaka

**Affiliations:** 1 Laboratory for Protein Conformation Diseases, RIKEN Center for Brain Science, Wako, Saitama, Japan; 2 Department of Biomedical Sciences and Engineering, Graduate School of Medical and Dental Sciences, Institute of Science Tokyo, Tokyo, Japan; 3 Department of Cellular Neuropathology, Brain Research Institute, Niigata University, Niigata, Japan; 4 Department of System Pathology for Neurological Disorders, Brain Research Institute, Niigata University, Niigata, Japan; 5 Proteome Homeostasis Research Unit, RIKEN Center for Integrative Medical Sciences, Yokohama, Japan

**Keywords:** Cellular neuroscience, Gene expression analysis

Correction to: *Nature Communications* 10.1038/s41467-026-74968-z, published online 23 July 2026

In this article, the text in the legend for Fig. 2 (panels ‘D’ and ‘E’) was inadvertently swapped. The Acknowledgements section was also missing from this article and should have read, ‘We thank R. Bise for his advice on data analysis, Y. Chen for developing the data preprocessing pipelines, N. Takahashi, N. Takashima, and M. Isogai for technical assistance, Luke Lavis for providing JF646-HaloTag ligand, and the members of the Tanaka laboratory for discussions and suggestions. We are grateful to the Support Unit for Bio-Material Analysis, RIKEN CBS Research Resources Division.’ The original article has been corrected.

