# Supplementary material for: Activity-dependent ribosome profiling reveals the landscape of canonical and non-canonical translation in brain tissue
Source: Nat Commun. 2026 Jul 23;17:6179. doi: 10.1038/s41467-026-74968-z (PMC13396407; doi:10.1038/s41467-026-74968-z)
Supplement: Supplementary file 1 — Supplementary Information [file 41467_2026_74968_MOESM1_ESM.pdf]

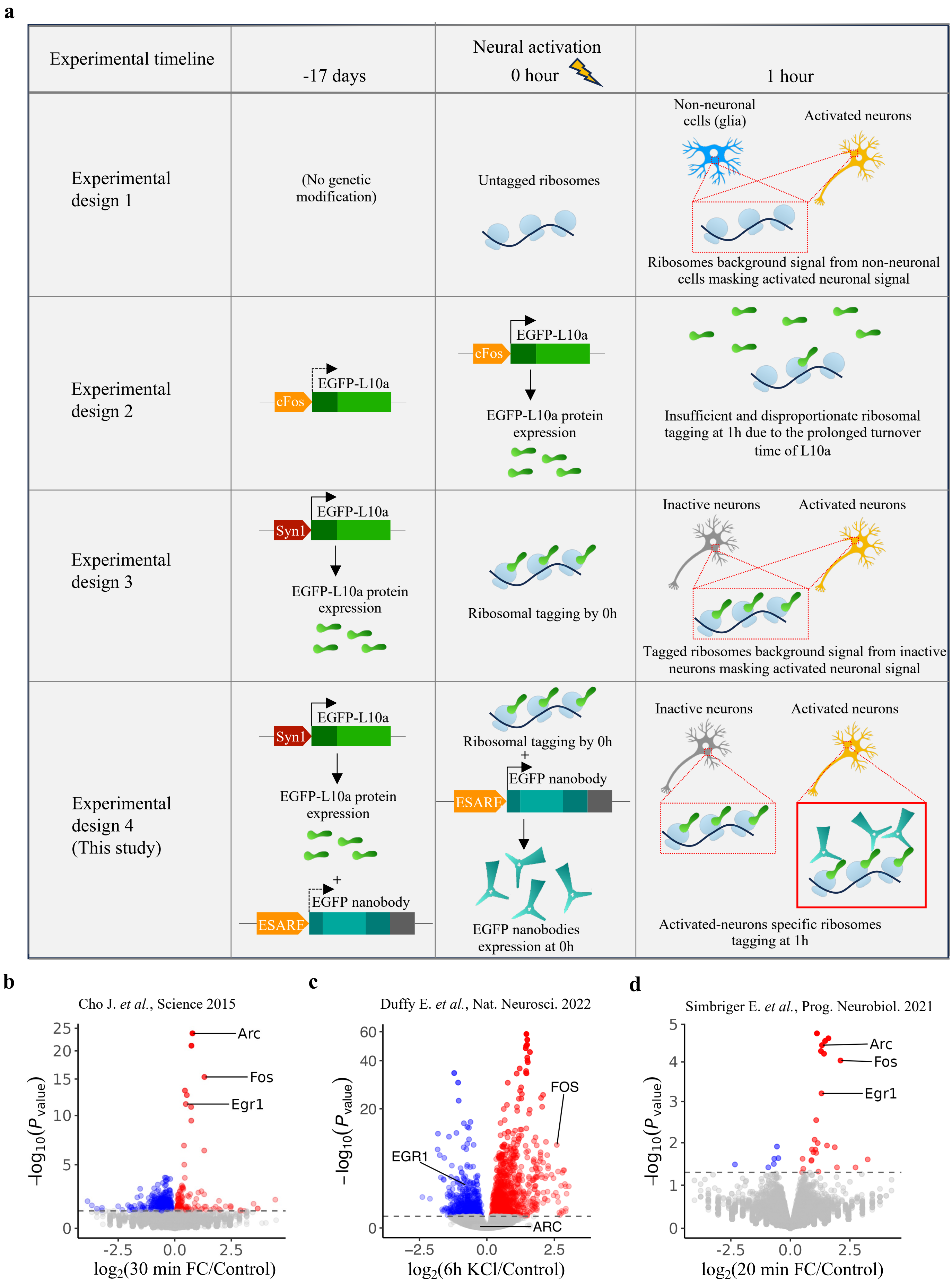
a
Experimental timeline
-17 days
Neural activation
0 hour
1 hour
Experimental design 1
(No genetic modification)
Untagged ribosomes
Non-neuronal cells (glia)
Activated neurons
Ribosomes background signal from non-neuronal cells masking activated neuronal signal
Experimental design 2
cFos
EGFP-L10a
EGFP-L10a protein expression
Insufficient and disproportionate ribosomal tagging at 1h due to the prolonged turnover time of L10a
Experimental design 3
Syn1
Ribosomal tagging by 0h
Inactive neurons
Tagged ribosomes background signal from inactive neurons masking activated neuronal signal
Experimental design 4 (This study)
ESARE
EGFP nanobody
Ribosomal tagging by 0h
EGFP nanobodies expression at 0h
Activated-neurons specific ribosomes tagging at 1h
b
Cho J. et al., Science 2015
Arc
Fos
Egr1
-log10(Pvalue)
log2(30 min FC/Control)
c
Duffy E. et al., Nat. Neurosci. 2022
FOS
EGR1
ARC
log2(6h KCl/Control)
d
Simbriger E. et al., Prog. Neurobiol. 2021
log2(20 min FC/Control)

Supplementary Fig. 1 | Differential gene expression analysis of Ribo-seq data from stimulated neurons. a, Schematics depicting a comparison of various experimental designs used to investigate activity-dependent translation in previous studies and in this study. Promoters are highlighted in red or orange, and expression sequences of the constructs are shown in shades of green. Solid and dotted arrows above the promoters indicate active and inactive protein expression, respectively. **b,** Ribo-seq analysis from mouse hippocampus 30 min after fear conditioning (FC) ($n = 3$; Cho J *et al.*[19]). Statistical significance was assessed using the Wald test followed by Benjamini–Hochberg correction. **c,** Ribo-seq analysis from hESC-derived neurons 6 h after KCl treatment ($n = 3$; Duffy E *et al.*[5]). Statistical significance was assessed using the Wald test followed by Benjamini–Hochberg correction. **d,** Ribo-seq analysis from mouse hippocampus 20 min after fear conditioning ($n = 2$; Simbriger E *et al.*[52]). Statistical significance was assessed using the Wald test. Red and blue points indicate significantly upregulated and downregulated genes, respectively (DESeq2; $P < 0.05$).

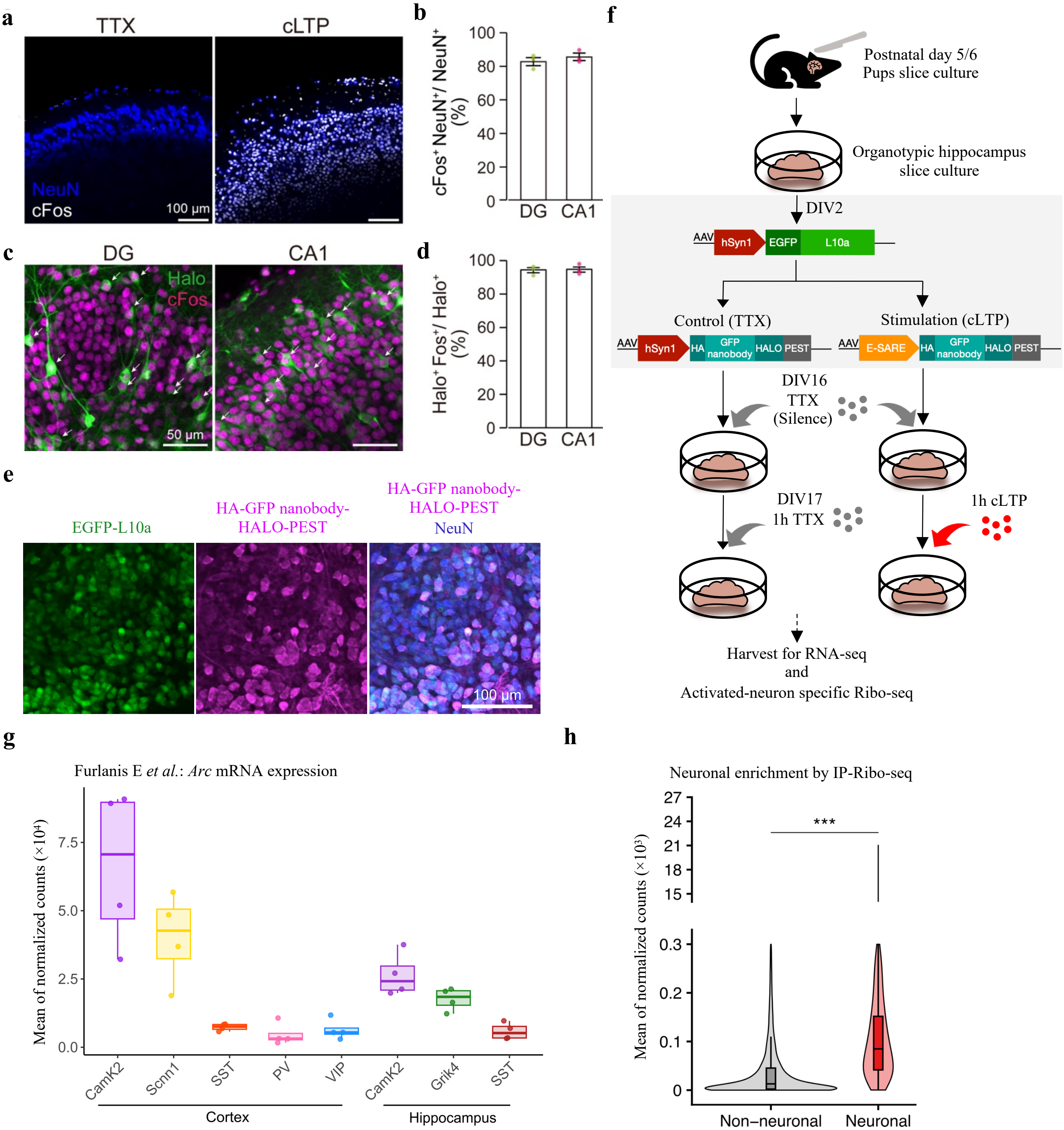

a
TTX
cLTP
NeuN
cFos
100 μm
b
cFos+ NeuN+/ NeuN+ (%)
100
80
60
40
20
0
DG
CA1
c
DG
CA1
Halo
cFos
50 μm
d
Halo+ Fos+/ Halo+ (%)
DG
CA1
e
EGFP-L10a
HA-GFP nanobody-HALO-PEST
HA-GFP nanobody-HALO-PEST
NeuN
100 μm
f
Postnatal day 5/6
Pups slice culture
Organotypic hippocampus slice culture
DIV2
AAV hSyn1 EGFP L10a
Control (TTX)
Stimulation (cLTP)
AAV hSyn1 HA GFP nanobody HALO PEST
AAV E-SARE HA GFP nanobody HALO PEST
DIV16
TTX
(Silence)
DIV17
1h TTX
1h cLTP
Harvest for RNA-seq
and
Activated-neuron specific Ribo-seq
g
Furlanis E et al.: Arc mRNA expression
Mean of normalized counts (×10⁴)
7.5
5.0
2.5
0.0
CamK2
Scnn1
SST
PV
VIP
CamK2
Grik4
SST
Cortex
Hippocampus
h
Neuronal enrichment by IP-Ribo-seq
Mean of normalized counts (×10³)
27
24
21
18
15
0.3
0.2
0.1
0
***
Non–neuronal
Neuronal

Supplementary Fig. 2 | Efficacy of activity-dependent translation methodology. a, Induction of *cFos* protein, a marker of activated neurons, in hippocampal slices 1 h after cLTP stimulation, assessed by immunohistochemistry using antibodies against NeuN (blue) and *cFos* (white). Scale bar: 100 µm. **b,** Quantification of *cFos*+/*NeuN*+ cells over total *NeuN*+ cells in dentate gyrus (DG) and CA1 regions after cLTP induction. A total of 919–1,042 or 290–642 cells in DG or CA1, respectively, from n = 3 mice were counted; data are shown as mean ± s.d. **c,** Co-localization of ESARE-driven HaloTag-GFP nanobody and *cFos* in cLTP-treated hippocampal slices, assessed by immunohistochemistry with *cFos* antibody (magenta) and Janelia Fluor dye (JF646) conjugated to HaloTag ligand (green). Arrows indicate HaloTag-GFP nanobody+/*cFos*+ cells. Scale bar: 50 µm. **d,** Quantification of HaloTag-GFP nanobody+/*cFos*+ cells over HaloTag-GFP nanobody+ cells in DG and CA1 regions. A total of 78–109 or 46–187 cells in DG or CA1, respectively, from n = 3 mice were counted; data are shown as mean ± s.d. **e,** Co-immunostaining of EGFP-L10a and HA-GFP-nanobody-HALO-PEST in hippocampal slices demonstrates activated neuronal specificity after 2 h cLTP. Green (EGFP), magenta (anti-HA), and blue (*NeuN*); n = 1. Scale bar: 100 µm. **f,** Experimental timeline: Hippocampal slices from P5–P6 mice were used for AAV-mediated gene delivery of neuron-specific GFP-tagged ribosomes and stimulation-specific Halo-tagged GFP nanobodies, along with appropriate controls. cLTP treatment was conducted on DIV17. Subsequently, excitatory neuron-specific ribosomes were immunoprecipitated, and samples were processed for Ribo-seq. **g,** RiboTRAP RNA-seq analysis of *Arc* expression in mouse brain reveals excitatory neuron specificity, as previously reported (Furlanis, Traunmüller, Fucile & Scheiffele, *Nat Neurosci*, 2019[23]). CX and HC refer to the mouse neocortex and hippocampus, respectively. Cre lines: CamK2-cre, excitatory glutamatergic pyramidal neurons, neocortical projection neurons, and hippocampal CA1 pyramidal neurons; Scnn1a-cre, layer 5 intratelencephalic (IT) excitatory neurons in neocortex; Grik4-cre, hippocampal CA3 pyramidal excitatory neurons; SST-cre, somatostatin-expressing inhibitory interneurons; Pvalb-cre, parvalbumin-expressing inhibitory interneurons; VIP-cre, vasoactive intestinal peptide-expressing inhibitory interneurons, n = 423; data are shown as mean with IQR, and the whiskers represent the minimum and maximum values. **h,** Quantification of neuronal (excitatory neurons) and non-neuronal gene expression in activity-dependent Ribo-seq analysis. A two-sided unpaired Wilcoxon test was performed on n = 4,406 neuronal transcripts and n = 36,246 non-neuronal transcripts from three independent replicates (****P* = 2.2e-16); data are shown as median with IQR, and the whiskers represent the minimum and maximum values.

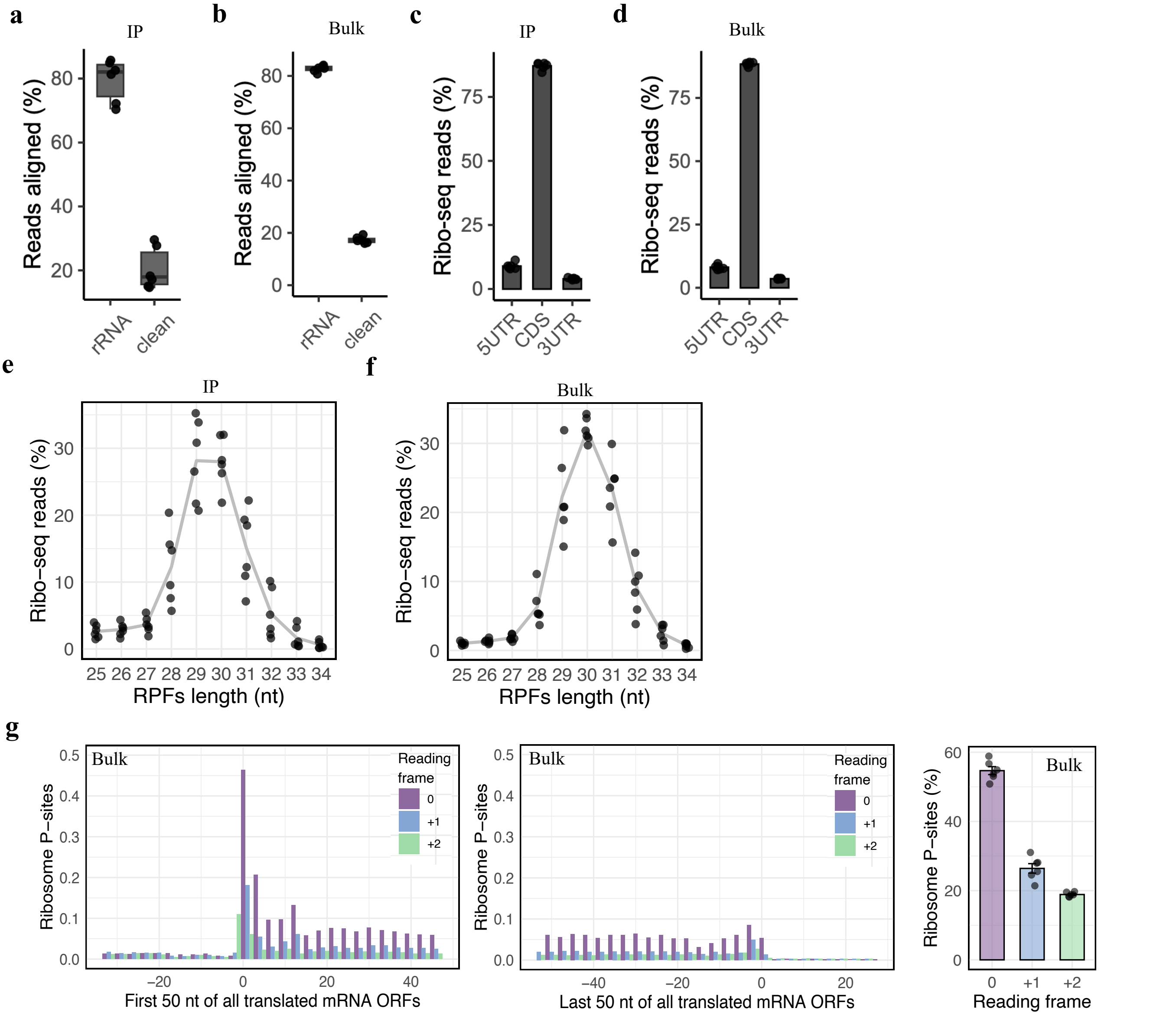

Supplementary Fig. 3 | Quality statistics of activity-dependent translation methodology. a-b, Boxplot showing the fraction of raw sequence reads derived from ribosomal RNA (*rRNA*) and remaining reads (clean) from IP-Ribo-seq (a) and Bulk-Ribo-seq (b). n = 6 per group; data are shown as median with IQR, and the whiskers represent the minimum and maximum values. **c-d,** Bar plot of the percentage of reads mapping to the coding sequence (CDS) and untranslated regions (5′ and 3′ UTRs) of mRNAs from IP-Ribo-seq (c) and Bulk-Ribo-seq (d). Data are shown as mean; n = 6 per group. **e-f,** Dot plot of sequenced ribosome footprint lengths across all six TTX- and cLTP-treated hippocampal slices from IP-Ribo-seq (e) and Bulk-Ribo-seq (f). The gray line indicates the average percentage of Ribo-seq reads assigned to a given read length across all samples. **g,** Bar plots displaying P-sites derived from Bulk-Ribo-seq reads in the first 50 nt (left) and last 50 nt (middle) of annotated ORFs, as well as the percentage of footprints in each reading frame (right); data are shown as mean ± s.e.m; n = 6.

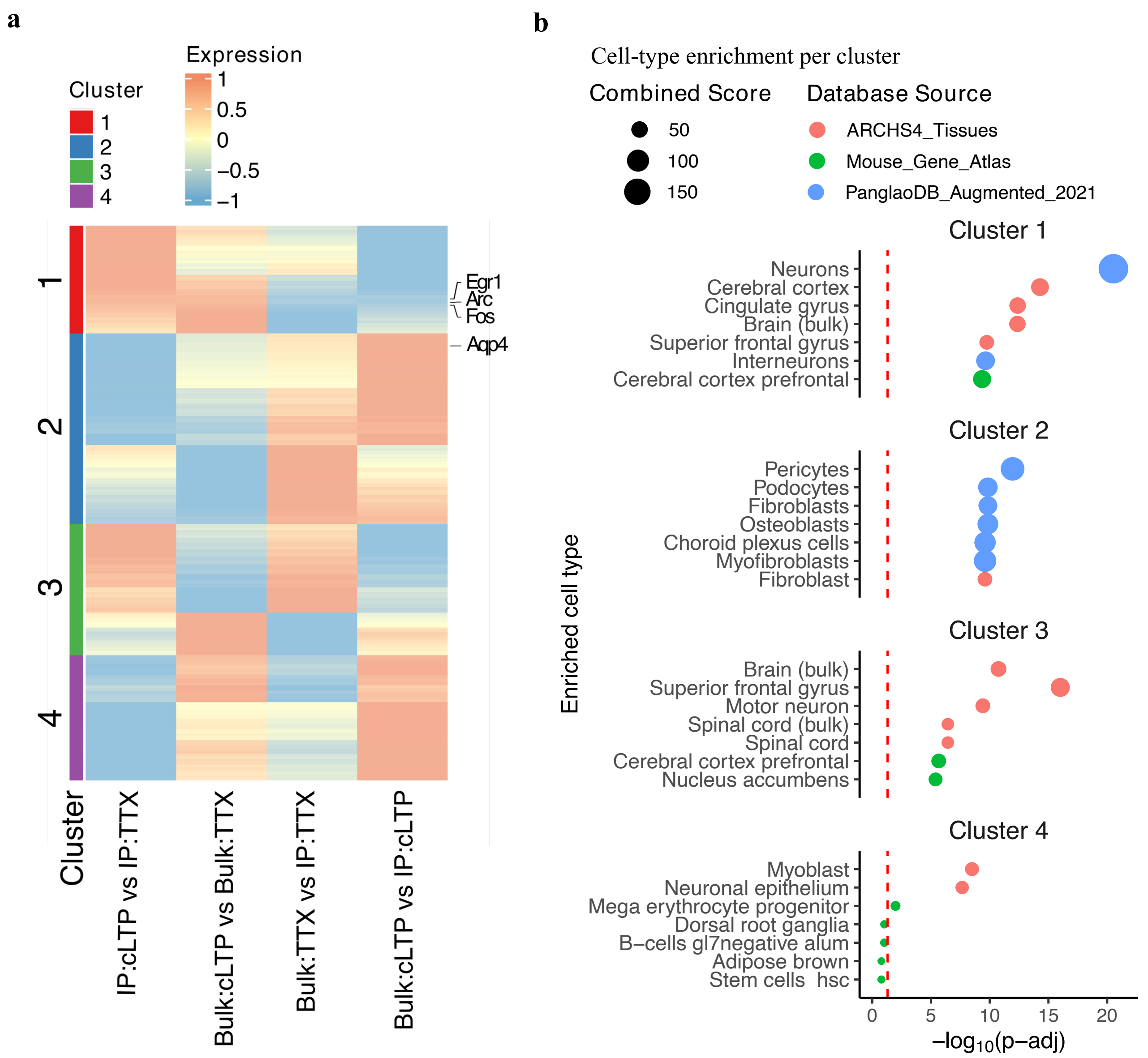

Extended Data Fig. 4 | Comparison of Bulk-Ribo-seq and IP-Ribo-seq (αHALO pulldown) in capturing activity-dependent translation of CDS/mORFs. a, Heatmap showing normalized gene expression fold changes between cLTP and TTX across Bulk-Ribo-seq and IP-Ribo-seq datasets. The color scale represents relative expression levels, and hierarchical clustering groups genes with similar expression profiles (*n* = 3). Heatmap generated using DESeq2-normalized counts and pheatmap. **b,** Enrichment dot plot of predicted cell types using Enrichr across expression clusters. For each cluster, the top enriched cell types are shown as dots. The vertical dashed red line marks FDR = 0.05. Statistical significance was determined using a one-sided Fisher's exact test followed by Benjamini–Hochberg multiple testing correction.

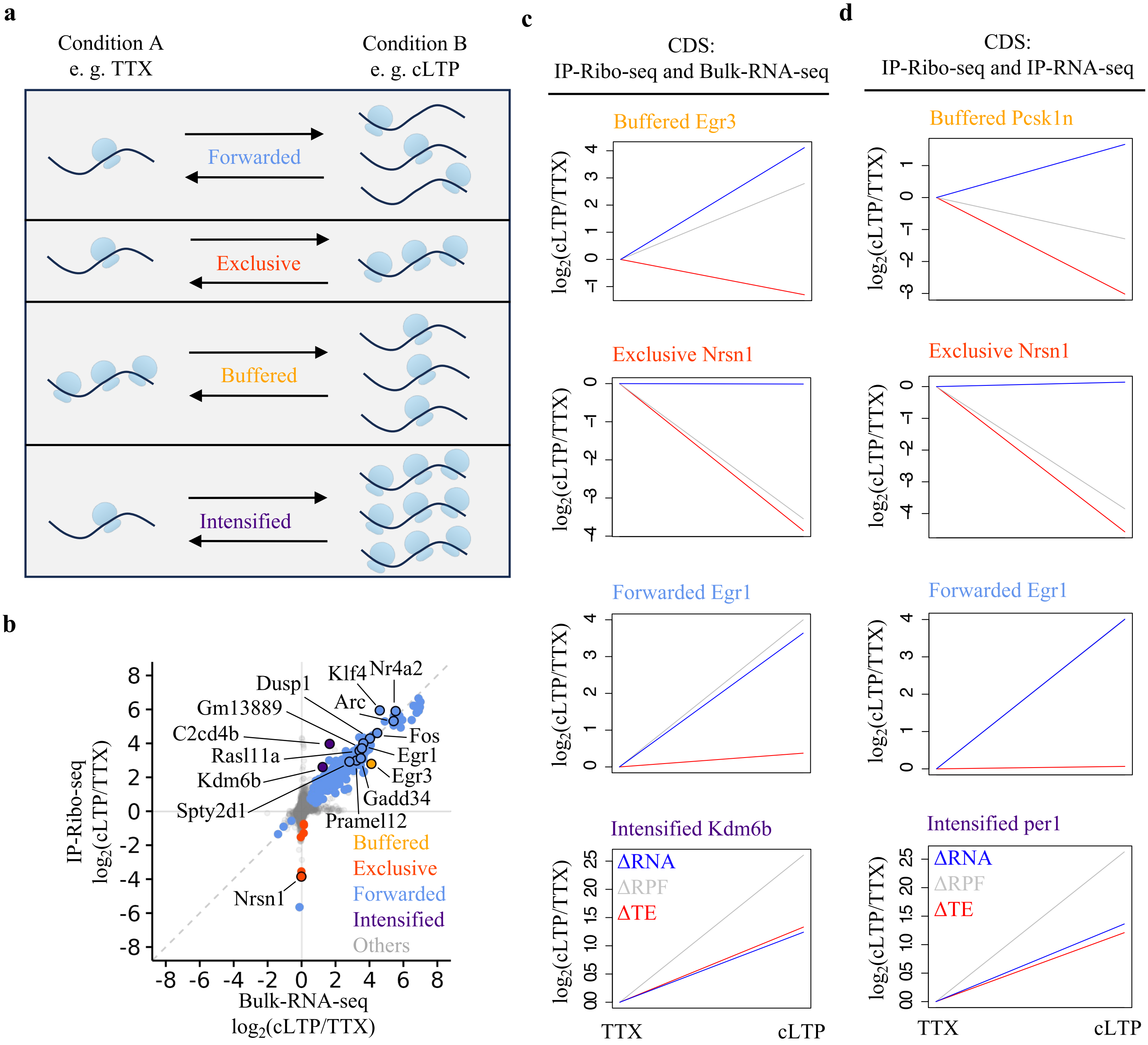

Supplementary Fig. 5 | Translation efficiency of canonical transcripts. a, Definitions of mRNA classification based on transcriptional and translational changes. Condition A and Condition B represent either TTX or cLTP. **b,** Translation efficiency scatterplots showing activity-dependent fold changes between cLTP and TTX for canonical coding sequences (CDSs; mORFs). Based on the bulk RNA-seq: blue points indicate forward expression (n = 242), magenta indicate intensified expression (n = 2), orange indicate exclusive expression (n = 6), and yellow indicate buffered expression of canonical ORFs (n = 1). Statistical significance was assessed using the Wald test followed by multiple-testing correction with the Benjamini–Hochberg method (IP-Ribo-seq, $n = 3$; Bulk-RNA-seq, $n = 3$, $P < 0.05$). **c–d,** Examples of canonical ORFs in each regulation class: translationally forwarded (c), exclusive (d), buffered (e), and intensified (f). ΔRNA, ΔRPF, and ΔTE denote differential expression at the transcriptional, translational, and translation efficiency levels, respectively. Statistical significance was determined using the Wald test followed by multiple testing correction with the Benjamini–Hochberg method ($P < 0.05$).

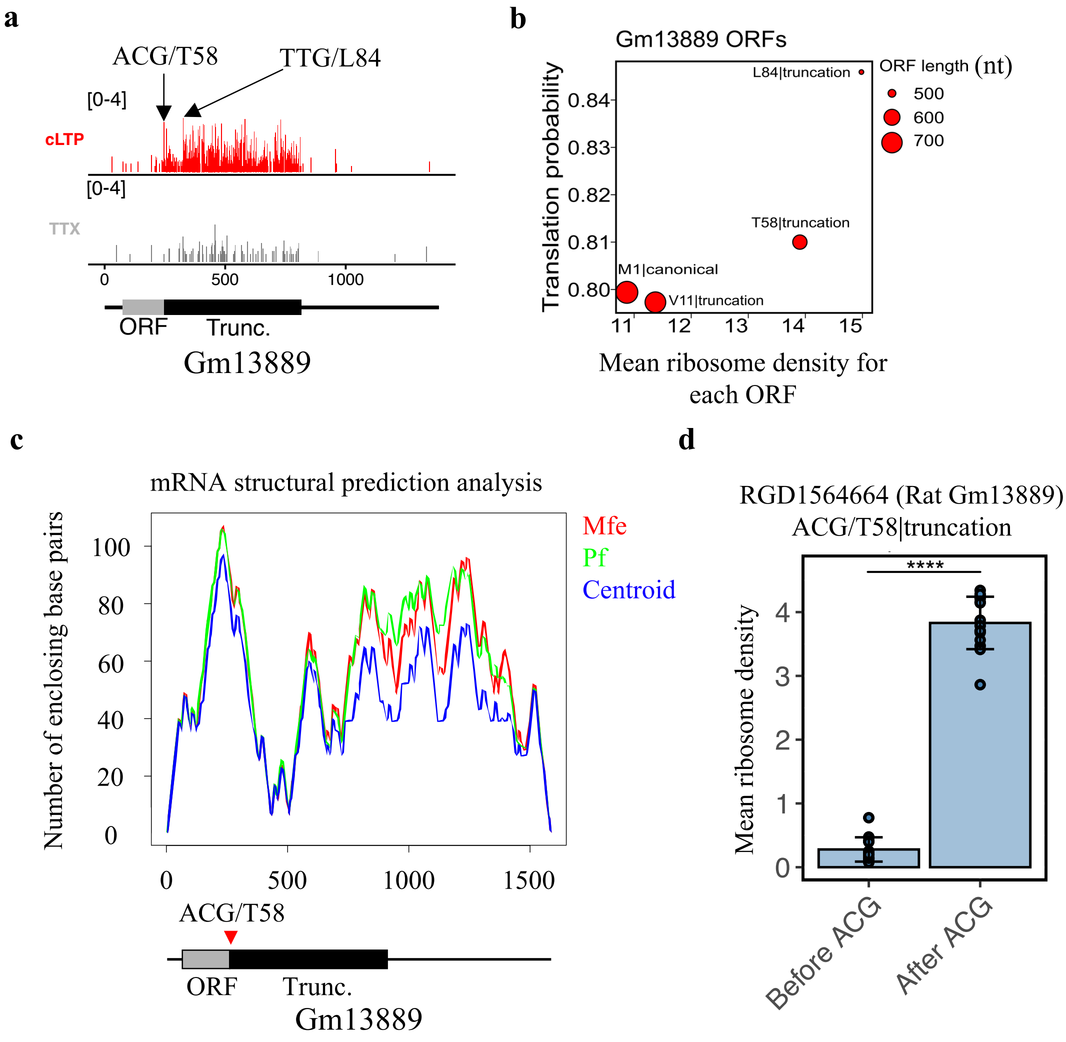

Supplementary Fig. 6 | Identification of activity-dependent N-terminally truncated expression of *Gm13889* using ribosome profiling. a, P-site gene expression profile of *Gm13889* in mouse cultured hippocampal slices under TTX (gray) and *cLTP* (red) conditions. *Gm13889* was identified as a novel truncated activity-dependent ORF with non-AUG start codons, ACG and TTG, at nucleotide positions 246 (M58) and 324 (L84) within the *Gm13889* transcript. *nt* stands for nucleotides. Gray and black box indicates the main ORF (mORF) and truncation, respectively. **b,** Median predicted translation probability of *Gm13889* proteoforms from RibORF plotted against mean ribosome occupancy as coverage per nucleotide. **c,** mRNA structural prediction using ViennaRNA revealed a highly stable structure at the N-terminal coding region of canonical *Gm13889*. MFE, PF, and Centroid denote minimum free energy structure, partition function derived from all possible structures, and structure minimizing the base-pair distance to other structures in the ensemble, respectively. **d,** Bar plot showing ribosome-protected fragment (RPF) coverage per nucleotide before and after the predicted translation initiation site of the N-terminally truncated *Gm13889* (ACG/T58) in rat hippocampal cultured neurons in the presence of harringtonine and cycloheximide, Glock *et al.*[8], Two-sided *t*-distribution test; $n = 15$, ****$P = 4.800684e-18$; data are shown as mean ± s.d.

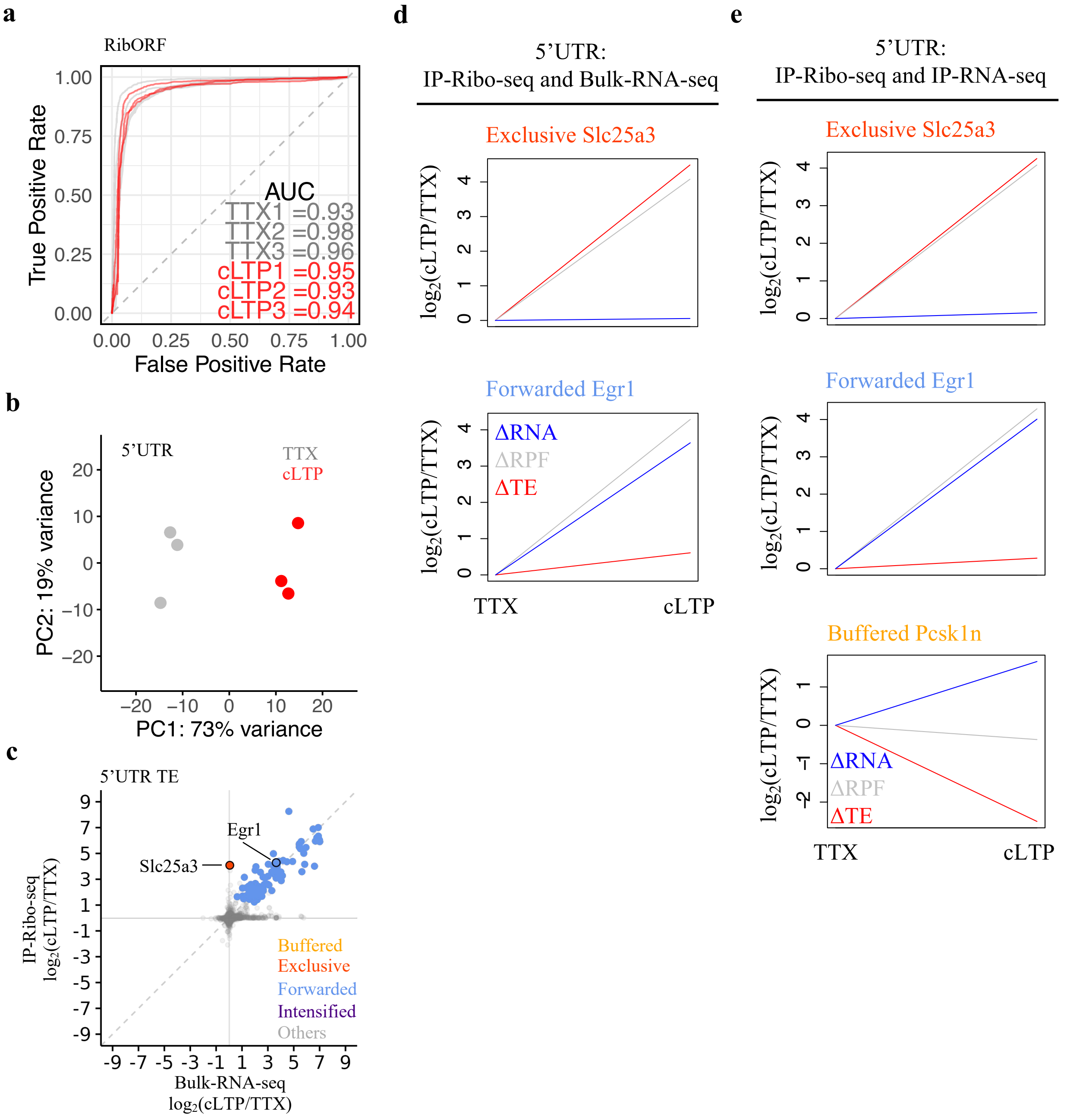

Supplementary Fig. 7 | Identification of activity-dependent uORFs. a, ROC analysis demonstrating the performance of RibORF in detecting translating open reading frames (ORFs) in slice-cultured mouse hippocampus samples (AUC ≥ 0.93). **b,** PCA plot of activity-dependent Ribo-seq expression counts for the top 500 genes in the 5′ UTR with high variance. TTX samples are shown in gray, and cLTP samples in red. **c,** 5′ UTR translational efficiency analysis using the deltaTE algorithm applied to Bulk RNA-seq data. Blue points indicate forwarded expression ($n = 88$), whereas orange points indicate exclusive 5′ UTR–associated expression ($n = 1$). No 5′ UTR–specific buffered or intensified expression was observed. Statistical significance was assessed using the Wald test followed by multiple-testing correction with the Benjamini–Hochberg method (IP-Ribo-seq, $n = 3$; Bulk-RNA-seq, $n = 3$, $P < 0.05$). **d–e,** Examples of 5′ UTR-associated expression in each regulation class: translationally forwarded (d), exclusive (e); no buffered or intensified 5′ UTR ribosome densities were observed (n = 3). ΔRNA, ΔRPF, and ΔTE denote differential expression in transcription, translation, and translation efficiency, respectively. Statistical significance was determined using the Wald test followed by Benjamini–Hochberg correction; $P < 0.05$.

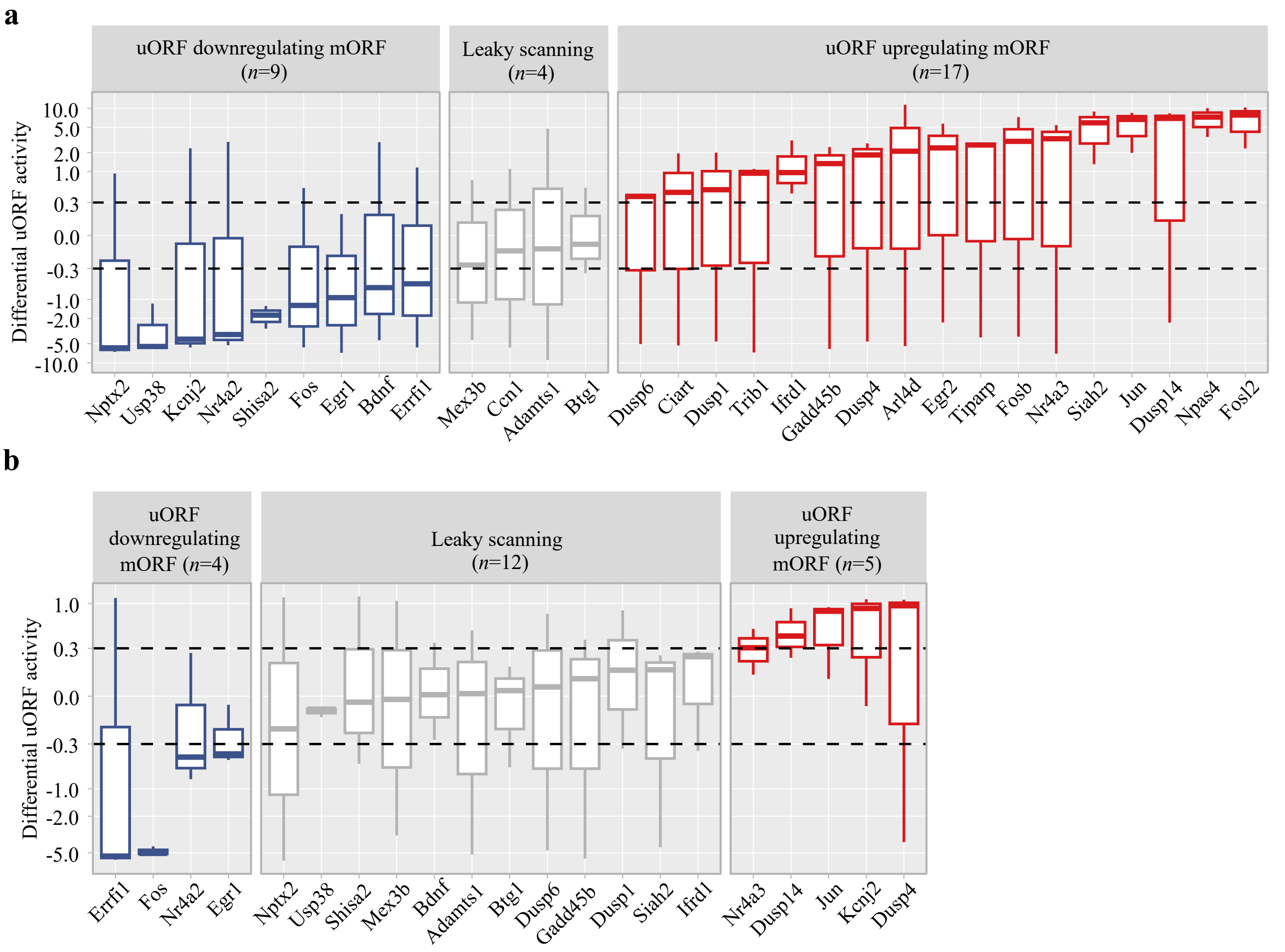

Supplementary Fig. 8 | Differential uORF activity analysis of transcriptionally and translationally forwarded 5′ UTRs in response to neuronal activity. a–b, Differential uORF activity plots for uORFs derived from forwarded 5′ UTRs (based on IP-RNA-seq) upon 1 h cLTP compared to TTX (this study, a) and in vivo fear conditioning (FC) datasets from Cho J *et al.*[19] (FC: 30 min compared to 5min) (b). Values around 0 ± 0.3 indicate predicted leaky scanning (gray), negative values (< -0.3) indicate translational repression of the main ORF (mORF) by the uORF (blue), and positive values (> 0.3) indicate translational amplification of the mORF by the uORF (red); data are shown as median with IQR, and the whiskers represent the minimum and maximum values.

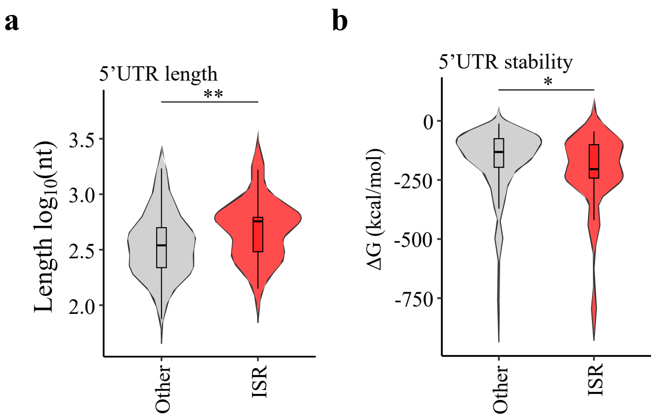

Supplementary Fig. 9 | 5'UTR length and stability of uORFs upregulated by ISR. a–b, Violin plots illustrating the length dependency (**a**) and minimum free energy (**b**) of the 5′ UTRs of ISR-dependent uORFs. ISR-dependent uORFs are shown in red (n = 30 transcripts corresponding to n = 746/53 uORFs), and other mRNAs in gray (n = 316 transcripts corresponding to n = 25,305/1,150 uORFs). Statistical significance was determined using a two-sided unpaired Wilcoxon rank-sum test; *P = 0.027 and **P = 0.005; data are shown as median with IQR, and the whiskers represent the minimum and maximum values.

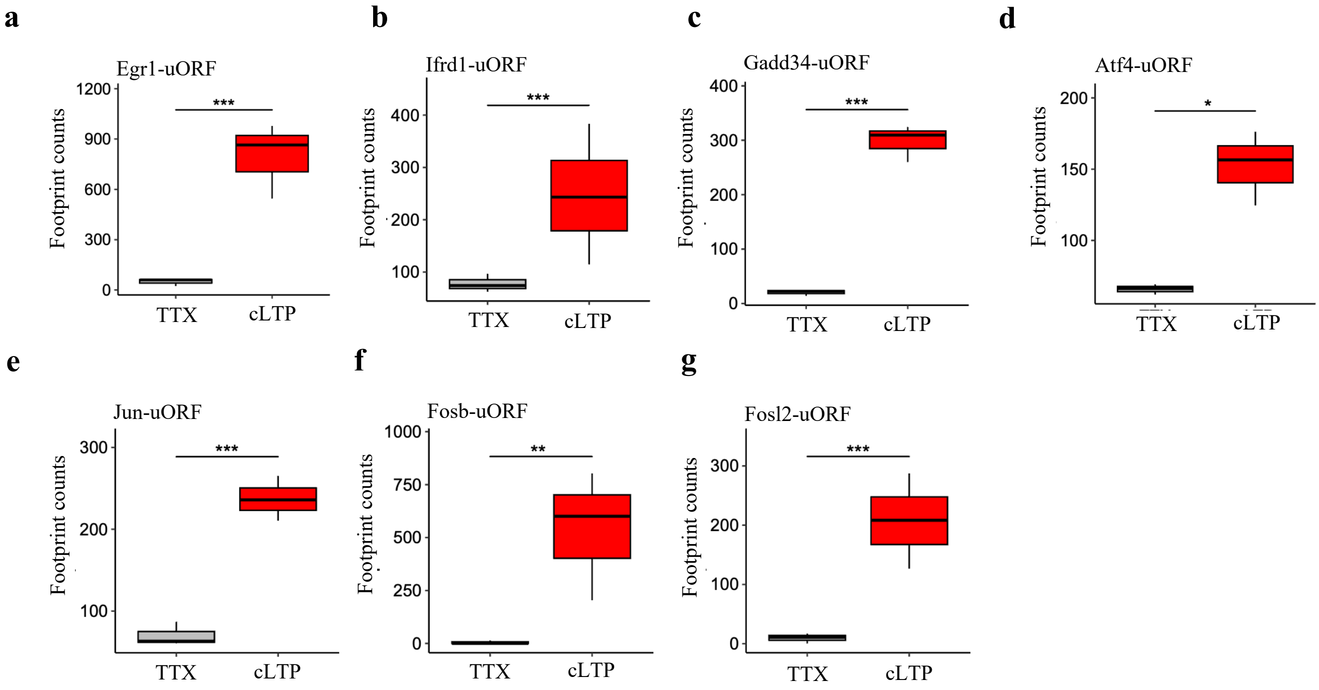

Supplementary Fig. 10 | Examples of activity-dependent uORFs associated with ISR. a–g, Examples of shared upregulated uORFs upon cLTP treatment and ISR ($n = 3$, $P < 0.05$ and $\log_2 FC > 0$, each), shown by normalized Ribo-seq expression counts. Egr1-uORF, ***P = 6.359596e-73 (a), Ifrd1-uORF , ***P = 5.033489e-05 (b), Gadd34-uORF , ***P = 1.067951e-31 (c), Atf4-uORF , *P = 0.027272855 (d), Jun-uORF , ***P = 3.876506e-07 (e), Fosb-uORF , **P = 4.038860e-03 (f), Fosl2-uORF , ***P = 1.987628e-14 (g). Statistical significance was determined using DESeq2, two-sided Wald test with multiple-testing correction using the Benjamini–Hochberg method (FDR)); data are shown as median with IQR, and the whiskers represent the minimum and maximum values.

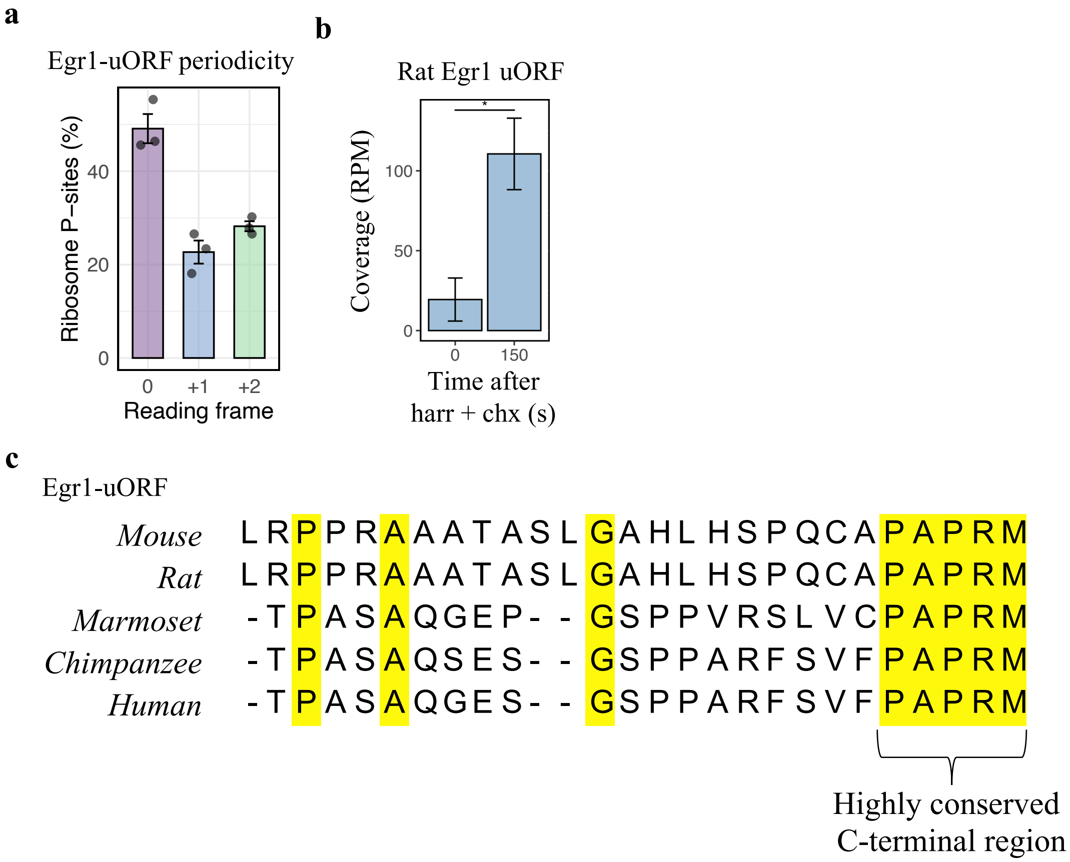

Supplementary Fig. 11 | Egr1-uORF is a novel neuronal activity- and stress-responsive uORF conserved across evolution. a, Tri-nucleotide periodicity of ribosome-protected fragments (RPFs) aligned to the *Egr1*-uORF coding region, suggesting active translation into a peptide (n = 3). Error bars represent mean ± s.d. **b,** Bar plot showing RPF coverage at the translation initiation site of the *Egr1*-uORF in rat hippocampal cultured neurons in the presence of harringtonine and cycloheximide (Glock *et al.*[8]). Statistical significance was determined using a two-sided t-distribution test; n = 3, *P = 6.9e-03; data are shown as mean ± s.d. **c,** Sequence conservation of the *Egr1*-uORF across different species, examined using MEGA11. The yellow region highlights highly conserved coding regions of the *Egr1*-uORF. Dash (–) represents sequence deletion.

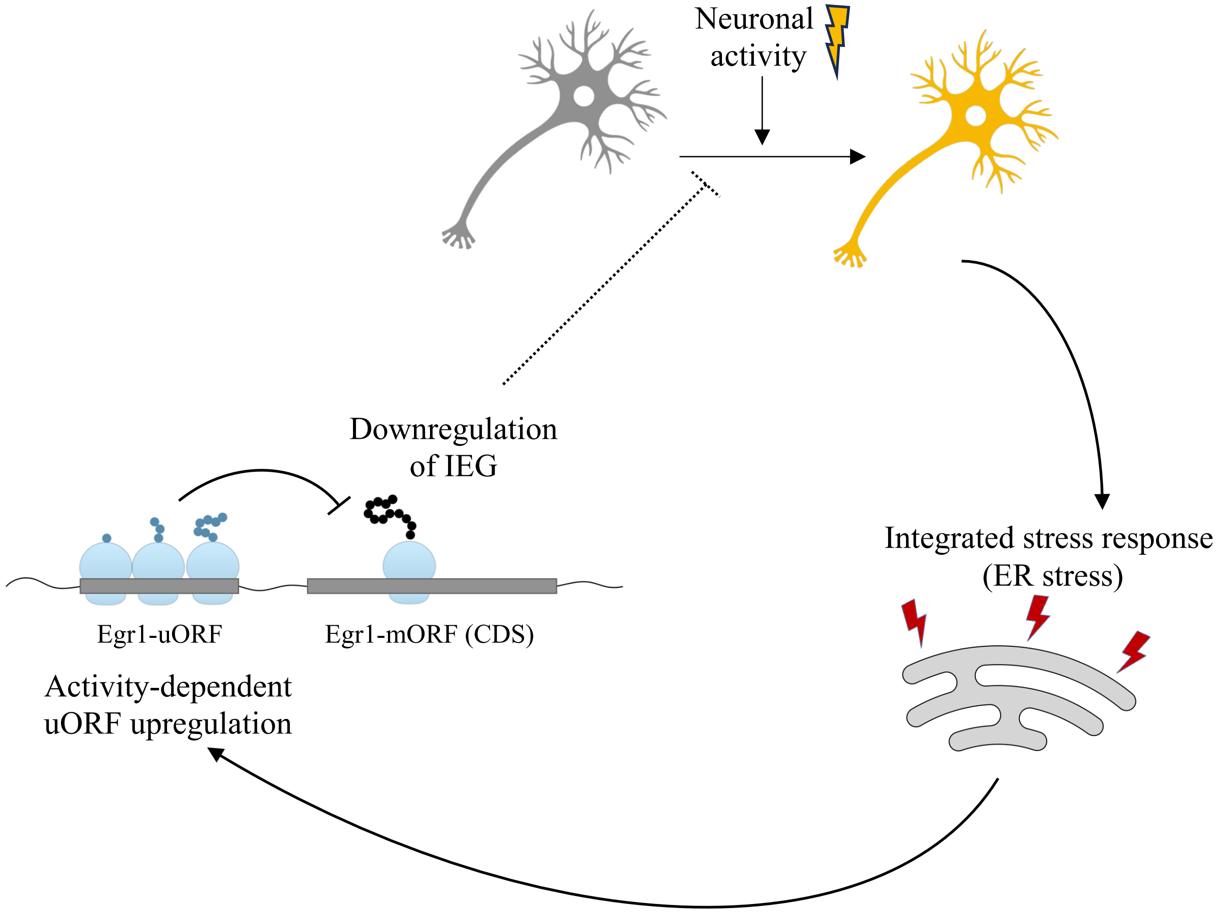

Supplementary Fig. 12 | Proposed model linking ISR and neuronal activity. Schematic model illustrating the engagement of the integrated stress response (ISR) during neuronal activity, leading to stress-responsive *Egr1*-uORF upregulation and modulation of the immediate early gene (IEG) *Egr1* expression. Solid lines indicate findings from this study, while dotted lines represent pathways or mechanisms that require further investigation.

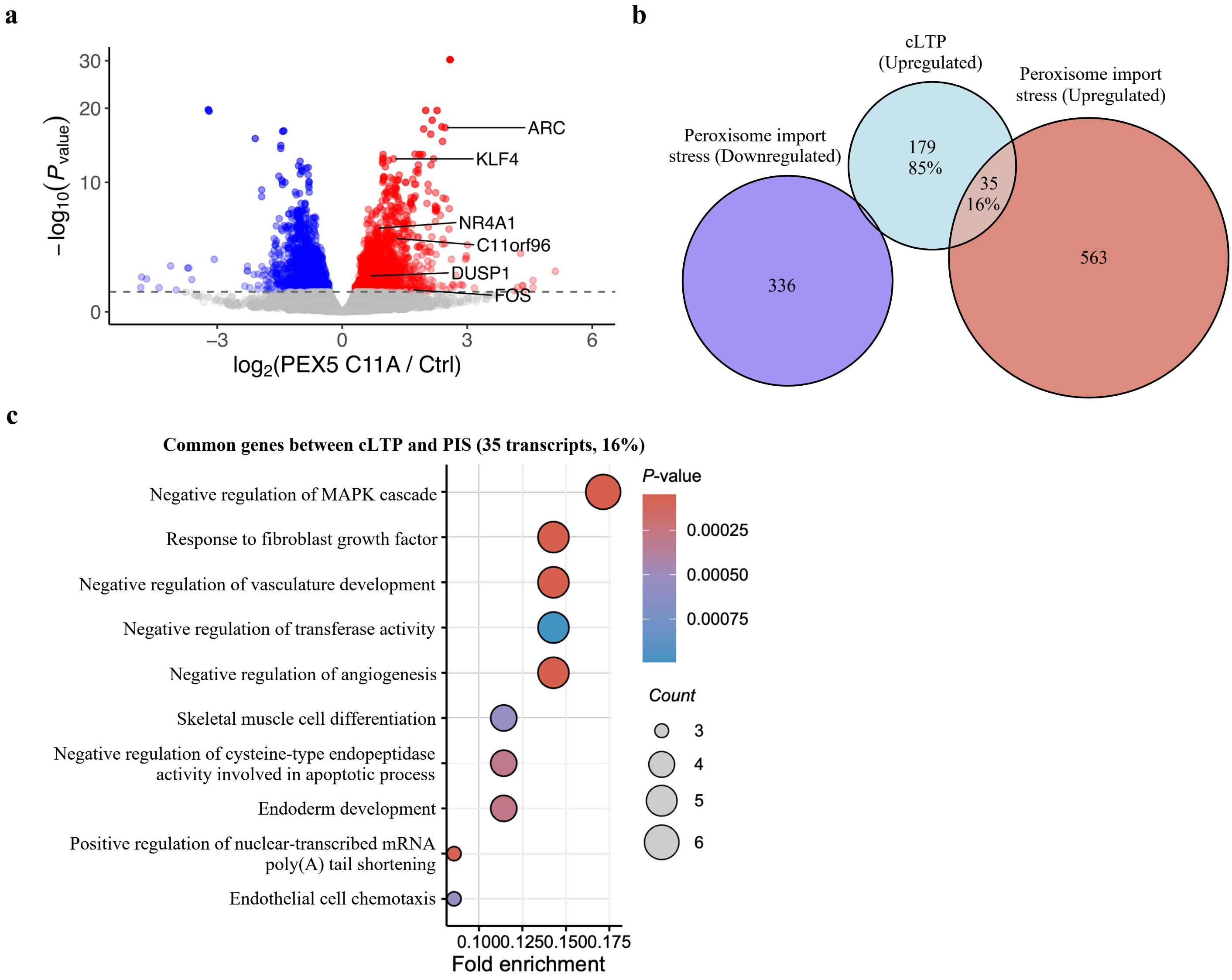

Supplementary Fig. 13 | Molecular stress resembling peroxisome import stress is associated with neuronal activity. a, Volcano plot showing RNA-seq–based differential expression analysis in HEK cells overexpressing PEX5-C11A compared with controls, from three independent replicates. Red and blue points indicate differentially upregulated and downregulated genes, respectively ($P < 0.01$, DESeq2), highlighting immediate early genes (IEGs) including *ARC*, *KLF4*, *NR4A1*, *C11orf96*, *DUSP1*, and *FOS*. Peroxisome import stress RNA-seq data were reanalyzed from Kim J *et al.*[55] Statistical significance was determined using the Wald test followed by Benjamini–Hochberg correction. **b,** Venn diagram showing that approximately 16% of activity-dependent genes are also upregulated in response to peroxisome import stress. **c,** Gene ontology analysis (ClusterProfiler) of genes common to neuronal activity and peroxisome import stress, showing enrichment for GO terms related to regulation of phosphorylation and transcription. One-sided Fisher's exact test with Benjamini–Hochberg multiple testing correction (FDR).
