# Supplementary material for: Activity-dependent ribosome profiling reveals the landscape of canonical and non-canonical translation in brain tissue
Source: Nat Commun. 2026 Jul 23;17:6179. doi: 10.1038/s41467-026-74968-z (PMC13396407; doi:10.1038/s41467-026-74968-z)
Supplement: Supplementary file 2 — Description of Additional Supplementary Files [file 41467_2026_74968_MOESM2_ESM.pdf]

Description of Additional Supplementary Files

File name: Supplementary Data 1

Description: In-depth proteomics data containing the number of prototypic peptides and fractional enrichment from organotypic hippocampal slice cultures undergoing 1 h cLTP (n = 2).

File name: Supplementary Data 2

Description: Truncated ORFs with fold changes and adjusted p-values from IP-RNA-seq data (n = 3). DESeq2; two-sided Wald test with multiple-testing correction using the Benjamini–Hochberg method (FDR).

File name: Supplementary Data 3

Description: Canonical ORFs with fold changes and adjusted *p*-values from IP-RNA-seq data (*n* = 3). DESeq2; two-sided Wald test with multiple-testing correction using the Benjamini–Hochberg method (FDR).

File name: Supplementary Data 4

Description: Extension ORFs with fold changes and adjusted *p*-values from IP-RNA-seq data (*n* = 3). DESeq2; two-sided Wald test with multiple-testing correction using the Benjamini–Hochberg method (FDR).

File name: Supplementary Data 5

Description: External ORFs with fold changes and adjusted *p*-values from IP-RNA-seq data (*n* = 3). DESeq2; two-sided Wald test with multiple-testing correction using the Benjamini–Hochberg method (FDR).

File name: Supplementary Data 6

Description: Noncoding ORFs with fold changes and adjusted *p*-values from IP-RNA-seq data (*n* = 3). DESeq2; two-sided Wald test with multiple-testing correction using the Benjamini–Hochberg method (FDR).

File name: Supplementary Data 7

Description: Overlapping upstream ORFs with fold changes and adjusted *p*-values from IP-RNA-seq data (*n* = 3). DESeq2; two-sided Wald test with multiple-testing correction using the Benjamini–Hochberg method (FDR).

File name: Supplementary Data 8

Description: Polycistronic ORFs with fold changes and adjusted *p*-values from IP-RNA-seq data (*n* = 3). DESeq2; two-sided Wald test with multiple-testing correction using the Benjamini–Hochberg method (FDR).

File name: Supplementary Data 9

Description: Upstream ORFs with fold changes and adjusted *p*-values from IP-RNA-seq data (*n* = 3). DESeq2; two-sided Wald test with multiple-testing correction using the Benjamini–Hochberg method (FDR).

File name: Supplementary Data 10

Description: Internal ORFs with fold changes and adjusted *p*-values from IP-RNA-seq data (*n* = 3). DESeq2; two-sided Wald test with multiple-testing correction using the Benjamini–Hochberg method (FDR).

File name: Supplementary Data 11

Description: Immunoprecipitation-based proteomics (IP–MS) of FLAG-tagged Egr1-uORFs.

FLAG-tagged Egr1-uORFs were immunoprecipitated under basal (TTX) and stimulated (1 h cLTP) conditions (*n* = 3, two-sided Welch's *t*-test). The data containing fold changes and adjusted *p*-values for the following comparisons are indicated:

Flagx3-Egr1_uORF_w_cLTP/Flagx3-Egr1_uORF_wo_cLTP

FLAG-tagged Egr1-uORF IP with and without cLTP

Flagx3-Egr1_uORF_w_cLTP/NIF_w_cLTP

FLAG-tagged Egr1-uORF IP versus control IP, both only under cLTP

Flagx3-Egr1_uORF_wo_cLTP/NIF_wo_cLTP

FLAG-tagged Egr1-uORF IP versus control IP, both only under TTX
