# Supplementary material for: Activity-dependent ribosome profiling reveals the landscape of canonical and non-canonical translation in brain tissue
Source: Nat Commun. 2026 Jul 23;17:6179. doi: 10.1038/s41467-026-74968-z (PMC13396407; doi:10.1038/s41467-026-74968-z)
Supplement: Supplementary file 14 — Reporting Summary [file 41467_2026_74968_MOESM14_ESM.pdf]

nature portfolio

Corresponding author(s): Takayasu Mikuni and Motomasa Tanaka

Last updated by author(s): Mar 3, 2026

Reporting Summary

Nature Portfolio wishes to improve the reproducibility of the work that we publish. This form provides structure for consistency and transparency in reporting. For further information on Nature Portfolio policies, see our Editorial Policies and the Editorial Policy Checklist.

Statistics

For all statistical analyses, confirm that the following items are present in the figure legend, table legend, main text, or Methods section.

n/a	Confirmed	
☐	☒	The exact sample size (*n*) for each experimental group/condition, given as a discrete number and unit of measurement
☐	☒	A statement on whether measurements were taken from distinct samples or whether the same sample was measured repeatedly
☐	☒	The statistical test(s) used AND whether they are one- or two-sided<br>*Only common tests should be described solely by name; describe more complex techniques in the Methods section.*
☐	☒	A description of all covariates tested
☐	☒	A description of any assumptions or corrections, such as tests of normality and adjustment for multiple comparisons
☐	☒	A full description of the statistical parameters including central tendency (e.g. means) or other basic estimates (e.g. regression coefficient) AND variation (e.g. standard deviation) or associated estimates of uncertainty (e.g. confidence intervals)
☐	☒	For null hypothesis testing, the test statistic (e.g. *F*, *t*, *r*) with confidence intervals, effect sizes, degrees of freedom and *P* value noted<br>*Give P values as exact values whenever suitable.*
☒	☐	For Bayesian analysis, information on the choice of priors and Markov chain Monte Carlo settings
☒	☐	For hierarchical and complex designs, identification of the appropriate level for tests and full reporting of outcomes
☐	☒	Estimates of effect sizes (e.g. Cohen's *d*, Pearson's *r*), indicating how they were calculated

Our web collection on statistics for biologists contains articles on many of the points above.

Software and code

Policy information about availability of computer code

Data collection	pysradb version 2.2.2
Data analysis	ImageJ Software version 2.1.<br>R version 4.3.3<br>RibORF version 1.0<br>Cutadapt version 3.5<br>Bowtie version 1.3.1<br>Bowtie2 version 2.4.5<br>STAR version 2.7.10a<br>Bedtools version 2.27.1<br>Samtools version 1.14<br>Rsamtools version 2.18.0<br>DESeq2<br>DTEG.R (https://github.com/SGDDNB/translational_regulation)<br>IPA version October 10 2025<br>Gene set enrichment analysis version 4.2.3<br>MEGA11 version 11.0.13<br>RNAfold 2.5.1<br>DIA-NN version 2.3.0<br>PTS1 predictor (https://mendel.imp.ac.at/pts1/)<br>ClusterProfiler version 4.8.3

Pheatmap version 1.0.13
Eulerr version 7.0.2
Enrichr 3.4

For manuscripts utilizing custom algorithms or software that are central to the research but not yet described in published literature, software must be made available to editors and reviewers. We strongly encourage code deposition in a community repository (e.g. GitHub). See the Nature Portfolio guidelines for submitting code & software for further information.

Data

Policy information about availability of data

All manuscripts must include a data availability statement. This statement should provide the following information, where applicable:

- Accession codes, unique identifiers, or web links for publicly available datasets
- A description of any restrictions on data availability
- For clinical datasets or third party data, please ensure that the statement adheres to our policy

Bulk RNA-seq, IP Ribo-seq, Bulk Ribo-seq, and IP RNA-seq datasets, are deposited under the SuperSeries accession GSE317977, while MS proteomics data are accessible through the ProteomeXchange Consortium via the jPOST partner repository under identifier PXD074666.

Research involving human participants, their data, or biological material

Policy information about studies with human participants or human data. See also policy information about sex, gender (identity/presentation), and sexual orientation and race, ethnicity and racism.

Reporting on sex and gender	n/a
Reporting on race, ethnicity, or other socially relevant groupings	n/a
Population characteristics	n/a
Recruitment	n/a
Ethics oversight	n/a

Note that full information on the approval of the study protocol must also be provided in the manuscript.

Field-specific reporting

Please select the one below that is the best fit for your research. If you are not sure, read the appropriate sections before making your selection.

☒ Life sciences ☐ Behavioural & social sciences ☐ Ecological, evolutionary & environmental sciences

For a reference copy of the document with all sections, see nature.com/documents/nr-reporting-summary-flat.pdf

Life sciences study design

All studies must disclose on these points even when the disclosure is negative.

Sample size	No statistical methods were used to predetermine sample size.
Data exclusions	IP-RNA-seq samples prepared using the SMARTer Ultra-Low Input RNA Library Prep Kit (PolyA_NexteraXT) were subjected to rigorous quality control; any libraries with a final concentration falling below the 10 nM threshold were excluded from subsequent Next-Generation Sequencing (NGS) due to failed QC.
Replication	RNA-seq and Ribo-seq experiments were reproduced 3 times with independent replicates. IP-RNA-seq using SMARTer Ultra-Low Input RNA Library Prep Kit (PolyA_NexteraXT) were reproduced 2 times. Mass spectrometry (MS) experiments for Egr1-uORF interactome study were reproduced at least 3 times. High depth MS were reproduced at least 2 times. Immunostaining was prepared using single replicate. Replication of studies statistically supported our conclusion in all experiments.
Randomization	n/a
Blinding	n/a

Reporting for specific materials, systems and methods

We require information from authors about some types of materials, experimental systems and methods used in many studies. Here, indicate whether each material, system or method listed is relevant to your study. If you are not sure if a list item applies to your research, read the appropriate section before selecting a response.

Materials & experimental systems

n/a	Involved in the study
☐	☒ Antibodies
☐	☒ Eukaryotic cell lines
☒	☐ Palaeontology and archaeology
☐	☒ Animals and other organisms
☒	☐ Clinical data
☒	☐ Dual use research of concern
☒	☐ Plants

Methods

n/a	Involved in the study
☒	☐ ChIP-seq
☒	☐ Flow cytometry
☒	☐ MRI-based neuroimaging

Antibodies

Antibodies used

Antibodies for immunostaining and imaging (cultured hippocampal slices):
HA-Tag (C29F4) rabbit mAb (Cell Signaling, 3724, 1:1000),
Guinea pig polyclonal antibody against NeuN (Millipore, ABN90P, 1:1000),
c-Fos (9F6) rabbit mAb (Cell Signaling, 2250, 1:2000)

Antibodies for immunostaining and imaging (HEK cells):
FLAG (M2) mouse mAb (Sigma Aldrich, F1804, 1:350),
ACOX1 (Proteintech, 10957-1-AP,1:100)

Secondary antibodies (Staining in cultured hippocampal slices):
Goat anti-rabbit Alexa Fluor plus 555 (Invitrogen, A32732, 1:2000),
Goat anti-guinea pig Alexa Fluor 647 (Invitrogen, A21450, 1:2000),
Goat anti-guinea pig Alexa Fluor 488 (Invitrogen, A11073, 1:2000)

Secondary antibodies (Staining in HEK cells)
Goat anti-rabbit Alexa Fluor plus 488 Abcam, ab150077, 1:200),
Goat anti-mouse Alexa Fluor plus 594 (Abcam, ab150116 1:200)

Validation

Antibodies for immunostaining and imaging:
HA-Tag (C29F4) rabbit mAb (Cell Signaling, 3724, 1:1000): validated by the manufacturer for the application and species. See manufacturer's website for validation statement (https://www.cellsignal.com/products/primary-antibodies/ha-tag-c29f4-rabbit-monoclonal-antibody/3724?srsltid=AfmBOoq1gBAUM4QeAER5FfFZsHmlSUh3NWlHWdpkwKpWGPtPf9L4p8a1)

Guinea pig polyclonal antibody against NeuN (Millipore, ABN90P, 1:1000): validated by the manufacturer for the application and species. See manufacturer's website for validation statement (https://www.merckmillipore.com/JP/ja/product/mm/ABN90P)

c-Fos (9F6) rabbit mAb (Cell Signaling, 2250, 1:2000): validated by the manufacturer for the application and species. See manufacturer's website for validation statement (https://www.cellsignal.com/products/primary-antibodies/c-fos-9f6-rabbit-monoclonal-antibody/2250?srsltid=AfmBOorYsW5zwqOg8benjkOxeRA2xUgD_JJo99NxQWLcZhgGlEWe7vTx)

ACOX1 (Proteintech, 10957-1-AP, 1:100): validated by the manufacturer for the application and species. See manufacturer's website for validation statement (https://www.ptglab.co.jp/products/AOX-Antibody-10957-1-AP.htm)

FLAG (M2) mouse mAb (Sigma Aldrich, F1804, 1:350): validated by the manufacturer for the application and species. See manufacturer's website for validation statement (https://www.sigmaaldrich.com/JP/ja/product/sigma/f1804?srsltid=AfmBOopyYntCkpolafGzVkadQrDGwb47c8U2uyriXlYi9GLwTJe4hDrA)

Secondary antibodies:
Goat anti-rabbit Alexa Fluor plus 555 (Invitrogen, A32732, 1:2000), validated by the manufacturer for the application and species. See manufacturer's website for validation statement (https://www.thermofisher.com/antibody/product/Goat-anti-Rabbit-IgG-H-L-Highly-Cross-Adsorbed-Secondary-Antibody-Polyclonal/A32732)

Goat anti-guinea pig Alexa Fluor 647 (Invitrogen, A21450, 1:2000): validated by the manufacturer for the application and species. See manufacturer's website for validation statement (https://www.thermofisher.com/antibody/product/Goat-anti-Guinea-Pig-IgG-H-L-Highly-Cross-Adsorbed-Secondary-Antibody-Polyclonal/A-21450)

Goat anti-guinea pig Alexa Fluor 488 (Invitrogen, A11073, 1:2000): validated by the manufacturer for the application and species. See manufacturer's website for validation statement (https://www.thermofisher.com/antibody/product/Goat-anti-Guinea-Pig-IgG-H-L-Highly-Cross-Adsorbed-Secondary-Antibody-Polyclonal/A-11073)

Goat anti-rabbit Alexa Fluor plus 488 Abcam, ab150077, 1:200): validated by the manufacturer for the application and species. See manufacturer's website for validation statement (https://www.abcam.co.jp/products/secondary-antibodies/goat-rabbit-igg-h-l-alexa-fluor-488-ab150077)

Goat anti-mouse Alexa Fluor plus 594 (Abcam, ab150116 1:200): validated by the manufacturer for the application and species. See

manufacturer's website for validation statement (https://www.abcam.co.jp/products/secondary-antibodies/goat-mouse-igg-h-l-alexa-fluor-594-ab150116)

Eukaryotic cell lines

Policy information about cell lines and Sex and Gender in Research

Cell line source(s)	LentiX HEK 293T cells were purchased from Takara (Cat. num. 632180)
Authentication	We did not independently authenticate the HEK 293T cell line. LentiX-293T cells were validated by morphology.
Mycoplasma contamination	Manufacturer validation: Each production lot is tested for sterility and Mycoplasma contamination. Lab validation: LentiX HEK 293T cells tested tested negative for Mycoplasma contamination.
Commonly misidentified lines (See ICLAC register)	n/a

Animals and other research organisms

Policy information about studies involving animals; ARRIVE guidelines recommended for reporting animal research, and Sex and Gender in Research

Laboratory animals	C57BL/6N mice (SLC)
Wild animals	n/a
Reporting on sex	Sex was not considered as a primary variable because the study aimed to characterize the fundamental molecular mechanisms of protein expression triggered by neuronal activity, processes which are fundamentally conserved across the species studied.
Field-collected samples	n/a
Ethics oversight	Mice were handled according to the protocol approved by Niigata University.

Note that full information on the approval of the study protocol must also be provided in the manuscript.

Plants

Seed stocks	n/a
Novel plant genotypes	n/a
Authentication	n/a
