# Supplementary figures and images for: Activity-dependent ribosome profiling reveals the landscape of canonical and non-canonical translation in brain tissue
Source: Nat Commun. 2026 Jul 23;17:6179. doi: 10.1038/s41467-026-74968-z (PMC13396407; doi:10.1038/s41467-026-74968-z)

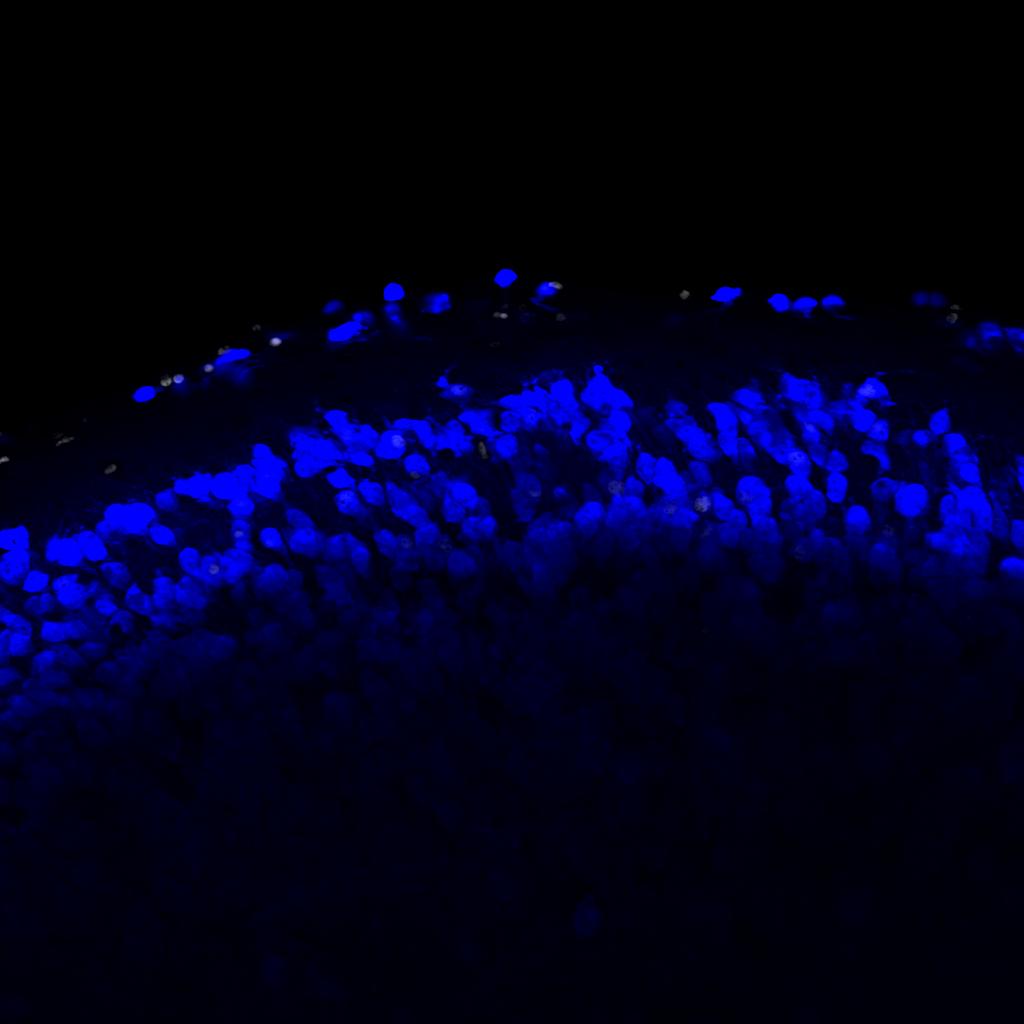

Supplement: Supplementary file 16 — Source Data [file 41467_2026_74968_MOESM16_ESM.zip › Source_data/Supplementary Fig2a_left.jpg]

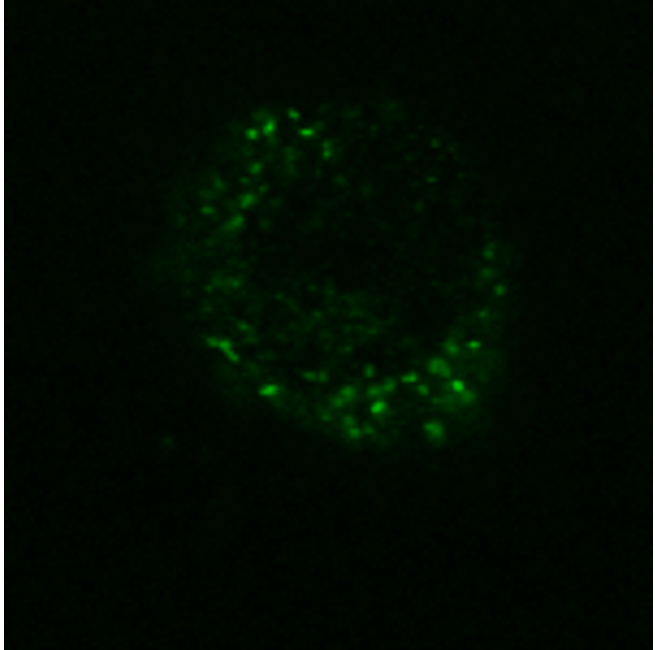

Supplement: Supplementary file 16 — Source Data [file 41467_2026_74968_MOESM16_ESM.zip › Source_data/Fig 5e_top left.png]

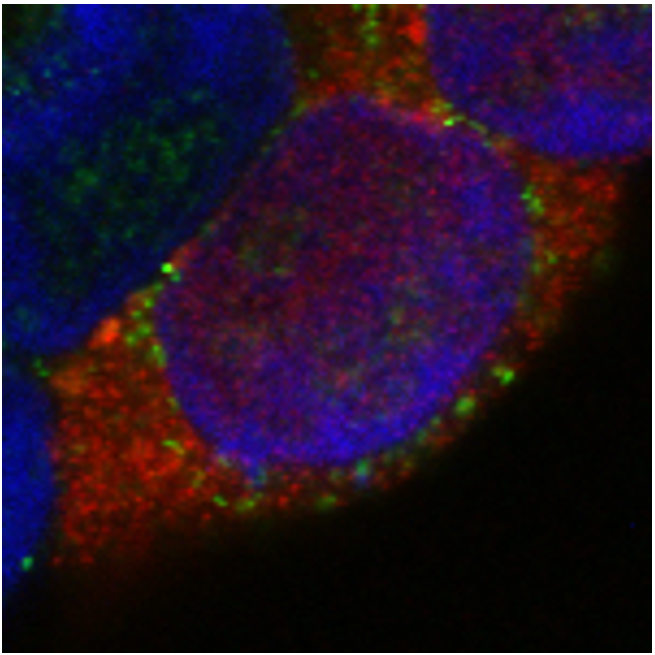

Supplement: Supplementary file 16 — Source Data [file 41467_2026_74968_MOESM16_ESM.zip › Source_data/Fig 5e_bottom right.png]

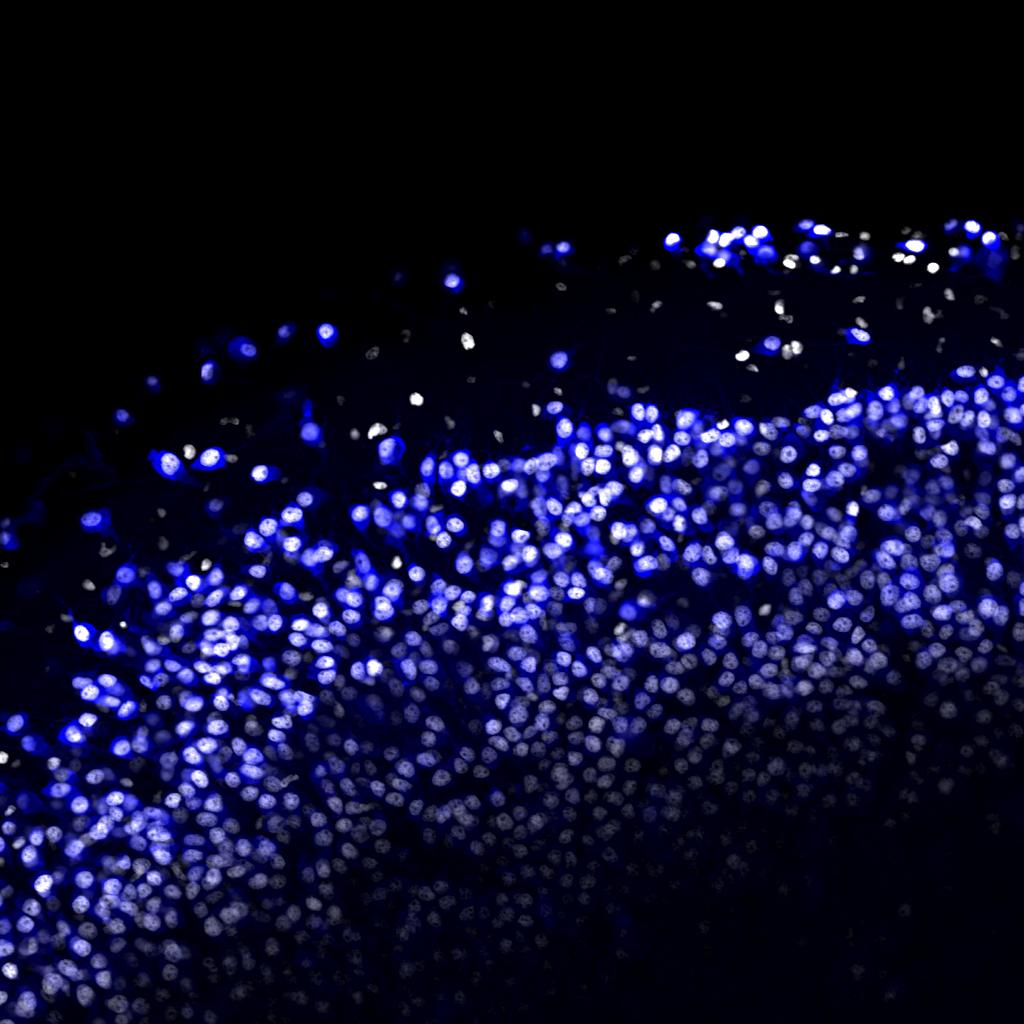

Supplement: Supplementary file 16 — Source Data [file 41467_2026_74968_MOESM16_ESM.zip › Source_data/Supplementary Fig2a_right.jpg]

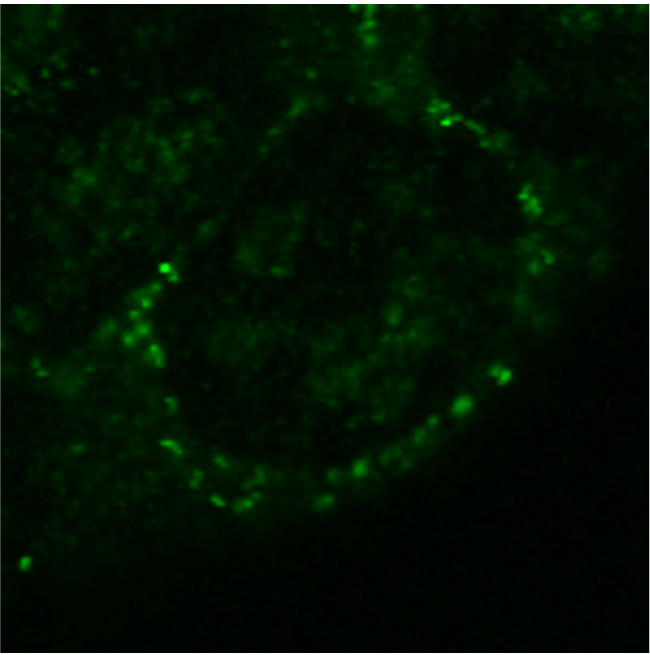

Supplement: Supplementary file 16 — Source Data [file 41467_2026_74968_MOESM16_ESM.zip › Source_data/Fig 5e_bottom left.png]

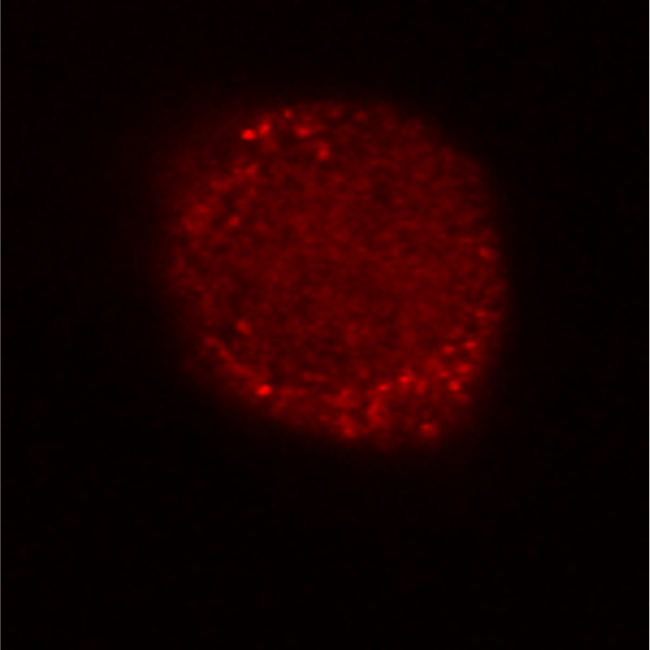

Supplement: Supplementary file 16 — Source Data [file 41467_2026_74968_MOESM16_ESM.zip › Source_data/Fig 5e_top middle.png]

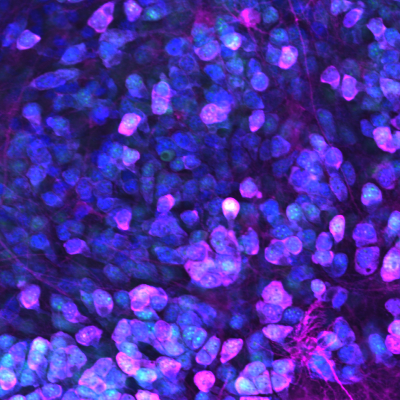

Supplement: Supplementary file 16 — Source Data [file 41467_2026_74968_MOESM16_ESM.zip › Source_data/Supplementary Fig2e_right.tif]

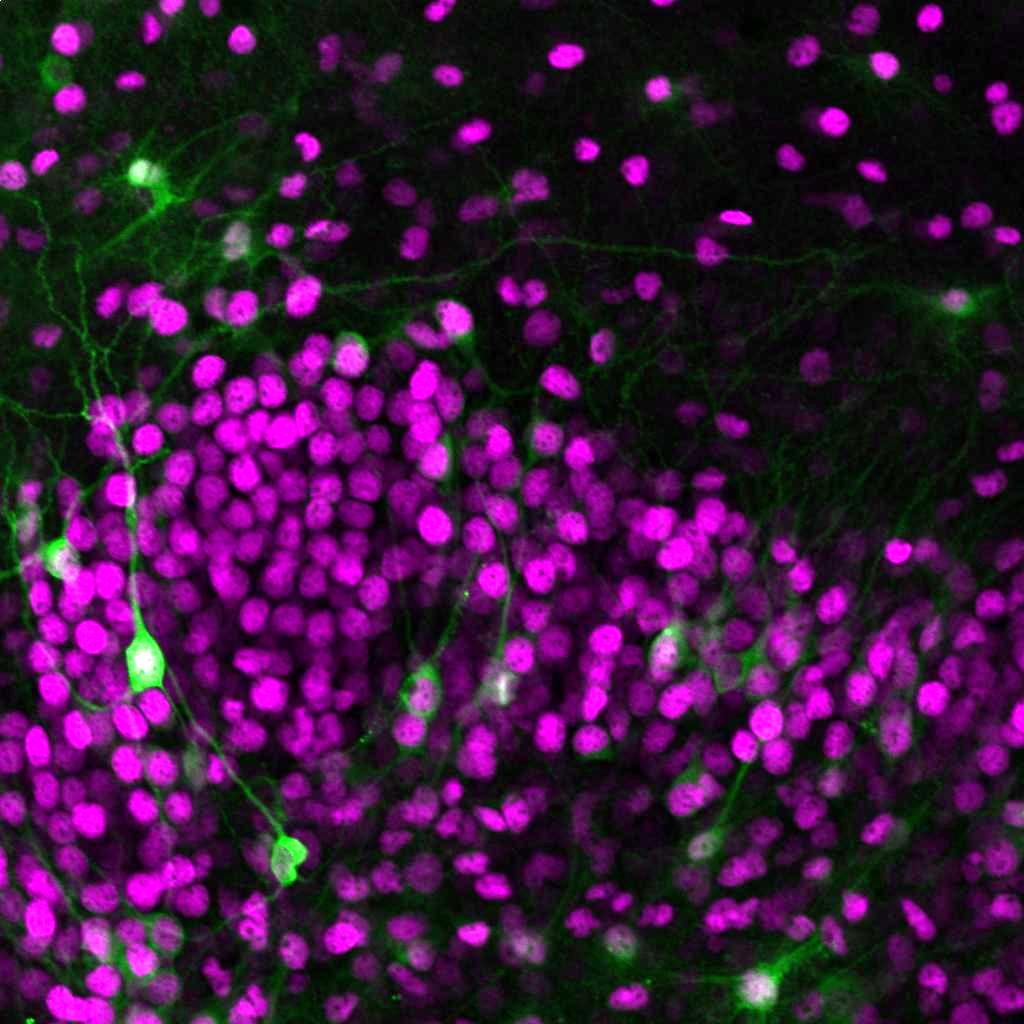

Supplement: Supplementary file 16 — Source Data [file 41467_2026_74968_MOESM16_ESM.zip › Source_data/Supplementary Fig2c_left.jpg]

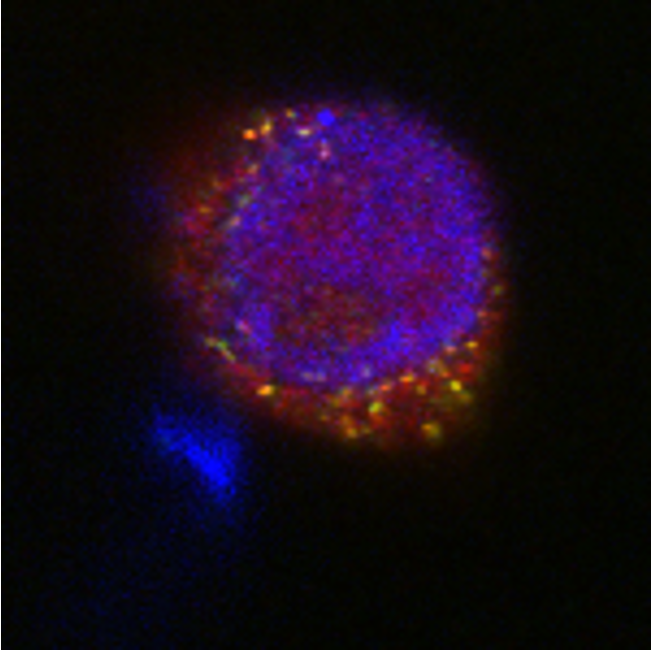

Supplement: Supplementary file 16 — Source Data [file 41467_2026_74968_MOESM16_ESM.zip › Source_data/Fig 5e_top right.png]

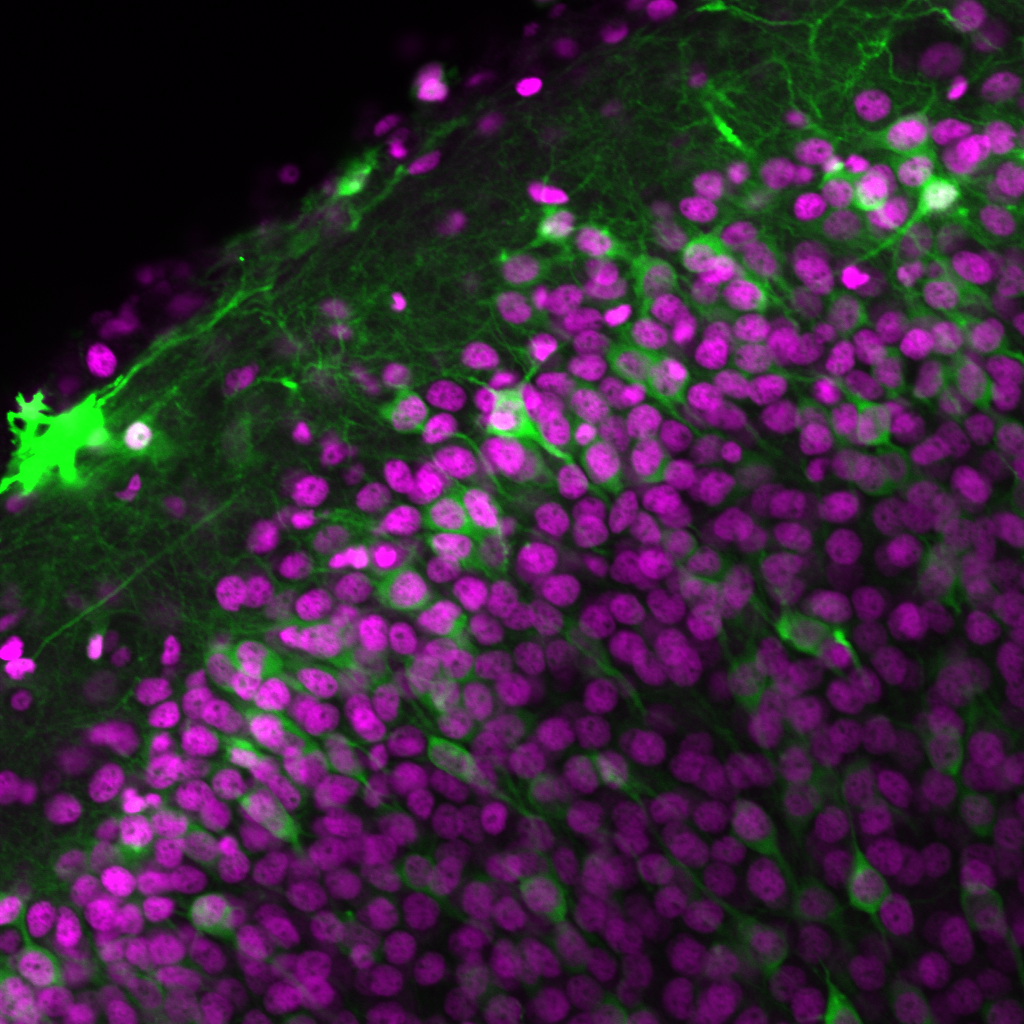

Supplement: Supplementary file 16 — Source Data [file 41467_2026_74968_MOESM16_ESM.zip › Source_data/Supplementary Fig2c_right.tif]

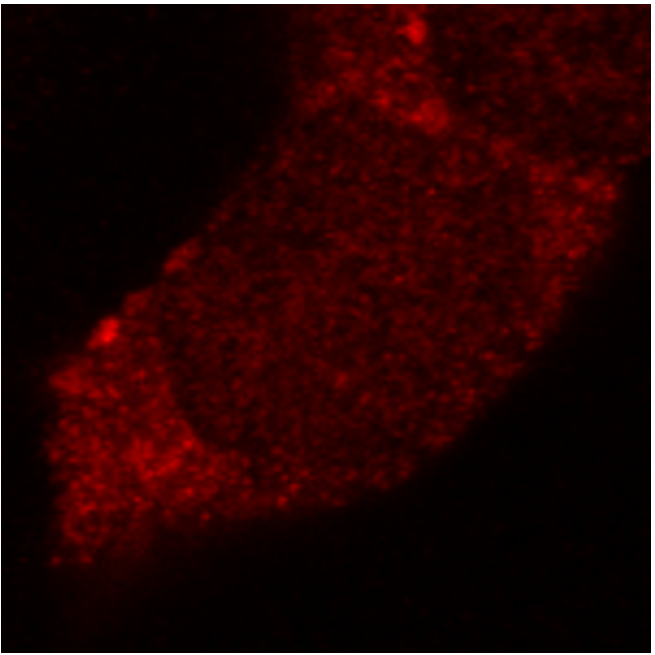

Supplement: Supplementary file 16 — Source Data [file 41467_2026_74968_MOESM16_ESM.zip › Source_data/Fig 5e_bottom middle.png]
