# Supplementary material for: Activity-dependent ribosome profiling reveals the landscape of canonical and non-canonical translation in brain tissue
Source: Nat Commun. 2026 Jul 23;17:6179. doi: 10.1038/s41467-026-74968-z (PMC13396407; doi:10.1038/s41467-026-74968-z)
Supplement: Supplementary file 15 — Transparent Peer Review file [file 41467_2026_74968_MOESM15_ESM.pdf]

nature portfolio

Peer Review File

Activity-dependent ribosome profiling reveals the landscape of canonical and non-canonical translation in brain tissue

Corresponding Author: Dr Motomasa Tanaka

This file contains all reviewer reports in order by version, followed by all author rebuttals in order by version.

Version 0:

Reviewer comments:

Reviewer #1

(Remarks to the Author)
In this manuscript, the Tanaka group developed a method that can selectively analyze the ribosome behaviors of the acutely activated neurons. This was achieved by the multiple selection steps; EGFP-10a incorporation into ribosomes, activity-dependent expression of EGFP nanobody-HALO, and the HALO immunoprecipitation. The obtained activity-dependent Ribo-seq data together with RNA-seq revealed neuronal activity-dependent translational upregulation and uORF translation.

The strength of this study is the development of the activity-dependent Ribo-seq technology that enables selective profiling of activated neurons for translation. However, the manuscript falls short of mechanistic insights.

Major comments

1. Page 7 “Moreover, the analysis of translational efficiency24 by comparing differential expression in activity-dependent Ribo-seq and RNA-seq in mORFs revealed distinct translational regulation upon neuronal activity (Extended Data Fig. 3a, b)” (lines 150-152)
If I understand correctly, the authors performed RNA-seq on total lysates from TTX and cLTP samples before the HALO-based immunoprecipitation using HA-GFP nanobody-HALO-PEST that binds to EGFP-L10a. In contrast, Ribo-seq was performed on immunoprecipitated ribosomes, presumably isolating transcripts only from activated neurons. This implies that the cLTP RNA-seq samples would contain a mixture of both resting and activated neurons, and perhaps even non-neuronal cells, whereas the cLTP Ribo-seq samples are enriched with activated neurons only. Given this discrepancy, would RNA-seq data obtained after HALO immunoprecipitation serve as a more appropriate condition for analyzing translation efficiency under cLTP treatment? Otherwise, the current translation efficiency analysis might overestimate the activity-dependent translation because the cLTP Ribo-seq data is expected to show larger difference from TTX than RNA-seq. If the authors believe that the current cLTP RNA-seq samples are appropriate for the goal of this study, it would be important to justify this choice and address the potential confound introduced by the cellular heterogeneity in the RNA-seq samples.

2. Page 8 “First, the classifier RF was trained on normalized expression counts of the top fifty significant genes from our activity-dependent Ribo-seq datasets, using the machine learning algorithm MLSeq29. Next, the normalized expression counts of the KCl-treated neurons RNA-seq samples were tested on the previously trained RF model (Fig. 2a)” (lines 180-183)
It is unclear why the authors chose to train the RF classifier using Ribo-seq data and then test it using RNA-seq data from the previous study. Do the authors assume that activity-dependent translation patterns are similar to the transcriptional patterns for this cross-modal classification to be valid? Given that the PCA plot already shows clear separation of the (presumably) Ribo-seq samples by condition (Figure 1d), it is unsurprising that the RF classifier performs well when trained on those same data. If the authors’ goal is to identify genes that contribute to determining neural activation status, examining the PCA loading could provide a more direct and interpretable approach. More importantly, using RNA-seq data for testing may undermine the authors’ conclusion that “This analysis further verified that our method is effective in capturing activity-dependent translation.” (lines 194-195), since the classifier was not evaluated within the same data modality. This could introduce a confounding issue due to differing biological signals between translation and transcription. The authors should either justify the rationale for using RNA-seq data as a test set for a classifier trained on Ribo-seq data or consider omitting this analysis altogether if it does not directly support their main claim.

3. Page 9 “A detailed analysis of differentially expressed ORFs upon neuronal activity indicated that nearly 50% of activity-dependent translation is non-canonical, suggesting that the genes other than canonical mORFs have a significant contribution to functional regulation by neuronal activity (Fig. 3b) (lines 207-210)
The pie chart in Fig. 3 showed that the majority of so-called “non-canonical” ORFs are internal ORFs. However, these may arise due to incomplete Ribo-seq coverage across the main ORFs. The authors’ claim that “the genes other than canonical mORFs have a significant contribution to functional regulation by neuronal activity” may need to be toned down.

4. Page 15 “Therefore, the technical platform developed in this study will open new avenues to reveal how neuronal activity-dependent translation impacts neuronal functions in health and disease.” (lines 342-344)
It would be helpful if the authors could briefly discuss the limitations of the current technology and study design. For example, does the multi-step protocol - including EGFP-L10a incorporation, activity-dependent expression of the EGFP nanobody-HALO construct, and subsequent HALO-based immunoprecipitation - reduce the overall RNA yield available for activity-dependent Ribo-seq? Additionally, does this method have sufficient sensitivity to detect translational changes under more physiological conditions, such as endogenous neuronal activation, rather than artificial stimulation like cLTP? A more detailed discussion of these technical considerations would enhance the manuscript and help readers evaluate the potential applications and limitations of this platform in future studies.

Minor comments

1. Page 7 “The slices from both groups were then subjected to immunoprecipitation using Halo-trap beads and processed for Ribo-seq (Fig. 1b)” (lines 137-138)
Could the authors provide quality control results of Ribo-seq such as read length distribution, in-frame ratios, and mapped regions (CDS, 5’UTR, and 3’UTR)?

2. Page 7 “To evaluate the efficacy of our method, we first examined differential gene expression between cLTP and control TTX-treated cultured hippocampal slices (Fig. 1c).” (lines 146-147)
Could the authors comment on why there are almost no downregulated mORFs in the comparison between cLTP and TTX?

3. Page 7 “The PCA analysis revealed that the TTX and cLTP sample groups showed well-separated clusters, indicating the underlying differences by neuronal stimulation (Fig. 1d).” (lines 156-158)
Is this PCA using RNA-seq, Ribo-seq, or Ribo-seq normalized by RNA-seq?

4. Page 9 “confusion metric” (line 191)
Maybe confusion matrix? A confusion metric is not widely used.

5. Figures 1b
Please add some explanations of the RNA- and Ribo-seq plots. What is transcript X? Is this a real data or just an example?
6. Figures 1f-i
The overlayed density plots of TTX and dLTP are hard to see. How about plotting seperatedly and changing the mORF annotation colors (currently both TTX and mORF are shown in gray)?
7. Extended Data Fig. 2d
The x tick labels and the corresponding legend need to be revised since they are impossible to understand. The main text (page 7, lines 141-143) needs to clearly explain that the data is from RiboTRAP RNA-seq analysis from a previous study.
8.Page 21 ”RNA-seq and Ribo-seq data will be deposited in the NCBI’s Gene Expression Omnibus database (GEO GSExxxx).” (lines 128-179)
The authors need to deposit their data to GEO and provide the reviewer’s token.

9. Page 21 ”All custom codes and pipelines are available upon request.” (lines 183-184)
I guess Nature Communication requires the authors to make the code publicly available.

Reviewer #2

(Remarks to the Author)
Suryawanshi et al describe a new method to selectively profile the translational activity of neurons that are engaged in plasticity events. They employ the technique to uncover new upstream ORFs that may have an important role in neuronal plasticity, such as the one preceding the main ORF of Egr1. The strongest feature of the manuscript is the development of an exciting tool to detect changes in the translatome of neurons that are part of an engram. To achieve this, the authors conditioned the expression of an anti-GFP nanobody to the E-SARE promoter, a synthetic construct activated upon neuronal activity. In parallel, neurons express RPL10a::eGFP, a ribosomal protein conjugated with eGFP that, once incorporated into the ribosomes, can be used as bait to immunopurify cell type-specific ribosomes. The problem addressed is a real bottleneck of the field, most studies to date either addressed global changes in selected neuronal sub-populations or used whole tissue lysate to study translation changes with codon resolution. The statistics were properly performed and there are no major concerns with the bioinformatic methods employed (minor comments included below). Overall, if the authors can provides some additional controls, the tool represents a unique instrument to characterize active neural populations in the brain that are incorporated in engrams, which should draw considerable attention from the community.

There are some concerns that should be addressed by the authors.

Major Comments

1) The main concern is the lack of benchmarking experiments against other techniques to truly demonstrate that the tool is a leap in the field. Without that, the claims that this tool is an advance are not supported. Because the new tool is the highlight of the paper, without these experiments there is little novelty that can be extracted from this particular work.

2) Another concern is how the story is presented. The authors perform interesting analyses demonstrating non-canonical translation of multiple mRNAs and superficially analyze the upstream ORFs that are translated in response to chemical LTP. There are many interesting findings here, but no validation experiments, making it difficult to assess the true value of the findings gathered. What are the micro-peptides resulting from uORF translation doing? I would strongly recommend characterizing at least the product of the uORF associated with the Egr1 mRNA, which was the case study presented in the manuscript. Functional evidence gathered from experiments such as acute exposure of slices to recombinant peptide encoded in the uORF should be presented.

3) The authors need to include the multiQC of ribosome-protected fragment libraries used on the manuscript. Metrics such as codon periodicity, average RPF length and percentage of RPFs mapped to the CDS should be presented in the manuscript. Furthermore, authors should clarify the percentage of reads lost at filtering steps performed prior to analyses. How many of these were rRNA?

Minor Comments

1) The experiment demonstrating that HaloTag-GFP nanobody expression is confined to activated neurons is not convincing. I would recommend comparing Halotag-GFP nanobody expression to some marker of activation, such as Arc or even using gCAMP.

2) The translation efficiency is computed against the whole RNA-sequencing, which encompasses both neural and non-neural reads. This can create damaging biases in the final analysis, where genes particularly enriched in glial cells show low translation efficiency in neurons, but only because there is a large input of transcriptome originated from glia.

3) It is unclear what Ext. Fig 2D means. What do the authors mean by “The neuron specificity of the activity-dependent Ribo-seq datasets aligned with the expression pattern of Arc, consistent with the design of the E-SARE promoter, which incorporates regulatory elements from the Arc gene (Extended Data Fig. 2d)” – lines 141-143. Is there any direct comparison between the expression of the nanobody and Arc?

4) Extended Fig 3a-b. There is no explanation as to how the authors defined each of the groups cited (forwarded, exclusive, buffered, intensified). Furthermore, this reviewer would suggest simplifying the description in the main manuscript (lines 146-160). The authors insert the description for each group between parentheses and insert some gene names in the text, which makes the reading difficult.

5) The story behind Gm13889 is potentially interesting, but it has no connection with the remaining of the manuscript, nor any biological validation proving that there is an alternative initiation site at ACG/T58. This reviewer thinks these findings are dispensable for the manuscript. Furthermore, there is no exploration regarding the other three genes cited, Spty2d1, Rasl11a and Pramel12, and they are not even cited for the remaining of the manuscript.

6) There is no reference in the text to panels Ext Fig 3d-f

7) Figure 2 – The supervised ML model performs accurately. This was predictable, given that the genes employed in the analysis are broadly known to be main components of neural activity, such as Egr2-4, Nr4a1-3, Dusp genes, Fos an Arc. It is important that the authors can reproduce previous data and reinforces their method as a substantial improvement towards studying “active” cells. But predicting cell state using these genes is hardly novel. Furthermore, immediate early genes are part of stress response. Therefore, even though the authors can use these genes to classify quiescent versus active cells, they most likely do not represent an activity signature, which is a much more complex and unsolved problem in neurobiology of plasticity.

8) Lines 234-235 – “This result indicates that activity-dependent translational changes in 5’UTR are primarily regulated at the transcriptional level.” The rationale behind this conclusion is reasonable, as in their data mRNAs that have high uORF translation also have changes at the transcriptional level. However, this finding is confusing, and the only logical explanation for changes in 5’UTR expression (based on the data presented) would be higher number of ribosomes loaded with these mRNAs. This either suggest a leaky system employed by the cell or that these uORFs are the “main targets” of translation during activity. This reviewer suggests comparing, within mRNAs that present high “forwarded” translation, what are the ratios of uORF vs mORF translation. Differences in this comparison might suggest that there is direct targeting of uORFs, rather than mORFs, for translation, and that this is not just a leaky system where more mRNAs transcribed lead to higher proportions of uORFs translated.

9) Figure panels are cited out of order on the text. For example, panels 3f and 3g are cited first on lines 231 and 232, where only two violin plots are cited. Then the authors cite the rest of the plot on lines 261-263, after describing figures 4 and 5. This difficult interpretation of the manuscript, and this reviewer thus suggests breaking the panels into two different plots.

10) Extended data 8A – The authors infer based on an N of 1. This data should be completed with additional experiments.

Reviewer #3

(Remarks to the Author)
I co-reviewed this manuscript with one of the reviewers who provided the listed reports. This is part of the Nature Communications initiative to facilitate training in peer review and to provide appropriate recognition for Early Career Researchers who co-review manuscripts.

Version 1:

Reviewer comments:

Reviewer #1

(Remarks to the Author)
In this revised manuscript, the authors have addressed many concerns by clarification and additional experiments. As a result, it is now suitable for publication.

Reviewer #2

(Remarks to the Author)
Major comments:

The authors made a sincere effort to reply to the comments of the two reviewers, which is appreciated. They produced a new figure for the manuscript, acknowledged that the computational portion of the work was lacking rigor, removed the ML section, and focused their attention on analyzing the Egr1-uORF as the sole case study in their dataset. The authors also attempted to include a cell type-specific bulk-IP-RNA-seq as a "transcriptome" control, which is far from representing the cell type-specific transcriptome but does add a bit more context to their findings as it allows the comparison of bulk-IP-RNA-seq ribosomal loading versus ribosomal localization data obtained from the ribo-seq.

Taken together, the authors' attempt to address the problems pointed out by the reviewers were not entirely satisfactory. Having said that, some of these problems (such as studying the cell type-specific transcriptome in parallel with the translatome) are still unsolved, which diminishes this reviewer's frustration.

The main strength of the manuscript is the tool developed by the authors. The biology discovered is interesting but needs refinement. This manuscript fits well as a novel tool description with some functional validation.

Minor comments:

The Gm13889 still feels out of place. I suggest removing it, but I will leave it to the editor's discretion.

Line 111: This is an over-the-top sentence. The tool is new, but a pioneering work usually encompasses a much more in-depth biological characterization. uORF-derived micropeptides have been extensively studied, as has uORF-based translational control in neuronal plasticity and translational changes induced by neuronal activity. I suggest toning down these type of statements. (this is a new request).

Line 144: In addition, the brain cell-type-specific expression analysis from Furlanis et al., 2019 demonstrates the enrichment of Arc in activity-prone CaMKII+ neurons (Fig S2g) – Furlanis et al., 2019 studies the resting landscape of the cell type-specific translatome. How do the authors get to "activity-prone" Camk2a+ neurons? All neuron types have the possibility of being active.

Line 247L: It could also mean post-transcriptional regulatory level.

The new experiments partially characterizing the peptide encoded by the Egr1-uORF are quite interesting. The characterization of the PTS domain and functional demonstration that it localizes the peptide to peroxisomes are convincing. Although I hoped for a demonstration of its role in the ISR or neuronal activity, I recognize that this was not the primary goal of the manuscript.

The discussion remains weak. The authors continue to describe findings in their discussion, rather than contextualizing their findings with what was previously found in the literature. There is also little speculation, which reduces the excitement about the findings.

Open Access This Peer Review File is licensed under a Creative Commons Attribution 4.0 International License, which permits use, sharing, adaptation, distribution and reproduction in any medium or format, as long as you give appropriate credit to the original author(s) and the source, provide a link to the Creative Commons license, and indicate if changes were made.
In cases where reviewers are anonymous, credit should be given to 'Anonymous Referee' and the source.
The images or other third party material in this Peer Review File are included in the article's Creative Commons license, unless indicated otherwise in a credit line to the material. If material is not included in the article's Creative Commons license and your intended use is not permitted by statutory regulation or exceeds the permitted use, you will need to obtain permission directly from the copyright holder.
To view a copy of this license, visit https://creativecommons.org/licenses/by/4.0/

REBUTTAL RESPONSE

We are pleased to resubmit our revised manuscript. We thank the reviewers for their insightful comments, which have allowed us to strengthen the study through new experimental data and refined analysis. Detailed point-by-point responses are provided in the accompanying letter. For clarity, revisions in the manuscript are highlighted in red, while new text in the response letter is marked in magenta. We believe these updates fully address the concerns raised and look forward to your further assessment.

Reviewer 1

(Remarks to the Author):
In this manuscript, the Tanaka group developed a method that can selectively analyze the ribosome behaviors of the acutely activated neurons. This was achieved by the multiple selection steps; EGFP-10a incorporation into ribosomes, activity-dependent expression of EGFP nanobody-HALO, and the HALO immunoprecipitation. The obtained activity-dependent Ribo-seq data together with RNA-seq revealed neuronal activity-dependent translational upregulation and uORF translation.

The strength of this study is the development of the activity-dependent Ribo-seq technology that enables selective profiling of activated neurons for translation. However, the manuscript falls short of mechanistic insights.

> We thank the Reviewer for this constructive feedback and for recognizing the innovation of our activity-dependent Ribo-seq technology. To address the request for deeper mechanistic insight, we have expanded the scope of our study by incorporating activated-neuron-specific RNA-seq (IP-RNA-seq) alongside a suite of complementary validation experiments.

By integrating these high-resolution datasets, we successfully decouple transcriptional contributions from translational control within the activated neuronal population. This multi-layered approach allowed us to provide a comprehensive mechanistic model illustrating how neuronal activity directs the synthesis of non-canonical proteins and uORFs to reshape the proteome. We believe these additions strengthen both the mechanistic depth and the translational relevance of the study.

Major comments

1. Page 7 "Moreover, the analysis of translational efficiency[24] by comparing differential expression in activity-dependent Ribo-seq and RNA-seq in mORFs revealed distinct translational regulation upon neuronal activity (Extended Data Fig. 3a, b)" (lines 150-152). If I understand correctly, the authors performed RNA-seq on total lysates from TTX and cLTP samples before the HALO-based immunoprecipitation using HA-GFP nanobody-HALO-PEST that binds to EGFP-L10a. In contrast, Ribo-seq was performed on immunoprecipitated ribosomes, presumably isolating transcripts only from activated neurons. This implies that the cLTP RNA-seq samples would contain a mixture of both resting and activated neurons, and perhaps even non-neuronal cells, whereas the cLTP Ribo-seq samples are enriched with activated neurons only. Given this discrepancy, would RNA-seq data obtained after HALO immunoprecipitation serve as a more appropriate condition for analyzing translation efficiency under cLTP treatment? Otherwise, the current translation efficiency analysis might overestimate the activity-dependent

translation because the cLTP Ribo-seq data is expected to show larger difference from TTX than RNA-seq. If the authors believe that the current cLTP RNA-seq samples are appropriate for the goal of this study, it would be important to justify this choice and address the potential confound introduced by the cellular heterogeneity in the RNA-seq samples.

> We appreciate the comment regarding the activated neuron-specific RNA-seq. We have fully addressed this concern with an additional set of experiments. First, however, there are a few important factors to be considered: (1) Even the sample for activated neuron-specific RNA-seq would not include free-floating mRNAs alone that are not engaged in translation, suggesting that the activated neuron-specific RNA-seq is also not an ideal control and may overestimate the activity-dependent translation in the activated-neuron-specific RNA-seq design. (2) Theoretically, the HALO IP strategy utilized for Ribo-seq is readily applicable for RNA-seq. However, we have found substantial technical challenges for the RNA-seq using HALO immunoprecipitation with brain samples. HALO beads would capture the ribosomes loaded onto mRNA, compared to those from Ribo-seq, where monosomes after the RNase treatment are to be pulled down. We found the capture of ribosomes onto mRNAs (polysome) by HALO beads was a bottleneck step, potentially due to the higher load of ribosomes in the polysome pulldown strategy. Alternative method of pelleting the ribosomes and fractionation of monosome/polysomes after sucrose density gradient prior to HALO IP, is also likely to reduce the pulldown efficiency because the precipitated ribosomes tend to form aggregates. Therefore, we argue that both the total RNA-seq and the RNA-seq from HALO IP have their inherent disadvantages and advantages. Thus, in the original study, we decided to leverage the first strategy of Bulk-RNA-seq as a control due to its robust and well-documented design.

However, in order to fully address the reviewer's concern, we performed a new RNA-seq experiment after HALO IP from the activated (cLTP) and the control (TTX) cultured hippocampal slices. Although, as expected, the yield of RNAs after HALO IP was very low, we overcame this issue of library preparation by leveraging the low-input tailored RNAs-seq kit as described in the method (SMART-Seq Ultra-low input RNA Kit), hereafter referred to as IP-RNA-seq. Since both IP- and Bulk-RNA-seq methodologies have their own advantages in terms of biological interpretation, we compared the similarity and differences between them, and found that the conclusions were largely consistent.

In the revised manuscript, we added the new results in the main text and figures in Fig. 1f: Translation efficiency (TE) from IP-Ribo-seq and IP-RNA-seq. The TE from IP-Ribo-seq and Bulk-RNA-seq analysis from the original manuscript, Fig. S3b, is now at Fig. S5b, and we have also noted the limitation of Bulk-RNA-seq in the main text (page 8, lines 169-183). Similarly, for the TE analysis focused on 5'UTR, we added the new results in the main text and figures in Fig. 2h: TE from IP-Ribo-seq and IP-RNA-seq. The TE from IP-Ribo-seq and Bulk-RNA-seq analysis from the original manuscript, Fig. 3h, is now at Fig. S7c (page 11, lines 242-246).

2. Page 8 "first, the classifier RF was trained on normalized expression counts of the top fifty significant genes from our activity-dependent ribo-seq datasets, using the machine learning algorithm MLSeq[29]. Next, the normalized expression counts of the KCl-treated neurons rna-seq samples were tested on the previously trained rf model (fig. 2a)" (lines 180-183). It is unclear why the authors chose to train the RF classifier using Ribo-seq data and then test it using RNA-seq data from the previous study**.** Do the authors assume that activity-dependent translation patterns are similar to the transcriptional patterns for this

cross-modal classification to be valid? Given that the pca plot already shows clear separation of the (presumably) ribo-seq samples by condition (figure 1d), it is unsurprising that the RF classifier performs well when trained on those same data. If the authors' goal is to identify genes that contribute to determining neural activation status, examining the PCA loading could provide a more direct and interpretable approach. More importantly, using RNA-seq data for testing may undermine the authors' conclusion that "this analysis further verified that our method is effective in capturing activity-dependent translation." (lines 194-195), since the classifier was not evaluated within the same data modality. This could introduce a confounding issue due to differing biological signals between translation and transcription. the authors should either justify the rationale for using RNA-seq data as a test set for a classifier trained on Ribo-seq data or consider omitting this analysis altogether if it does not directly support their main claim.

> We thank the reviewer for this important point. We acknowledge the validity of this concern regarding the cross-modal classification approach. We agree with the reviewers' concerns: Reviewer #1 pointed out that PCA loading could provide a more direct and interpretable approach for identifying genes contributing to neural activation status, and Reviewer #2 noted that immediate early genes are part of the stress response and thus may not represent a true activity-dependent signature (Reviewer #2 minor comment #7). In order to avoid potential confusion, we have removed the ML analysis from the revised manuscript.

We note that the primary rationale for using RNA-seq data as a test set was the lack of previously published Ribo-seq datasets with comparable read-depth, and that the ML analysis was restricted to IEGs among the top 50 differentially upregulated transcripts in IP-Ribo-seq, not representing the full scope of activity-dependent translation.

However, the reviewer #2 highlighted a distinct major concern (#1) about the need for benchmark experiments and analysis. In light of resolving these benchmarking concerns, we extended the RF classifier analysis to be trained on Bulk-Ribo-seq and compared it to that of IP-Ribo-seq. Notably, we found that IP-Ribo-seq data is more informative in terms of activity-dependent translation (Fig. R1). Please see reviewer #2, major comment #1 for additional details.

3. Page 9 "A detailed analysis of differentially expressed ORFs upon neuronal activity indicated that nearly 50% of activity-dependent translation is non-canonical, suggesting that the genes other than canonical mORFs have a significant contribution to functional regulation by neuronal activity (Fig. 3b) (lines 207-210) The pie chart in Fig. 3 showed that the majority of so-called "non-canonical" ORFs are internal ORFs. However, these may arise due to incomplete Ribo-seq coverage across the main ORFs. The authors' claim that "the genes other than canonical mORFs have a significant contribution to functional regulation by neuronal activity" may need to be toned down.

> We thank the reviewer for their feedback. Before addressing the main concern put forward by this reviewer, we want to clarify that, in the original manuscript, the labelled colour order of ORFs was not in sync between Fig. 3a and 3b of the original manuscript. The colours were appropriately assigned, but the order of plotting was different in Fig. 3a, 3b, and thereafter (original manuscript). To keep the flow consistent across the entire figure, we have changed the colours and colour order in Fig. 2a and 2b in the revised manuscript. This revised order of plotting was also extended to Fig. 2c and 2e of the revised manuscript.

This reviewer rightly asserted that the predicted ORFs detected in this study, including internal ORFs (supplementary table 1-9), may still have false positives due to incomplete Ribo-seq coverage across the main ORF and are fundamentally indistinguishable. Thus, in response to this reviewer's concern, we applied a more stringent biological filter to report representative ORFs that are differentially expressed in Figs. 3b, c, e of the original manuscript and Figs. 2b, c, e of the revised manuscript. The filter is as described in the RibORF publication/algorithm (Zhe Ji et al., Curr Protoc Mol Biol. 2018), with certain modifications: "Many candidate ORFs can overlap with each other with the same stop codon and different start codons. In this case, a representative ORF is selected based on the following criteria: we first pick AUG as a start codon if present, and we then choose the 5' most start codon as the representative one. Yet, if there is no read between the picked one and the next downstream candidate, we choose the next one as the representative start codon. Further, if more than one representative start codon is assigned for the ORFs ending with the same stop codon, then they were deemed as one by selecting the longest ORF as a representative." For the analysis in Figs. 3 b-c, e (original manuscript) and Figs. 2 b-c, e (revised manuscript), we now report representative ORFs that are differentially expressed upon neuronal stimulation ($|\log_2 FC|>0$, $P<0.05$). This filtering approach, as described in Zhe Ji. et al., *Curr Protoc Mol Biol.* 2018, would likely reduce false positive detections. As expected, this filtering strategy substantially reduced the number of differentially expressed ORFs, thereby decreasing the total number of internal ORFs identified. However, the percentage contribution remained largely similar. For example, uORFs were previously ~8% but now ~6%. This refined reanalysis yielded results consistent with our original findings, and accordingly, the conclusions remain unchanged.

We further want to clarify that the analysis focusing on uORFs, Fig. 3d onwards (original manuscript) and Fig. 2d onwards (revised manuscript), reports all predicted uORFs. Since uORFs are not derived from the incomplete coverage of the CDS, filtering only the representative ones is not necessary. Importantly, as stated in the original manuscript, our primary conclusions are based on robustly upregulated uORFs (baseMean > 100), which remain unchanged in the revised manuscript.

4. Page 15 "Therefore, the technical platform developed in this study will open new avenues to reveal how neuronal activity-dependent translation impacts neuronal functions in health and disease." (lines 342-344). It would be helpful if the authors could briefly discuss the limitations of the current technology and study design. For example, does the multi-step protocol - including EGFP-L10a incorporation, activity-dependent expression of the EGFP nanobody-HALO construct, and subsequent HALO-based immunoprecipitation - reduce the overall RNA yield available for activity-dependent Ribo-seq? Additionally, does this method have sufficient sensitivity to detect translational changes under more physiological conditions, such as endogenous neuronal activation, rather than artificial stimulation like cLTP? A more detailed discussion of these technical considerations would enhance the manuscript and help readers evaluate the potential applications and limitations of this platform in future studies.

> We appreciate the reviewer's attention to this technical aspect. First, the multi-step RNA selection process reduces the overall RNA yield, making *in vivo* library preparation challenging, and this point has been addressed in the discussion as per the reviewer's suggestions. We would like to mention that, although the number of activated neurons in response to behavioral tests will be substantially small, our future work involves developing low-input Ribo-seq that could be readily applied for *in vivo* experiments. We

now develop low-input activity-dependent Ribo-seq to be applied for *in vivo* activity-dependent translation analysis that reflects endogenous/physiological conditions.

Second, the time point scaling of the experiment is limited; the stability of GFP nanobody may reduce over time (Zhang Z. et al., *Sci Rep.* 2020), making it practically difficult to assess activity-dependent translation several hours after stimulation. Third, the effectiveness of GFP nanobody diffusion within activated neurons upon expression remains uncertain. Limited diffusion may enrich localization of GFP nanobody in the cell body rather than the axon and dendrites, potentially reflecting soma translation while under-representing local translation. Fourth, the cross-validation of novel activity-dependent uORFs using mass spectrometry presents inherent challenges. Peptides and small proteins are generally shorter in length and may have significantly shorter half-lives than canonical proteins. Additionally, they may have lower abundance or undergo rapid degradation by the proteasome. These features may limit their detection by mass spectrometry. For example, in the largest available study, Deutsch E. W. et al., *bioRxiv.* 2025, only 30 reliable ncORF peptides were identified out of 3.8 billion mass spectra from 95,520 experiments, suggesting that MS is inherently limited in its capacity for validation of novel activity-dependent uORFs. Moreover, Ribo-seq-based detection is unbiased towards the stability of the translational product unlike MS. While this limitation further emphasizes that IP-Ribo-seq can detect cryptic translation in response to neuronal stimulation, which is not amenable to detection by conventional techniques, we have duly acknowledged it.

We have updated these limitations in the Discussion section of the revised manuscript (page 16-17, lines 372-387).

Minor comments

1.Page 7 “The slices from both groups were then subjected to immunoprecipitation using Halo-trap beads and processed for Ribo-seq (Fig. 1b)” (lines 137-138) Could the authors provide quality control results of Ribo-seq such as read length distribution, in-frame ratios, and mapped regions (CDS, 5’UTR, and 3’UTR)?

> We thank the reviewer for the opportunity to clarify the library quality of our datasets. The Ribo-seq data follows standard footprints distribution and tri-nucleotide periodicity pattern. Additionally, the majority of reads are aligned to CDS, as expected. The data supporting these quality controls are included in Fig. 1c and Fig. S3 a-g in the revised manuscript. We have updated these results in the revised manuscript (page 7, lines 153-157).

We particularly bring the reviewers’ attention to the rRNA content in the Ribo-seq data. As mentioned in the revised methods, no rRNA depletion strategy was involved during library preparation. Skipping the rRNA depletion was intentional, since the original aim of this project was to investigate ribosomal heterogeneity using the rRNA fragments in the Ribo-seq datasets (Alkan et al., *Nuc Aci Res.* 2022). However, the analysis of this aspect remained inconclusive and thus was not further pursued in depth for the scope of this research. The rRNA content of our libraries (~70-80%) was comparable to previous brain Ribo-seq datasets (Duffy E. et al., *Nat. Neurosci.* 2022). These noncoding reads were subsequently removed during the data pre-processing step using the Bowtie alignment algorithm.

2.Page 7 "To evaluate the efficacy of our method, we first examined differential gene expression between cLTP and control TTX-treated cultured hippocampal slices (Fig. 1c)." (lines 146-147) Could the authors comment on why there are almost no downregulated mORFs in the comparison between cLTP and TTX?

> We thank the reviewer for this question. Although there are many activity-dependent downregulated genes such as Nrsn1, the downregulation is reported to be preferentially enriched immediately after neuronal stimulation (*i. e.* 5 min) (Cho J. et al., Science. 2015). In contrast, this study recorded translational changes 1 hour after neuronal stimulation. Therefore, we speculate that the differences might arise from the specificity of the time-point of the experiment. Additionally, as shown in Fig. 3c of the original manuscript and Fig. 2c of the revised manuscript (blue bar), ~100 downregulated transcripts are present in the canonical ORF category. This may not be immediately apparent in the volcano plot of the original manuscript (Fig. 1c). Since the volcano plot (Fig. 1c) apparently does not fully manifest the downregulated transcripts due to the scaling of the Y-axis, in the revised manuscript, we expanded the $-\log_{10}$(*P*-values) axis to better reflect downregulated genes colored in blue (Fig. 1d).

Of note, in the original manuscript, Fig. 1c–d utilized ComBat for batch correction, while the translational efficiency (ΔTE) analysis (Fig. 3h) employed a Generalized Linear Model (GLM) design formula (~batch + condition), which is the standard statistically appropriate approach for differential testing. Additionally, an R package *apeglm* was used for log2 fold-change shrinkage to ensure robust data visualization.

To ensure maximum clarity and methodological consistency for the reader, we have now standardized all DESeq2 analyses in the revised manuscript (including uORF and mORF/CDS datasets) to utilize the GLM-based batch correction. This includes Fig. 5a, where we replaced the initial bootstrapping approach- originally intended to mitigate low sample size (*n*=2) with the standard GLM framework.

Critically, this unified reanalysis shows that the significant gene sets remain largely unchanged. Our primary conclusions continue to be supported by the subset of uORFs that are robustly upregulated across all predicted candidates. Consequently, these improvements to the statistical workflow do not alter the fundamental findings of the study. The updated analyses are mapped as follows:

Original Fig. 1c–e (GSEA version 4.0.3) → Revised Fig. 1d, f, g (GSEA version 4.2.3)
Original Fig. 4 → Revised Fig. 3
Original Fig. 3d, f, g → Revised Fig. 2d, f, g
Original Extended Data Fig. 7 → Revised Fig. S10
Original Fig. 5a-c & 3f, g → Revised Fig. 4a-c & Fig. S9a, b

3.Page 7 "The PCA analysis revealed that the TTX and cLTP sample groups showed well-separated clusters, indicating the underlying differences by neuronal stimulation (Fig. 1d)." (lines 156-158) Is this PCA using RNA-seq, Ribo-seq, or Ribo-seq normalized by RNA-seq?

> We thank the reviewer for this question. Raw counts from Ribo-seq (now referred to as IP-Ribo-seq in the revised manuscript) were processed using DESeq2 for the PCA. We have added the details to the revised main text (page 8, lines 166-168) and updated the figure legend of the revised manuscript.

4.Page 9 "confusion metric" (line 191) Maybe confusion matrix? A confusion metric is not widely used.

> We appreciate the reviewer's careful observation. In the revised manuscript, we have corrected this to "confusion matrix".

5.Figures 1b Please add some explanations of the RNA- and Ribo-seq plots. What is transcript X? Is this a real data or just an example?

> We have revised the schematics in Fig. 1b to be more intuitive. We have revised the schematic to include all RNA-based library preparation methods applied in this study. Activity-dependent Ribo-seq, also referred to as HALO pull-down-based Ribo-seq upon cLTP treatment, is hereafter referred to as "IP-Ribo-seq". The corresponding benchmark control Ribo-seq upon cLTP from whole hippocampal slices is referred to as "Bulk-Ribo-seq." Similarly, the HALO pull-down based RNA-seq upon cLTP and control RNA-seq from whole hippocampal slices upon cLTP are hereafter referred to as "IP-RNA-seq" and "Bulk-RNA-seq," respectively.

The data shown in Fig. 1b is from the Nr4a2 mRNA and was derived from actual experimental results (IP-Ribo-seq) and used as a representative example. The gene symbol is not explicitly mentioned in Fig. 2b, since the goal of this figure is to show methodology. Thus, we originally used the symbol "Transcript X" as a notation. In the revised manuscript. We have changed the figure legend to explicitly state that the transcript is Nr4a2. However, this may give readers of the journal an impression that this study is related only to the Nr4a2 gene. Thus, we used the notation "(e. g.)" on the X- axis, in addition to Nr4a2."

We have updated the schematic in Figure 1b in response to the experimental suggestions provided by Reviewers #1 and #2.

6.Figures 1f-I The overlayed density plots of TTX and cLTP are hard to see. How about plotting seperatedly and changing the mORF annotation colors (currently both TTX and mORF are shown in gray)?

> We agree with the reviewer and have updated the plots to visualize TTX and cLTP separately. Although these data are omitted from the revision based on the concern raised by reviewer #2, minor comment #5, we extended the correction suggested by this reviewer to all the read density plots included in the manuscript. The list of plots is given below:

Fig. 1f (original manuscript) = Fig. S6a (revised manuscript)

Fig. 4e (original manuscript) = Fig. 3d (revised manuscript)

Fig. 5d-f (original manuscript) = Fig. 4d-f (revised manuscript)

7.Extended Data Fig. 2d The x tick labels and the corresponding legend need to be revised since they are impossible to understand. The main text (page 7, lines 141-143) needs to clearly explain that the data is from RiboTRAP RNA-seq analysis from a previous study.

>We appreciate the reviewer's attention. The main text is updated to clarify that the data is from RiboTRAP RNA-seq analysis from a previous study (Furlanis E. et al., *Nat Neurosci.* 2019). Briefly, CX and HC refer to the mouse neocortex and hippocampus, respectively. CamK2-cre: Excitatory glutamatergic pyramidal neurons, neocortical projection neurons, and hippocampal CA1 pyramidal neurons. Scnn1a-cre: Layer 5 intratelencephalic (IT) excitatory neurons in neocortex. Grik4-cre: Hippocampal CA3 pyramidal excitatory neurons. SST-cre: Somatostatin-expressing inhibitory interneurons. Pvalb-cre: Parvalbumin-expressing inhibitory interneurons. VIP-cre: Vasoactive intestinal peptide-expressing inhibitory interneurons. In the revised manuscript, we have clarified the labels in the figure legend and included an explanatory text to acknowledge that this data

is a reanalysis of the previous study from Furlanis E. et al., *Nat Neurosci.* 2019 (page 7, lines 143-145).

8.Page 21 ”RNA-seq and Ribo-seq data will be deposited in the NCBI’s Gene Expression Omnibus database (GEO GSExxxx).” (lines 128-179). The authors need to deposit their data to GEO and provide the reviewer’s token.

>We thank the reviewer for pointing it out. We deposited all the RNA-seq and Ribo-seq data under the GEO accession number GSE317977. The reviewer-only access is now possible from the token given below:

Token: qtsryyguhnspjaz

The proteomics data from the Egr1-uORF immunoprecipitation experiment (reviewer #2 major comment #2) is accessible at PXD074666. The reviewer-only access is now possible from the token given below:

https://repository.jpostdb.org/preview/3915594816997e5138f481

Token: 2365

9.Page 21 ”All custom codes and pipelines are available upon request.” (lines 183-184) I guess Nature Communication requires the authors to make the code publicly available.

> We thank the reviewer for pointing this out. All custom code and analysis pipelines mentioned in the original manuscript were previously published and were replicated here for the analyses. Although no new code was developed in this study, we have listed the preprocessing commands and general analysis packages in the following link:

https://github.com/nayanvs/Suryawanshi_et_al._2026

We have included this information in the Method of the revised manuscript

Reviewer 2

(Remarks to the Author)
Suryawanshi et al describe a new method to selectively profile the translational activity of neurons that are engaged in plasticity events. They employ the technique to uncover new upstream ORFs that may have an important role in neuronal plasticity, such as the one preceding the main ORF of Egr1. The strongest feature of the manuscript is the development of an exciting tool to detect changes in the translatome of neurons that are part of an engram. To achieve this, the authors conditioned the expression of an anti-GFP nanobody to the E-SARE promoter, a synthetic construct activated upon neuronal activity. In parallel, neurons express RPL10a::eGFP, a ribosomal protein conjugated with eGFP that, once incorporated into the ribosomes, can be used as bait to immunopurify cell type-specific ribosomes. The problem addressed is a real bottleneck of the field, most studies to date either addressed global changes in selected neuronal sub-populations or used whole tissue lysate to study translation changes with codon resolution. The statistics were properly performed and there are no major concerns with the bioinformatic methods employed (minor comments included below). Overall, if the authors can provides some additional controls, the tool represents a unique instrument to characterize active neural populations in the brain that are incorporated in engrams, which should draw considerable attention from the community.
> We thank the Reviewer for their supportive assessment and for accurately highlighting the 'bottleneck' our study aims to address. We are gratified that the Reviewer recognizes the potential of this technology to characterize active neural populations and engrams.

To address the request for additional controls and to further demonstrate the necessity of this activated-neurons-specific approach, we have significantly expanded our comparative framework. Specifically, we have incorporated conventional Bulk-Ribo-seq (performed upon cLTP without activated-neuron specificity) to serve as a direct benchmark against our activity-dependent method. Beyond technological benchmarking, to provide functional validation, we performed an interactome analysis of the Egr1-uORF, providing a first look at the protein-protein interactions of this novel activity-dependent product.

We believe these additions reinforce the study's methodological rigor and biological depth. We hope these new controls and functional insights satisfy the Reviewer's request for a more robust validation of the tool.

Major comments

1.The main concern is the lack of benchmarking experiments against other techniques to truly demonstrate that the tool is a leap in the field. Without that, the claims that this tool is an advance are not supported. Because the new tool is the highlight of the paper, without these experiments there is little novelty that can be extracted from this particular work.

> We thank the reviewer for his/her suggestion of the benchmarking experiment to demonstrate the advancement of the proposed method of capturing activity-dependent translation in this study. In light of this suggestion, we performed new benchmarking experiments with whole brain slices (TTX vs. cLTP) without HALO immunoprecipitation and compared them side by side for differentially expressed genes using cluster-based analysis using the *pheatmap* package in R algorithm. As shown in Fig. S4 of the revised manuscript, cluster #1 of gene expression, which is predominantly enriched for neuronal genes (panel b), shows more enrichment of activity-dependent expression in IP-Ribo-seq

data (Column 1, panel a) than the Bulk-Ribo-seq data (Column 2, panel a), demonstrating the advancement of the proposed tool. We have updated these results in the revised manuscript (page 7, lines 158-162).

We additionally compared RF classifier model performance trained on IP-Ribo-seq data or Bulk-Ribo-seq data to investigate if the IP-Ribo-seq data-based model shows higher accuracy, which may further support that the IP-Ribo-seq method surpasses conventional methods to represent activity-dependent translation (Fig. R1). Briefly, for both IP- and Bulk-Ribo-seq, IEGs among the top 50 transcripts were selected for training (Fig. R1a). 34 transcripts out of the top 50 (~68%) were IEGs in IP-Ribo-seq. In contrast, only 21 transcripts (~42%) were IEGs in Bulk-Ribo-seq, with reference to Tyssowski et al., *Neuron*. 2018. Although the RF classifier based on Bulk-Ribo-seq performed better compared to the IP-Ribo-seq during training (Fig. R1 c), during testing, the model trained on IP-Ribo-seq had higher overall accuracy (Fig. R1 d-j). Additionally, the model trained on Bulk-Ribo-seq had a further decline in performance during testing, when the threshold was relaxed to include more than 21 IEGs in the RF classifier. In conclusion, the IP-Ribo-seq-based RF classifier model (**$P = 0.0021$) performed better than the Bulk-Ribo-seq-based RF classifier (*$P = 0.012$). P-values were obtained by permutation testing.

In response to the original concerns raised by both reviewers regarding the machine learning (ML) analysis in the main manuscript, we have decided to remove it from the main text. To allow close scrutiny, we have deposited the code on the GitHub page associated with this publication (see Reviewer #1, Minor Comment #9).

Method: Machine learning-based prediction of neuronal activity status using Random Forest model

For both IP-Ribo-seq and Bulk-Ribo-seq, IEGs among the top 50 differentially upregulated transcripts upon neuronal stimulation were selected. Corresponding normalized and batch-corrected RPF counts of each transcript were extracted. The classifier analysis was performed using the R package MLSeq. We implemented the random forest model referring to its previous application in RNA-seq (Goksuluk et al., 2019; Smyth et al., 2024; Wenric & Shemirani, 2018). The classifier was trained on a total of 6 randomly sorted TTX and cLTP-treated activated-neurons specific Ribo-seq replicates from this study, with ~80-20% train-test splits. During the training process, all models underwent 5-fold cross-validation, which was repeated 10 times. The trained model was tested using the scaled data of targeted RNA-seq of IEGs from Tyssowski et al. (Tyssowski et al., 2018). The reads derived from cultured neurons treated with either 60-minute KCl (n=7) or control (n=13), were tested. The testing accuracy, precision, recall, F1 score, and AUC/ROC were reported. The gene importance, internally generated by the classifier, was analyzed using their Mean Decrease Gini, MDG. We used 60 min time points for both the train-test split.

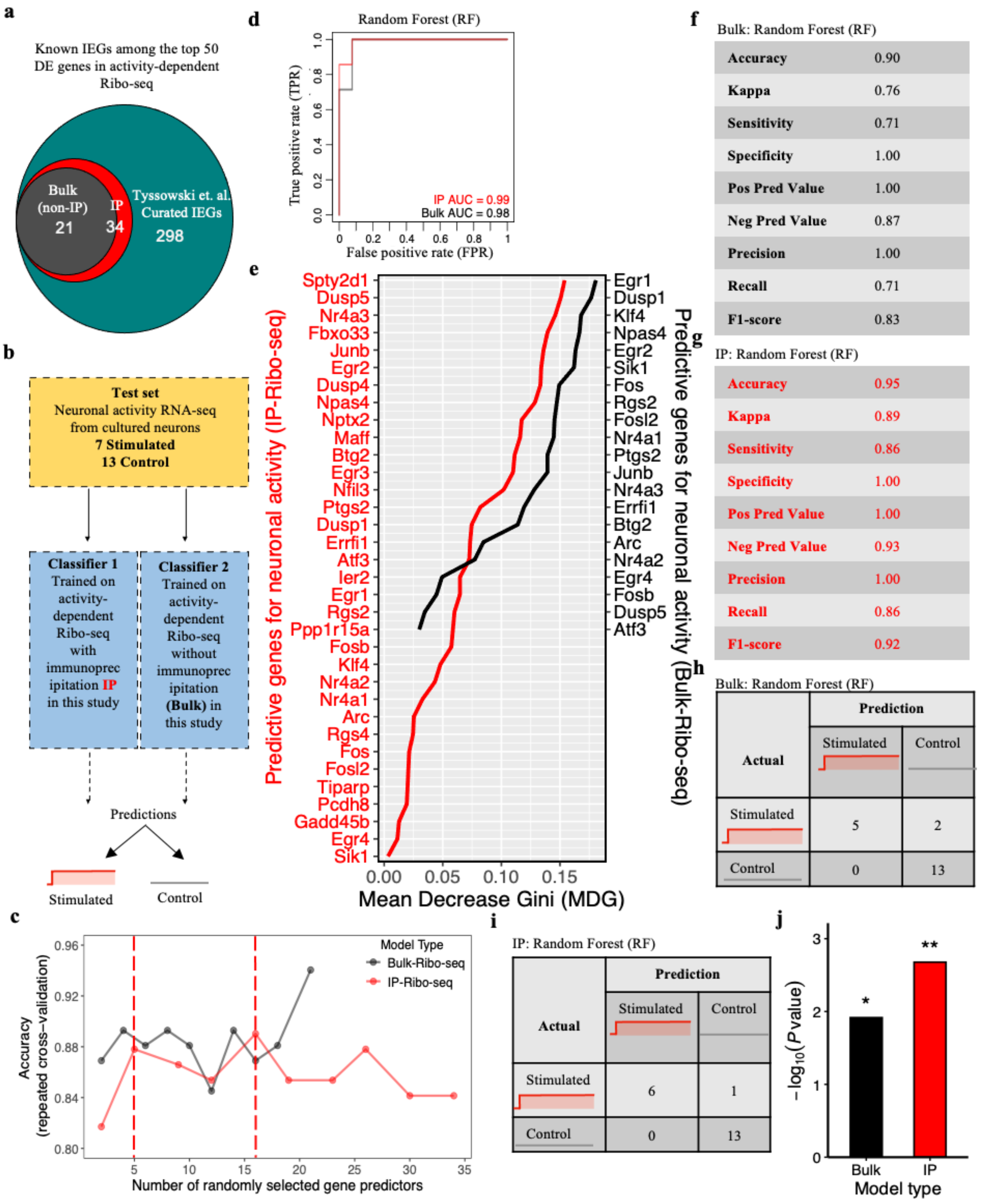

Fig. R1 | Machine learning-based validation of a method to investigate activity-dependent translation. a, *Venn diagram showing the proportion of known immediate early genes (IEGs) among the top 50 differentially expressed transcripts in response to neuronal stimulation, from IP-Ribo-seq and Bulk-Ribo-seq.* ***b,*** *Overview of classifiers trained on six ex vivo activity-dependent gene expression Ribo-seq datasets from this study (3 TTX and 3 cLTP treated samples each). Classifier 1: IP-Ribo-seq; Classifier 2: Bulk-Ribo-seq. The trained models were used to infer activity status from 20 in vitro cultured neuronal RNA-seq datasets from Tyssowski et al. (13 control and 7 1h KCl treated samples).* ***c,*** *Accuracy*

metric of the random forest (RF) model in predicting neuronal activity status. The number of transcripts plotted on the x-axis represents randomly selected predictors/features. Accuracy is based on randomly selected transcript features from the training data (TTX and cLTP, n = 3) after 5-fold cross-validation repeated 10 times for Classifier 1 (IP-Ribo-seq, red) and Classifier 2 (Bulk-Ribo-seq, black). Dotted red lines at 5 and 16 indicate the best subset of transcript features for Classifier 1, yielding the highest prediction accuracy. ***d,*** *Receiver operating characteristic (ROC) analysis for Classifier 1 (IP-Ribo-seq, red) and Classifier 2 (Bulk-Ribo-seq, black), depicting the performance of the RF model in accurately identifying test samples.* ***e,*** *Mean Decrease Gini (MDG) metric showing ranked importance of gene features used by the RF model for Classifier 1 (red) and Classifier 2 (black) to accurately predict neuronal activity in unseen datasets.* ***f-g,*** *RF performance metrics highlighting classifier efficacy: Classifier 1 (IP-Ribo-seq, red, f) and Classifier 2 (Bulk-Ribo-seq, black, h) in accurately predicting neuronal activity in unseen datasets.* ***h-i,*** *Confusion matrices for RF models: Classifier 2 (Bulk-Ribo-seq, g) and Classifier 1 (IP-Ribo-seq, i) predicting neuronal activity status of test datasets from Tyssowski et al. Significance:* ***P*** *< 0.01, determined by the binomial test.* ***j,*** *Comparison of classification performance between Classifier 1 (IP-Ribo-seq) and Classifier 2 (Bulk-Ribo-seq). Classifier 1 shows superior performance. P-values were obtained by permutation testing of class labels and reflect the significance of classification accuracy relative to chance.*

2.Another concern is how the story is presented. The authors perform interesting analyses demonstrating non-canonical translation of multiple mRNAs and superficially analyze the upstream ORFs that are translated in response to chemical LTP. There are many interesting findings here, but no validation experiments, making it difficult to assess the true value of the findings gathered. What are the micro-peptides resulting from uORF translation doing? I would strongly recommend characterizing at least the product of the uORF associated with the Egr1 mRNA, which was the case study presented in the manuscript. Functional evidence gathered from experiments such as acute exposure of slices to recombinant peptide encoded in the uORF should be presented.

> We thank the reviewer for their feedback. Indeed, the goal of the study should be to provide biological advancement, in addition to the methodological advancement proposed in the original manuscript. Using the activated-neurons specific Ribo-seq, this study reports dozens of novel activity-dependent peptides or small proteins with unknown functions. However, we agree with the reviewer that an example uORF with functional evidence should be presented to demonstrate the potential of this study. In light of the suggestion by this reviewer, we have performed an additional set of experiments to characterize the product from *Egr1*-uORF.

This reviewer kindly suggested the experiment of treating cultured slices or neurons with a recombinant peptide of *Egr1*-uORF. However, our preliminary search using the computational prediction of cell-penetrating peptides (Kumar N. et. al. *J. Chem. Inf. Model.* 2025) suggested that exogenous delivery of the recombinant *Egr1*-uORF peptide may not be readily incorporated into the cells. Thus, this experimental direction was not pursued further.

In order to validate Egr1-uORF expression, we thoroughly investigated MS data downloaded from 4 or more previous publications (e. g., PXD036064, PXD025201, PXD008526, PXD050222, PXD009920, PXD006382). However, since the Egr1 uORF is

a short peptide with 27 amino acids, and due to the absence of K/R, there is only one peptide to be identified by MS analysis, representing a major challenge in detection. Additionally, as elaborated in the reviewer #1 major comment #4 (limitation #4, page 5 in this response letter), the stability of the peptide may influence detection by MS, unlike Ribo-seq.

We attempted to complement MS detection by developing a polyclonal antibody against Egr1 uORF, which could inform us about the subcellular localization of Egr1 uORF and potentially elucidate its function (Fig. R2 a). However, despite our substantial efforts, it showed background signals, and the development of the Egr1 uORF antibody was deemed unsuccessful.

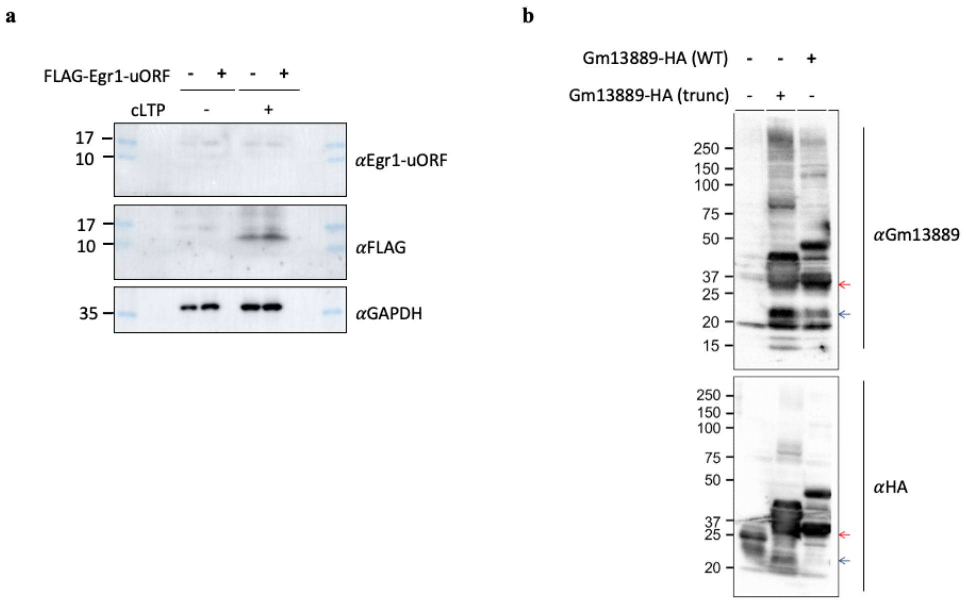

Fig. R2 | Lab-made antibody of Egr1-uORF and Gm13889 have low-specificity for the respective protein. a, *αEgr1-uORF western blot using cultured neurons transfected with or without FLAG-Egr1-uORF and with or without stimulation using cLTP. The FLAG antibody detected the bands of interest that appeared to be (10 kD). However, the same band was not detected by the endogenous Egr1-uORF antibody.* ***b,*** *αGm13889 western blot using HEK cells transfected with truncated Gm13889-HA (T58/ACG) or WT Gm13889-HA (M1/ATG), and HEK cells that have not been transfected with anything as a negative control. The HA antibody detected the bands of interest that appeared to be truncated (21.3 kD; blue arrow) and WT (27.6 kD; red arrow).*

Therefore, we investigated the function of *Egr1*-uORF by fluorescence microscopy and immunoprecipitation-based mass spectrometry. We overexpressed FLAG-tagged Egr1-uORF in primary cultured neurons, and examined its subcellular localization (Fig. 5e of the revised manuscript) and performed interactome analysis using IP-mass spectrometry (Fig. 5a of the revised manuscript). Immunostaining experiments indicated a punctate pattern in the cytosol (Fig. 5e). Notably, the IP-MS data identified many peroxisomal genes as top hits of interacting partners (Fig. 5b). Computational prediction of peroxisomal targeting sequence 1 (PTS1) using a previously published webtool (Neuberger G. et al., *J*

Mol. Biol. 2003) revealed that Egr1-uORF is a strongly predicted peroxisomal localizing small protein (Fig. 5d). In the conserved C-terminal region of the Egr1-uORF sequence, we revealed that the last 3 amino acids represent a canonical peroxisomal targeting sequence. Generally, the last three amino acids are the determinant of peroxisomal targeting; therefore, to validate peroxisomal localization, we additionally examined the subcellular localization of FLAG-Egr1-uORF Δ3aa mutant by deleting the conserved C-terminal 3 aa (Fig. 5e). Importantly, we observed a partial loss of distinct punctate structures and peroxisomal localization of the FLAG-Egr1-uORF Δ3aa mutant. Together, these results suggest Egr1 uORF has a novel role in peroxisomal biology. The characterization of the Egr1-uORF serves as a representative example of the broader class of activity-dependent translation products identified in this study. These data reveal a previously underappreciated landscape of small proteins and non-canonical ORFs within the nervous system. While a comprehensive functional analysis of every candidate is beyond the scope of the present study, these findings provide a rich foundation for future investigation into neuronal translational control. We included these results in Fig. 5 and updated the main text of the revised manuscript (page 14, lines 314-333).

Since these experiments broadly implied that peroxisomal biology is linked to neuronal activity in a manner partially resembling ISR, we surveyed the literature linking peroxisomal biology, neuronal activity, and ISR. One study by Abe Y et. al. *Front. Cell Dev. Biol.* 2020, suggests that peroxisomes are required for memory formation. Another study by Kim J. et. al. *PNAS Nexus.* 2025, suggested that peroxisomal import stress triggers ISR. Although these studies do not focus on translational regulation, Kim J. et. al. performed RNA-seq upon peroxisomal import stress from HEK cells (Fig. S13a). Interestingly, our new analysis revealed that ~16% of activity-dependent mORFs were significantly upregulated upon peroxisomal import stress (e. g., *ARC* and *FOS*) (Fig. S13b), and these shared genes are involved in the regulation of the MAPK pathway (Fig. S13c). Furthermore, although our primary objective was to investigate the role of the Egr1 uORF in peroxisomal biology, we unexpectedly identified that the human ortholog of the previously uncharacterized IEG *Gm13889*, *C11orf96* (Fig. 1f, original manuscript) is also upregulated upon peroxisomal import stress (Fig. S13a). We have included these links in the Discussion section of the revised manuscript (page 16, lines 360-371). These findings are also referred to in our response to Reviewer #2, minor comment #5.

3.The authors need to include the multiQC of ribosome-protected fragment libraries used on the manuscript. Metrics such as codon periodicity, average RPF length and percentage of RPFs mapped to the CDS should be presented in the manuscript. Furthermore, authors should clarify the percentage of reads lost at filtering steps performed prior to analyses. How many of these were rRNA? Is this PCA using RNA-seq, Ribo-seq, or Ribo-seq normalized by RNA-seq?

> We agree with the reviewer. In the revised manuscript, we included multiQC reports in Fig. 1c and Figs. S3 a-g, which met general quality standards. As further described in the reviewer #1 minor comment #1, the library did have high rRNA fragment content, which was strategically retained for ribosomal heterogeneity analysis upon neuronal activity. However, the results of ribosomal heterogeneity remained inconclusive, and therefore, we did not report the results in this manuscript.

Minor comments

1.The experiment demonstrating that HaloTag-GFP nanobody expression is confined to activated neurons is not convincing. I would recommend comparing Halotag-GFP nanobody expression to some marker of activation, such as Arc or even using gCAMP.

> We thank the reviewer for this important comment.

HaloTag-GFP nanobody expression in our system is driven by the E-SARE promoter, a validated activity-dependent promoter (Kawashima et al., *Nat. Methods*. 2013). Thus, by design, HaloTag-GFP nanobody expression should be restricted to activated neurons.

To experimentally confirm this, we compared HaloTag-GFP nanobody expression with the activity marker *c*-Fos in hippocampal slice cultures following cLTP induction (Figs. S2 a-d of the revised manuscript). *c-Fos* is a well-established immediate early gene induced by neuronal activation and regulated independently of the synthetic E-SARE promoter, making it suited for this validation experiment. Our immunohistochemical analysis showed that 83% (dentate gyrus) and 86% (CA1) of NeuN-positive neurons were c-Fos-positive after cLTP, and importantly, 94% (dentate gyrus) and 95% (CA1) of HaloTag-GFP nanobody-positive neurons were also c-Fos-positive, demonstrating that HaloTag expression is highly restricted to activated neurons.

Thus, these results provide direct experimental evidence that HaloTag-GFP nanobody expression driven by E-SARE is highly specific to activated neurons under our experimental conditions.

2.The translation efficiency is computed against the whole RNA-sequencing, which encompasses both neural and non-neural reads. This can create damaging biases in the final analysis, where genes particularly enriched in glial cells show low translation efficiency in neurons, but only because there is a large input of transcriptome originated from glia.

> We appreciate the comment regarding the translation efficiency analysis. As described in the reviewer #1 major comment #1, we have updated our datasets with an additional set of experiments to address the concerns related to translation efficiency (TE). We have also described the advantages and disadvantages of each method and the corresponding results. The TE results and conclusions remained largely consistent, potentially because the translational data for both analyses were based on the activated neuron-specific IP-Ribo-seq. We argue that both Bulk- and IP-RNA-seq provide crucial insights on their own and provide a rich resource for the reader of the journal. Therefore, we have included both datasets in the revised manuscript.

3.It is unclear what Ext. Fig 2D means. What do the authors mean by "The neuron specificity of the activity-dependent Ribo-seq datasets aligned with the expression pattern of Arc, consistent with the design of the E-SARE promoter, which incorporates PROMOTER AND ENHANCER from the Arc gene (Extended Data Fig. 2d)" – lines 141-143. Is there any direct comparison between the expression of the nanobody and Arc?

> We agree with the reviewer's concern. The design of the E-SARE promoter is based on the *Arc* gene enhancer and promoter (Fig. R3), which provided us with a rationale for comparing the expression patterns between IEGs such as Arc and the GFP nanobody expressed under E-SARE in Fig. S2d and Fig. S2g of the original and revised manuscripts, respectively. As described in our response to minor comment #1 above, the direct comparison of *Arc* and E-SARE-driven gene of interest has already been reported in Fig. 2a of the previous publication (Kawashima et al., 2013). Additionally, we have provided immunostaining data of another IEG, *c-Fos*, that has a similar expression profile to *Arc*, as

described in minor comment #1. We have further clarified this point in the figure S2g legend.

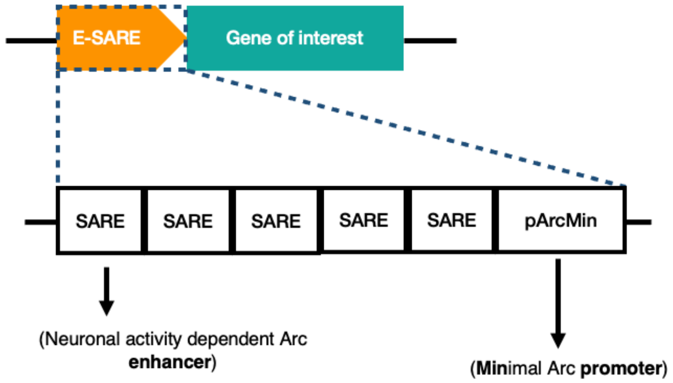

Fig. R3 | Design of the synthetic activity-dependent promoter E-SARE. *The enhancer region of the Arc gene, known as the synaptic activity–responsive element (SARE), is concatenated into five repeats and fused with the essential region of the Arc promoter to drive several-fold stronger activity-dependent, neuron-specific expression compared with known endogenous activity-dependent promoters. The design was first developed by Kawashima, et al. Nat Methods 2013.*

4.Extended Fig 3a-b. There is no explanation as to how the authors defined each of the groups cited (forwarded, exclusive, buffered, intensified). Furthermore, this reviewer would suggest simplifying the description in the main manuscript (lines 146-160). The authors insert the description for each group between parentheses and insert some gene names in the text, which makes the reading difficult.

> We agree with the reviewer's comment. The definition of these groups was based on previous publications described in minor comment #6 below, and is updated in the references of the revised manuscript. We understand that the text is difficult to read due to the combination of terms, definitions, and examples. Thus, in the revised manuscript, we have moved the information about examples from each group to the respective figures and figure legends, while terms with their definitions are described at their first instance. The revised text is as follows (page 8, lines 173-175):

"…We categorized the differentially regulated genes into four distinct groups based on the relationship between mRNA abundance and translational output as described previously. These include forwarded (both translation up and mRNA up); intensified (translation up and mRNA down or vice versa); buffered (either translation changes negated by mRNA changes or vice versa), and exclusive genes (either translation up or mRNA up)…"

The description related to the revised figures and figure legends with the highlighted examples can be found in our response to reviewer #1's major comment #1 (last paragraph).

Of note, in response to the concerns regarding the translation efficiency (TE) analysis, we have incorporated additional panels referring to the four distinct groups

(forwarded, exclusive, buffered, intensified), into the revised manuscript. New panels utilizing IP-RNA-seq-based TE analysis for the CDS and 5'UTR are now presented in Figs. S5d and S7e, respectively. Consequently, the original Bulk-RNA-seq-based TE analyses for these regions have been moved to Figs. S5c and S7d. This reorganization allows for a direct comparison between the two methodologies.

5.The story behind Gm13889 is potentially interesting, but it has no connection with the remaining of the manuscript, nor any biological validation proving that there is an alternative initiation site at ACG/T58. This reviewer thinks these findings are dispensable for the manuscript. Furthermore, there is no exploration regarding the other three genes cited, Spty2d1, Rasl11a and Pramel12, and they are not even cited for the remaining of the manuscript.

> We thank the reviewer for their feedback. Indeed, the identification of the truncated version of *Gm13889* is one of the new contributions of this manuscript. Moreover, *Spty2d1*, *Rasl11a,* and *Pramel12* are other genes identified in this study with uncharacterised functions in the nervous system. We agree with the reviewer that exploration and/or biological validation should be presented to demonstrate the potential of this study. In light of this reviewer's suggestion, we have performed an additional set of experiments to validate *Gm13889*, *Spty2d1*, *Rasl11a,* and *Pramel12*.

For *Gm13889*, we attempted to develop a polyclonal antibody, which could help identify its truncated proteoforms and potentially elucidate its function (Fig. R2 b). However, despite our substantial efforts, it showed background signals, and the development of the *Gm13889* antibody was deemed unsuccessful.

Therefore, we performed a mass proteomics experiment upon cLTP in cultured hippocampal slices (Supplementary Table 10 of the revised manuscript). Three out of four previously uncharacterized IEGs proposed based on IP-Ribo-seq data in this study, namely *Gm13889*, *Spty2d1*, and *Rasl11a,* were detected using MS. We also found that all proteotypic peptides derived from Gm13889 were downstream of the alternative initiation site at ACG/T58, predicted from IP-Ribo-seq data (Fig. R4). Although the absence of any peptides from the N-terminal of full-length Gm13889 by MS does not fully validate the alternative initiation site, the data are consistent with the primary prediction from IP-Ribo-seq, which has single-nucleotide resolution. Notably, the N-terminal region is enriched in lysine and arginine residues, resulting in frequent tryptic cleavage and the generation of short peptides that are poorly detectable by LC-MS/MS, which may also account for the lack of N-terminal peptide identification. We have updated these results in the revised manuscript (page 9, lines 189-191).

Additionally, as described in our response to major comment #2 above (last paragraph), we found that the human ortholog of *Gm13889*, *C11orf96,* was also upregulated upon peroxisomal import stress, which triggers ISR. Interestingly, a previous study has concluded that *C11orf96* may be involved in ISR (Yang H. et al., *BMC Vet. Res.,* 2022).

However, in light of the feedback from this reviewer, the data corresponding to the mRNA of *Gm13889* was moved to the Fig. S6 of the revised manuscript. Furthermore, we have decided to eliminate the read density expression data corresponding to the other three mRNAs: *Spty2d1*, *Rasl11a,* and *Pramel12* (Fig. 1g-i of the original manuscript), but still highlighted in the volcano plot (Fig. 1c and Fig. 1d of the original and revised manuscripts,

respectively). Accordingly, we have updated the Results and Methods sections to include the new MS analysis in the revised manuscript.

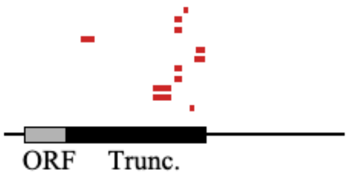

Fig. R4 | Related to supplementary table 10: Prototypic peptides of Gm13889 detected by MS upon neuronal stimulation. *In-depth MS analysis of cultured hippocampal slices upon cLTP (n=2). Gray and black box indicates the main ORF (mORF) and truncation, respectively. Each stacked Red line corresponds to the unique peptide of Gm13889 detected by MS.*

6.There is no reference in the text to panels Ext Fig 3d-f

> We thank the reviewer for pointing this out. We have included the appropriate references in the revised manuscript at their first instance.

1. Liu, Y. Li, E.E. Stackpole, A. Novak, Y. Gao, Y. Zhao, X. Zhao, & J.D. Richter , Regulatory discrimination of mRNAs by FMRP controls mouse adult neural stem cell differentiation, Proc. Natl. Acad. Sci. U.S.A. 115 (48) E11397-E11405
2. O. Larsson, N. Sonenberg, & R. Nadon, Identification of differential translation in genome wide studies, Proc. Natl. Acad. Sci. U.S.A. 107 (50) 21487-21492
3. McManus, C. Joel, et al. "Ribosome profiling reveals post-transcriptional buffering of divergent gene expression in yeast." Genome research 24.3 (2014): 422-430.
4. Chothani, S. et al. deltaTE: Detection of Translationally Regulated Genes by Integrative Analysis of Ribo-seq and RNA-seq Data. Curr Protoc Mol Biol 129, e108 (2019).

7.Figure 2 – The supervised ML model performs accurately. This was predictable, given that the genes employed in the analysis are broadly known to be main components of neural activity, such as Egr2-4, Nr4a1-3, Dusp genes, Fos an Arc. It is important that the authors can reproduce previous data and reinforces their method as a substantial improvement towards studying "active" cells. But predicting cell state using these genes is hardly novel. Furthermore, immediate early genes are part of stress response. Therefore, even though the authors can use these genes to classify quiescent versus active cells, they most likely do not represent an activity signature, which is a much more complex and unsolved problem in neurobiology of plasticity.

> We thank the Reviewer for this insightful feedback. We agree that the concerns regarding the machine learning (ML) analysis are valid; consequently, as noted in our response to Reviewer #1 (Major Comment #2), we have removed this analysis from the main manuscript.

However, to address the benchmarking concerns raised in Major Comment #1, we have performed an extended ML analysis comparing IP- and Bulk-Ribo-seq for the Reviewer's

consideration (Fig. R1). This repurposed analysis now serves a benchmarking function rather than a validation claim. We have deposited the code on the associated GitHub page for further review.

8.Lines 234-235 – “This result indicates that activity-dependent translational changes in 5’UTR are primarily regulated at the transcriptional level.” The rationale behind this conclusion is reasonable, as in their data mRNAs that have high uORF translation also have changes at the transcriptional level. However, this finding is confusing, and the only logical explanation for changes in 5’UTR expression (based on the data presented) would be higher number of ribosomes loaded with these mRNAs. This either suggest a leaky system employed by the cell or that these uORFs are the “main targets” of translation during activity. This reviewer suggests comparing, within mRNAs that present high “forwarded” translation, what are the ratios of uORF vs mORF translation. Differences in this comparison might suggest that there is direct targeting of uORFs, rather than mORFs, for translation, and that this is not just a leaky system where more mRNAs transcribed lead to higher proportions of uORFs translated.

> We thank the reviewer for this insightful suggestion. We agree that comparing uORF to mORF translation within forwarded transcripts would clarify whether translation targeting is likely regulated or attributable to leaky scanning. We have performed the differential uORF activity analysis as described in uORF-tools (Scholz A. et al., *PLoS*. 2019), with certain modifications as follows: Parameter 1. Not only uORF but also overlapping uORFs and N-terminally extended proteoforms contribute to the forwarded population in 5’UTR-specific translation efficiency analysis (Fig. 3h and Fig. 2h of the original and revised manuscripts, respectively). Thus, these 5’UTRs were removed from further analysis unless if they contain any uORFs. Parameter 2. To account for the multiple uORFs that are derived from each distinct 5’UTR among the forwarded translation, we applied a more stringent biological filter as described in detail in reviewer #1 major comment #3 and consolidated the uORFs ending with the same stop codon into a representative uORF during comparison across replicates and conditions. Parameter 3. If the uORF was not expressed in a particular replicate, then a pseudo-count of the lowest normalized expression in that replicate, divided by 2, was assigned to avoid division by zero errors. Parameter 4. These candidates were evaluated for differential uORF activity using their normalized expression (RPKM) as described in uORF-tools (REF). Parameter 5. Differential uORF activity within an empirically defined interval of $\pm$ 0.3 was classified as potential leaky scanning. Conversely, uORFs with median activity scores above 0.3 or below -0.3 were classified as translationally activating (CDS/mORF-up) or repressing (CDS/mORF-down), respectively.

As shown in Fig. S8a of the revised manuscript, we found a total of 17 and 9 uORFs among the forwarded 5’UTRs, which are likely translationally activating (CDS/mORF-up) or repressing (CDS/mORF-down), respectively. Meanwhile, 4 uORFs had median activity scores close to zero, suggesting potential leaky scanning. We also extended this analysis to the data reanalyzed from Cho. J. et. al., to cross-validate these predicted differential uORF activity, and found an overlapping set of translationally activating (*n*=4) and repressing uORFs (*n*=4), across these studies. We also note that a greater number of predicted leaky scanning uORFs were identified from our analysis of the Cho J. et al. dataset (*n* = 12) compared to the IP-Ribo-seq dataset central to this study (*n* = 4). This difference may reflect variations in read depth arising from the lack of activated-neuron specificity in the

Cho et al. dataset relative to the IP-Ribo-seq approach. We have updated these results in the revised manuscript (page 11, lines 248-257).

Of note, we want to bring this reviewer's attention to the differential uORF activity analysis of Egr1-uORF, performed in the original manuscript (Fig. 5g), where parameter 3 was not applied. In TTX conditions, certain replicate(s) had no detectable expression from IP-Ribo-seq and thus were originally assigned a zero value. In the revised manuscript (Fig. 4g), a pseudo count was assigned if the uORF was undetected in certain replicate(s), as described in parameter 3 above. Importantly, this improved reanalysis drew conclusions consistent with the results in the original manuscript, and thus, the conclusions remain unchanged.

In the revised manuscript, we include the new results in Figs. S8a and 4g. Fig. 4g in the revised manuscript is updated from Fig. 5g of the original manuscript. The main text is updated accordingly.

9.Figure panels are cited out of order on the text. For example, panels 3f and 3g are cited first on lines 231 and 232, where only two violin plots are cited. Then the authors cite the rest of the plot on lines 261-263, after describing figures 4 and 5. This difficult interpretation of the manuscript, and this reviewer thus suggests breaking the panels into two different plots.

> We apologize for the lack of clarity in the original figure presentation. Following the Reviewer's suggestion, we have separated these datasets into distinct plots to enhance readability and logical flow.

Specifically, we have refined the 'Other' 5'UTR control group to ensure a more rigorous comparison. In the original manuscript, this category excluded both activity-dependent and ISR-dependent uORFs. In the revised version, the 'Other' category is specifically tailored to each plot: it excludes 5'UTRs containing activity-dependent uORFs in Fig. 2f–g (page 10, lines 236-242), and those containing ISR-dependent uORFs in Fig. S9 (page 12, lines 275-283).

Additionally, as noted in our response to Reviewer #1 (Minor Comment #2), we have standardized the DESeq2 analysis across the manuscript using a unified batch-correction method. Because our analysis of 5'UTR length and stability is contingent upon the set of significantly upregulated uORFs, these violin plots have been updated to reflect the refined results.

This reanalysis remains entirely consistent with our original findings. The corresponding text has been updated to reflect these separated panels and to ensure a more intuitive narrative.

10. Extended data 8A – The authors infer based on an N of 1. This data should be completed with additional experiments.

> We appreciate the reviewer's concern. We have increased the sample size to n=3 for these experiments through additional replicates. We included the updated results in the Fig. S11a of the revised manuscript.

REBUTTAL RESPONSE

We are pleased to resubmit our revised manuscript. We thank the reviewers and editors for their critical evaluation and appreciating the potential and impact of the method is neurobiology research in coming years. Detailed point-by-point responses are provided in the accompanying letter. For clarity, revisions in the manuscript are highlighted in red, while new text in the response letter is marked in magenta. We believe these updates fully address the concerns raised and look forward to your further assessment.

Reviewer 1

(Remarks to the Author):
In this revised manuscript, the authors have addressed many concerns by clarification and additional experiments. As a result, it is now suitable for publication.
> We thank the Reviewer for recognizing the innovation of our activity-dependent Ribo-seq technology.

Reviewer 2

(Remarks to the Author)

Major comments

1. The authors made a sincere effort to reply to the comments of the two reviewers, which is appreciated. They produced a new figure for the manuscript, acknowledged that the computational portion of the work was lacking rigor, removed the ML section, and focused their attention on analyzing the Egr1-uORF as the sole case study in their dataset. The authors also attempted to include a cell type-specific bulk-IP-RNA-seq as a "transcriptome" control, which is far from representing the cell type-specific transcriptome but does add a bit more context to their findings as it allows the comparison of bulk-IP-RNA-seq ribosomal loading versus ribosomal localization data obtained from the ribo-seq.
> We thank the Reviewer for his/her comments and evaluating our new data in the revised manuscript.

2. Taken together, the authors' attempt to address the problems pointed out by the reviewers were not entirely satisfactory. Having said that, some of these problems (such as studying the cell type-specific transcriptome in parallel with the translatome) are still unsolved, which diminishes this reviewer's frustration.
> We thank the Reviewer for understanding our efforts for activated-neurons specific transcriptome analysis in the revised manuscript.

3. The main strength of the manuscript is the tool developed by the authors. The biology discovered is interesting but needs refinement. This manuscript fits well as a novel tool description with some functional validation.
> We thank the Reviewer for recognizing the potential of our activity-dependent Ribo-seq technology and the functional validation of Egr1-uORF. As the reviewer indicated, the

biology discovered in this study is interesting and provides opportunities for further studies on activity-dependent uORFs that remain unknown in neurobiology.

Minor comments

1. The Gm13889 still feels out of place. I suggest removing it, but I will leave it to the editor's discretion.

> We thank the Reviewer for this feedback. Gm13889 truncation is provided as an example of previously unrecognised activity-dependent translation product identified by our new Ribo-seq technique. We have decided to maintain them in Supplementary Fig. 6. We hope the Reviewer's understanding.

2. Line 111: This is an over-the-top sentence. The tool is new, but a pioneering work usually encompasses a much more in-depth biological characterization. uORF-derived micropeptides have been extensively studied, as has uORF-based translational control in neuronal plasticity and translational changes induced by neuronal activity. I suggest toning down these type of statements. (this is a new request).

> We thank the Reviewer for this feedback. We have rephrased the line 111 from the revised manuscript.

3. Line 144: In addition, the brain cell-type-specific expression analysis from Furlanis et al., 2019 demonstrates the enrichment of Arc in activity-prone CaMKII+ neurons (Fig S2g) – Furlanis et al., 2019 studies the resting landscape of the cell type-specific translatome. How do the authors get to "activity-prone" Camk2a+ neurons? All neuron types have the possibility of being active.

> We find the Reviewer's concern valid. However, Furlanis et al., 2019 studies did not employ any treatment to ensure neuronal silencing. Therefore, the data represent the combined population of resting neurons and spontaneously activated neurons. A general consensus is that *Arc* expression is largely conditional to neuronal activity. Therefore, the enrichment of Arc mRNA levels in (Fig S2g) can be, at least in part, attributed to activity-prone nature of CaMKII+ neurons, owing to its selective enrichment in the specific type of neurons.

4. Line 247L: It could also mean post-transcriptional regulatory level.

> We agree with the reviewer's concern. As the Reviewer indicated, the positive increase in ribosome density in 5'UTR along with the corresponding mRNA levels can be due to the post-transcriptional regulation such as increased stability of uORF-containing mRNAs or their reduced mRNA decay. However, transcriptional upregulation is very well known to be triggered by neuronal activity, and the evidence implying post-transcriptional regulation is limited. Since the possibility of post-transcriptional regulation cannot be ruled out, we have toned down the sentence as mentioned below:

Original manuscript:

This result indicates that activity-dependent translational changes in 5'UTR are **primarily** regulated at the transcriptional level.

Revised manuscript:

This result indicates that activity-dependent translational changes in 5’UTR are **likely** regulated at the transcriptional level.

5. The new experiments partially characterizing the peptide encoded by the Egr1-uORF are quite interesting. The characterization of the PTS domain and functional demonstration that it localizes the peptide to peroxisomes are convincing. Although I hoped for a demonstration of its role in the ISR or neuronal activity, I recognize that this was not the primary goal of the manuscript.

> We appreciate the Reviewer for his/her interest in elucidating roles of the peptide encoded by Egr1-uORF. Although we agree that the role of the peptide encoded by Egr1-uORF in ISR or neuronal activity would be a good addition, a functional elucidation of such newly identified microproteins warrants its own independent research. In response to this reviewer’s comment, we updated the discussion as mentioned below:

..the identification of previously unrecognised proteins in this study, warrants further research for their function in ISR and neuronal activity.

6. The discussion remains weak. The authors continue to describe findings in their discussion, rather than contextualizing their findings with what was previously found in the literature. There is also little speculation, which reduces the excitement about the findings.

> We thank the reviewer for their helpful feedback. In the Supplementary Fig. 1b-d and Fig 1d, we had compared our activity-dependent Ribo-seq data with some of the previously published datasets, showing that our technique provided rich activity-dependent translatome data. Additionally, as described in our previous manuscript, we had performed extensive analysis for many previously published Ribo-seq and MS datasets in order to compare the functional attributes of Egr1-uORF and truncated Gm13889. As we showed in Fig. 4e and Supplementary Fig. 1d of the revised manuscript, we revealed the ISR-dependent Egr1-uORF expression and the basal expression of truncated Gm13889 in rat hippocampal neurons, respectively. We believe that future studies would allow a fair evaluation of the methodology by regarding it as a benchmark and contextualizing the findings.
